# Diverse Microbial Communities Assemble on Both Recalcitrant and Labile Carbon Sources

**DOI:** 10.1111/1462-2920.70351

**Published:** 2026-06-25

**Authors:** Kaumudi H. Prabhakara, Yansong Zhao, Kristian Ullrich, Andrew D. Farr, Paul B. Rainey

**Affiliations:** ^1^ Department of Microbial Population Biology Max Planck Institute for Evolutionary Biology Plön Germany; ^2^ Laboratory of Biophysics and Evolution, CBI, ESPCI Paris, Université PSL, CNRS Paris France

**Keywords:** biomass, diversity, functional inference, interactions, microbial ecology, taxonomic dynamics

## Abstract

Microbial community assembly is shaped by the nature of available resources, with labile carbon sources like glucose often expected to support low diversity due to rapid growth and competitive exclusion. In contrast, recalcitrant substrates like cellulose are thought to support higher diversity through slower growth and increased niche partitioning. In previous work, we showed that compost‐derived microbial communities propagated on cellulose maintained high diversity over nearly a year. To determine whether such diversity is specific to recalcitrant substrates or reflects more general features of assembly, we tracked community dynamics in three environments—cellulose paper, cellulose broth and glucose—using daily 16S rRNA profiling. Communities maintained through four bi‐weekly serial transfers, with five replicates per treatment, yielded a high‐resolution dataset of over 800 samples. Despite originating from the same inoculum, communities diverged sharply in both taxonomic and functional composition. Cellulose environments yielded stable communities enriched in specialists, while glucose environments exhibited rapid succession and dominance by generalists. Surprisingly, all environments sustained comparably high levels of taxonomic diversity. Functional inference suggested extensive cross‐feeding and resource salvaging in both cases. Our results reveal distinct assembly trajectories under simple carbon regimes and provide a foundation for future mechanistic study.

## Graphical abstract

Taxonomically and functionally distinct communities assemble on cellulose and its monomer, glucose. Although single carbon sources were provided, high and comparable diversity was maintained in both environments, supported by extensive cross‐feeding and scavenging. However, glucose supported higher biomass than cellulose and selected generalists, while specialist bacteria were selected in cellulose.

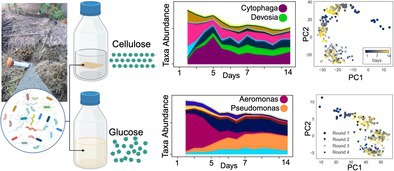

## 1 Introduction

Microbial communities underpin a wide range of ecological and industrial processes, performing complex functions through networks of interacting taxa (Kuypers et al. 2018; Buchan et al. 2014; D'Argenio and Salvatore 2015; Clemente et al. 2012; Melkonian et al. 2023; Werner et al. 2011; Meijer et al. 2026). A central challenge in microbial ecology is to understand how such communities assemble: the determinants of their structure, stability and diversity (Bittleston 2024; Gopalakrishnappa et al. 2022). Of particular interest is how distinct communities emerge from a shared inoculum when exposed to different environmental contexts (Goldford et al. 2018; Blanton et al. 2016; Prabhakara and Kuehn 2023). While synthetic communities have provided useful mechanistic insights (Leeuwen et al. 2023), they often lack the ecological complexity of natural systems (Cordero and Datta 2016; Gralka et al. 2020; Morin et al. 2022; Gallardo‐Navarro et al. 2024; Sanchez‐Gorostiaga et al. 2019; Mickalide and Kuehn 2019). By contrast, natural communities are rich and complex but difficult to track and manipulate (Louca et al. 2018; Vepštaitė‐Monstavičė et al. 2024; Rain‐Franco et al. 2024; Zhao et al. 2024; Yang et al. 2025). Propagating complex communities in controlled conditions offers a productive middle ground (Meijer et al. 2026; Prabhakara and Kuehn 2023; Astacio et al. 2021; Quistad et al. 2020).

In previous work (Quistad et al. 2020), we propagated microbial communities from garden compost on cellulose for nearly a year and observed sustained high taxonomic diversity, with over 200 genera persisting through regular (bi‐weekly) serial transfer. This led us to ask whether such diversity is a function of the resource—cellulose, a complex and recalcitrant polymer—or a more general feature of microbial community dynamics. To address this, we undertook a comparative study of community assembly on cellulose and glucose, the monomeric unit of cellulose, expecting the simpler substrate to support less diverse communities dominated by few rapidly growing taxa.

The results were unexpected. While community structure and dynamics differed markedly across environments, all three—glucose, cellulose broth and cellulose paper—supported high levels of taxonomic diversity. Cellulose‐grown communities exhibited slow, stable colonization dominated by specialists, whereas glucose‐grown communities showed rapid turnover, high biomass and a shifting cast of generalists and nitrogen‐fixing taxa. Functional inference pointed to cross‐feeding and niche construction as important features in both cases. Here we present a detailed account of these dynamics, based on daily 16S rRNA profiling over 8 weeks, with bi‐weekly serial transfers and five replicates per treatment—yielding a dense time series of over 800 samples. Although the mechanisms sustaining diversity remain to be fully resolved, the data reveal distinct ecological trajectories shaped by carbon source complexity and provide a foundation for mechanistic exploration of diversity maintenance in microbial ecosystems.

## 2 Materials and Methods

More detailed descriptions are found in Appendix A.

### 2.1 Sample Collection, Propagation and DNA Extraction

About 50 g compost was collected from a garden at $54^{{}^{\circ}}09^{\prime }32.4^{\prime \prime }\;N$ and $10^{{}^{\circ}}26^{\prime }13.2^{\prime \prime }\;E$ on 2 November 2021, 20 g of which was vigorously shaken with 100 mL M9 solution and then left static for ∼5 min for the solid particles to sediment. Five microcosms with 190 mL M9 medium and 40 cm^2^ cellulose paper (9 pieces of 2 cm × 2 cm paper and 16 pieces of 0.5 cm × 0.5 cm paper), and five microcosms with 190 mL M9 medium and 0.2 % w/v glucose, were each inoculated with 2 mL supernatant of the compost wash. The amount of cellulose (and separately) glucose added to each microcosm was adjusted in order to ensure an equivalent amount of carbon supplied in the two environments. Both environments had one microcosm each as control, that is, they were not inoculated with the compost. All microcosms were statically incubated at 28°C for 14 days. Every day during incubation, 2 mL of broth from all microcosms and one piece of 0.5 cm × 0.5 cm paper from the cellulose microcosms, were harvested. The DNA in the samples were immediately extracted using Qiagen PowerSoil Pro kit and then quantified using Qubit dsDNA High Sensitivity kit.

**FIGURE 1:**
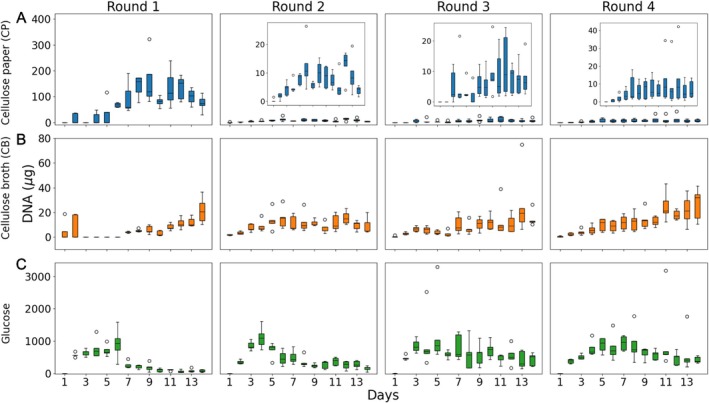
Community growth dynamics. The total DNA concentration (in μg) is plotted on the *y*‐axis over the 14‐day growth period (*x*‐axis) across four sequential Rounds (columns). Each boxplot represents the values across five replicate communities in each environment, where the boxes extend from the first to the third quartile, solid line represents the median, and the whiskers extend from the box to the farthest data point lying within 1.5 times the inter‐quartile range. (A) The DNA concentration for communities on cellulose paper (CP), (B) for communities in cellulose broth (CB) and (C) for communities in glucose. Insets in (A) show rescaled *y*‐axes to highlight lower DNA concentrations in cellulose environments.

The quantified DNA was then scaled up to obtain the total DNA content of the communities (Figure 1). The DNA concentration was first multiplied by the volume of elution (here 50 μL) to get the DNA eluted. For the liquid samples, the eluted DNA was divided by volume used for extraction (2 mL) and multiplied by the volume of the culture to estimate the DNA content of the entire microcosm. For the cellulose paper samples, since the DNA was harvested from 0.25 cm^2^ paper, the eluted DNA was divided by 0.25 cm^2^ and multiplied by 40 cm^2^, the total area of the paper in the culture, to estimate the total DNA content on the paper.

At the end of the 14‐day incubation, 1/20 of the content of each microcosm was transferred to a new, sterile microcosm with the same resource type and amount. The 1:20 ratio is a compromise between two aims: to lose as little diversity as possible at each transfer and to allow substantial new growth and thus change in diversity patterns. Ten microlitres of the broth was used to inoculate the next microcosm. In addition, for cellulose microcosms, nine 2 cm × 2 cm pieces of paper were harvested and vortexed with 30 mL M9 solution into a slurry and 1.5 mL of the slurry was used as an additional inoculum for the next microcosm. The newly inoculated microcosms then entered the next 14‐day incubation Round, with the same sampling and genomic preparation protocols as in the first Round, and this was repeated for a total of four Rounds (i.e., 56 days in total).

### 2.2 Sequencing and Analysis of Raw Sequencing Data

The DNA samples collected from the experiment were subject to 16S meta‐barcoding for community composition determination. In detail, 16S rRNA v1–v2 regions in each sample was amplified by 30 cycles of PCR (primers 27F 5′‐AGAGTTTGATCCTGGCTCAG‐3′, 338R 5′‐TGCTGCCTCCCGTAGGAGT‐3′) according to the dual‐index library preparation strategy (Kozich et al. 2013). The PCR products were then sequenced on the Illumina MiSeq platform. Next, the R package DADA2 was used to clean the raw sequences according to (Callahan et al. 2016), and the Silva Project's version 138.1 prokaryotic SSU database was used for taxonomy assignment. The raw reads, the filtered reads and the outputs of the DADA2 pipeline are available at: https://doi.org/10.5281/zenodo.16760758.

The exact sequences, Amplicon Sequence Variants (ASVs), were used for further analyses. All analyses were performed on Python (Harris et al. 2020; Virtanen et al. 2020) unless otherwise specified. In total, 15,576 unique ASVs were detected. Each sample had on average 24,044 counts with a standard deviation of 27% (Figure S1).

### 2.3 Diversity Metrics

Alpha diversity metrics—Shannon's metric, Abundance‐based coverage estimator (ACE), Simpson's evenness, Faith's phylogenetic diversity (Faith's PD) and the number of ASVs in different relative abundance groups (Section A.1 and Figures S5–S8)—and beta diversity metrics—Jensen–Shannon divergence (JSD), Aitchison's metric and Unifrac metric (Section A.3 and Figures S9–S14)—were computed on the entire dataset, except for the phylogenetically informed metrics, Faith's PD and Unifrac distance, for which a reduced dataset was used (Sections A.1.4 and A.3.3).

### 2.4 Principal Component Analysis (PCA)

PCA was performed taking into account the compositional nature of the data by transforming them using the centered log ratio (clr) transform and correcting for zeros on the entire dataset as well as a reduced dataset by removing rare taxa (Section A.2 and Figure S19). The results of the PCA were similar in both cases (Figures S20 and 4). The contributions of taxa to displacement along Principal Component 1 (PC1) and Principal Component 2 (PC2) were calculated based on the loadings of eigenvectors of the PCA on the reduced dataset (Sections A.2.4 and A.2.5 and Figures 4E and S18).

### 2.5 Correlation Networks

The package SCNIC (Shaffer et al. 2023) was employed to infer positive and negative interaction networks and define modules of co‐varying ASVs (Section A.4) within each environment. Only ASVs that had a minimum relative abundance of 5% in any sample were used for this analysis.

### 2.6 Functional Analysis

The packages PICRUSt2 (Douglas et al. 2020) and FAPROTAX (Louca et al. 2016) (Sections A.5.1 and A.5.2) were used to infer functional potential from 16S rRNA gene data. The outputs of these packages were processed to obtain functions significantly differing across environments (Tables S1–S4) and across time‐points (Tables S5–S8). PCA was performed on the output of these packages (Figures 5, 6, S21–S23 and S30) to obtain functions significantly responsible for separating the environments. Further, Euclidean distances between the communities were computed based on the functional abundances. These distances were embedded in lower dimensions and the distances between technical replicates compared (Figures S24–S29 and S31–S33).

### 2.7 Statistical Analysis

The raw sequence data were processed as described in Section 2.2. The data were transformed for PCA using the centre log transform as described in Sections 2.4 and A.2. For other analyses, the counts data were converted to relative abundances. The two‐sample, two‐sided Kolmogorov–Smirnov test was used to quantify significance. The null hypothesis of this test is that the two samples are drawn from the same distribution. When a test's *p*‐value is low enough, the null hypothesis can be discarded. In such cases, where the null hypothesis could be discarded, the means of the two samples were compared to determine which group had the higher value. Scipy's (Virtanen et al. 2020) “ks_2samp” function was used with the default options. When testing multiple samples, the Bonferroni correction (Miller 1981) was applied to correct the limit of significant *p*‐value by dividing 0.05 by the number of tests. There are five replicates.

## 3 Results

Microbial communities sourced from compost were propagated in minimal media containing either glucose or cellulose as the sole carbon source. Communities (composed of five technical replicates) were incubated under static conditions for 14 days, then serially transferred to fresh media. This cycle was repeated for four Rounds (Rounds 1–4, Section 2). Community dynamics were tracked via daily 16S amplicon sequencing, and ASVs were used for analysis. For cellulose treatments, we distinguished between cells attached to the cellulose paper (CP) and those growing planktonically in the surrounding broth (CB).

### 3.1 Rapid Growth‐Death Dynamics in Glucose, Slow and Steady Dynamics in Cellulose

Community biomass, estimated using DNA concentration (Figure 1), revealed different dynamics across treatments. In CP communities, biomass gradually increased with minimal loss across Rounds, consistent with the recalcitrant nature of the carbon source, though with reduced yield in later Rounds (Figure 1A). In CB, biomass lagged behind CP but peaked late in Round 1, likely reflecting the liberation of soluble carbon by CP‐associated cellulolytic taxa (Figure 1B).

In glucose‐supported communities biomass was 10 to 100‐fold higher than in cellulose, but exhibited boom‐bust dynamics. In Rounds 1 and 2, biomass peaked by Day 5 before dropping sharply, consistent with rapid resource depletion and cell death. Fluctuations were more stable in Rounds 3 and 4 (Figure 1C).

**FIGURE 2:**
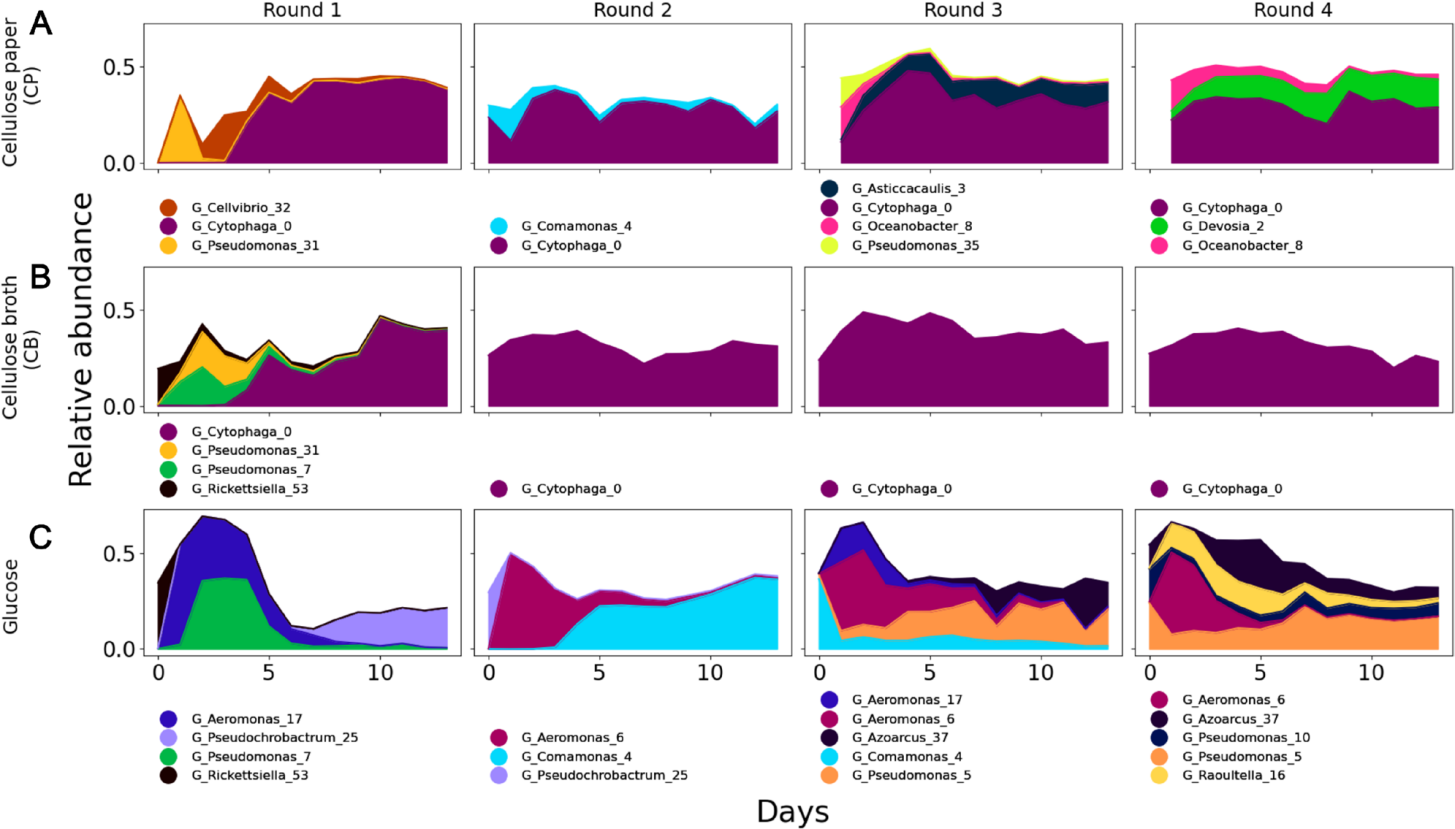
Dynamics of dominant taxa. Dominant taxa are defined as those taxa whose abundance was at least 15% in any community at any time point. Only a single replicate for each of the three conditions across the transfers are shown. The *x*‐axis represents days within each growth cycle, the *y*‐axis represents relative abundance. (A) Corresponds to communities on cellulose paper (CP), (B) to communities in cellulose broth (CB) and (C) to communities in glucose. Below each panel the identity of the ASVs are presented. G represents genus, the first taxonomic classification provided by SILVA. The numbers at the end represent unique identifiers for each ASV. Figures S2–S4 show the dynamics for the other four replicates.

To investigate these dynamics, we focused on high‐abundance ASVs (those with relative abundance >15%). All environments harboured transient colonizers, typically fast‐growing *Pseudomonas* (Figures 2 and S2–S4). In CP and CB, the taxon Cytophaga_0 dominated and remained stable over time (Figure 2A,B), mirroring biomass dynamics. In contrast, glucose environments showed rapid early expansion of dominant ASVs in each Round, followed by extinction and replacement (Figure 2C)—closely tracking biomass turnover (Figure 1C). As shown below, these differences in growth–death dynamics play a major role in determining the taxonomic and functional structure of communities in the two environments.

### 3.2 Similar Diversity Across Environments Despite Biomass Differences

**FIGURE 3:**
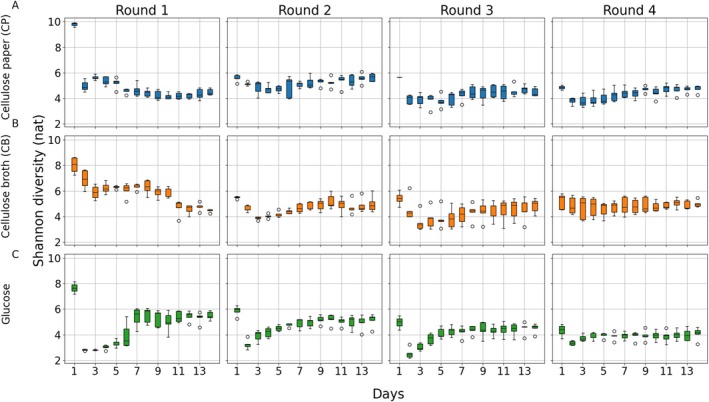
Shannon diversity dynamics across environments. Alpha diversity was quantified using the Shannon index (*y*‐axis) and tracked over 14 days of growth (*x*‐axis) across four Rounds of serial transfer (columns). Rows correspond to environments: cellulose paper, CP (A), cellulose broth, CB (B) and glucose (C). Each boxplot represents values across five replicate communities in each environment, where the boxes extend from the first to the third quartile, solid line represents the median, and the whiskers extend from the box to the farthest data point lying within 1.5 times the inter‐quartile range.

Given the differences in biomass across environments, we asked whether diversity metrics also varied. We computed Shannon diversity, richness, evenness and the number of ASVs across relative abundance ranges for all communities (Figures 3 and S5–S7) including all taxa. Overall, diversity metrics were broadly similar across treatments.

In glucose, particularly on Days 2–5 of Rounds 1, 2 and 4, there were more dominant strains (with >10% relative abundance) than in cellulose (Figure S5). However, at other time‐points, the number of ASVs in the 5%–10% and 1%–5% ranges was comparable across environments, accounting for the similar overall diversity across environments.

Shannon diversity was highest on Day 1 across all environments and Rounds, followed by a sharp decline (Figure 3). This pattern was mirrored by richness (Figure S6) and evenness (Figure S7). The elevated diversity on Day 1 in Round 1 reflects the high richness of the inocula, prior to acclimatization. In subsequent Rounds, transferred inocula contained ASVs that only began growing later in the Round. The early drop in diversity reflects the decline of these taxa, while the later recovery corresponds to their resurgence once appropriate substrates became available, indicating the importance of cross‐feeding.

In glucose, evenness increased after Day 5 (Figure S7, bottom row) because prior to Day 5, few strains dominated, reducing evenness (Figure S5, bottom row), while after Day 5, additional taxa rose in abundance, increasing evenness (Figures 2C and S4–S5 bottom row). In contrast, evenness remained relatively stable across days in CP and CB (Figure S7, top rows), consistent with the steady dynamics discussed earlier.

To further examine diversity trends, we compared the diversity metrics averaged across Days 13 and 14 of each Round, by which time communities had stabilized. By the end of Round 1, glucose communities were significantly richer, more even and more diverse than CB communities (Figure 3, Column 1; Figures S6 and S7; *p* < 0.008). By the end of Round 4 however, CB communities were richer and more diverse than those in glucose (Figure 3, Column 4; Figure S6; *p* < 0.05).

As expected, richness declined across Rounds in all environments due to strain loss during transfers (Figure S6). In glucose, the averaged final evenness did not change over Rounds, whereas in CB it increased significantly (*p* = 5.6e‐06; Figure S7). In Round 1, cellulose environments were dominated by one or two cellulolytic taxa (Figures 2A,B, S2, S3 and S5), leading to low evenness. In subsequent Rounds, cross‐feeders (ASVs unable to directly degrade cellulose) increased in abundance, raising evenness. In glucose, cross‐feeders appeared as early as Round 1 (Figures 2C and S4) due to rapid depletion of the supplied carbon, resulting in stable evenness across Rounds.

Taken together, these trends explain the observed patterns in Shannon diversity: in glucose, declining richness and stable evenness reduced diversity, while in cellulose, increasing evenness countered the loss in richness, resulting in higher overall diversity.

When phylogenetic information was incorporated into diversity calculations (Figure S8), glucose communities showed a marked increase in phylogenetic diversity by the end of each Round. Initially, closely related taxa dominated, but over time more distantly related taxa—likely cross‐feeders—rose in abundance. This pattern was less pronounced in the cellulose treatments, indicating that related taxa persist in the cellulose environment.

### 3.3 Communities on Cellulose and Glucose Remain Taxonomically Distinct, While Replicates Become Similar Over Time

**FIGURE 4:**
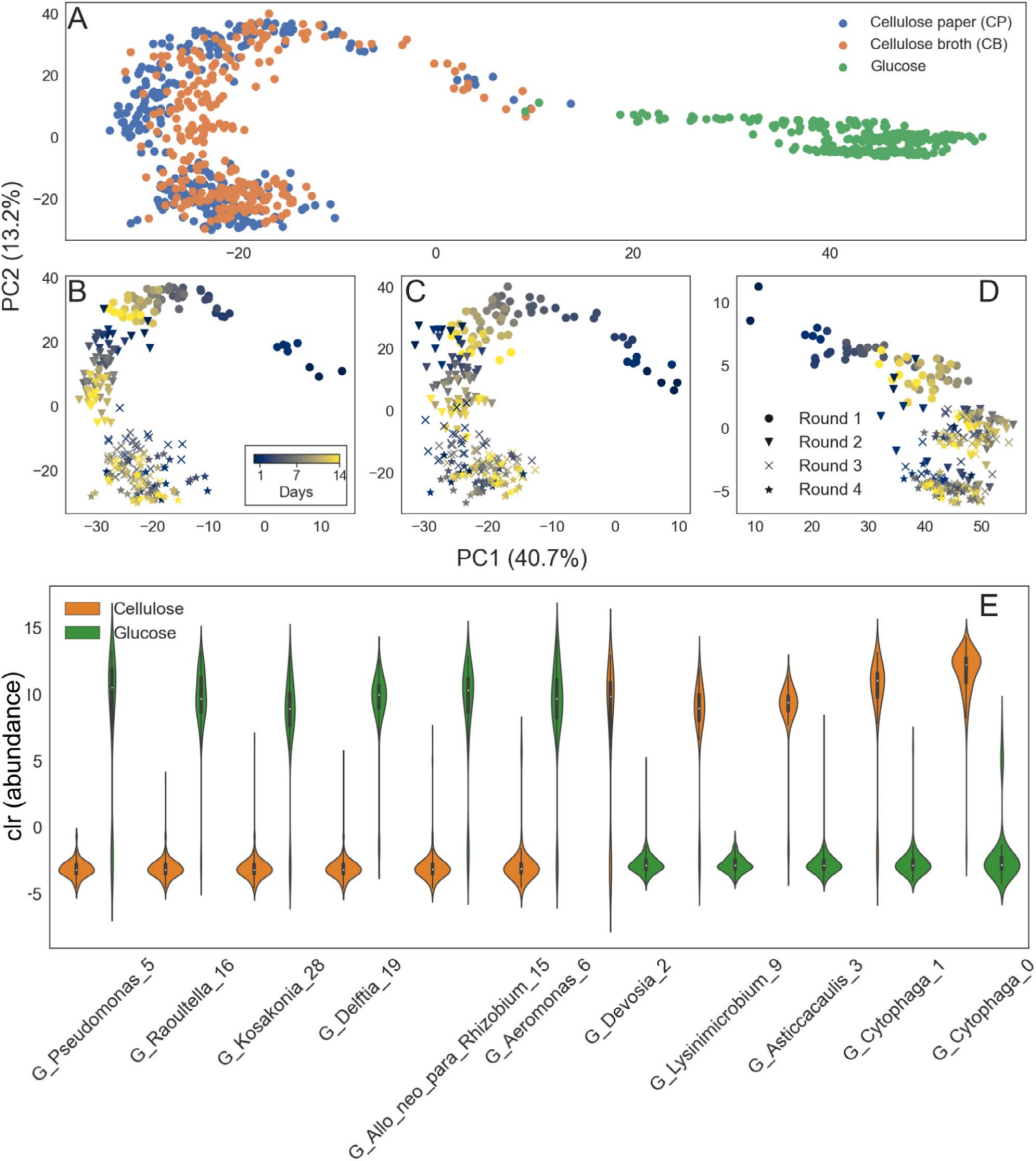
PCA on 16S sequence data. PCA was performed on the reduced dataset as described in Appendix A with five replicates for each condition. (A) The output of PCA plotted in two dimensions. Samples are colour coded by environment. (B–D) The same embedding as in (A), but separated by environment. (B) The samples on cellulose paper (CP), (C) the samples in cellulose broth (CB) and (D) the samples in glucose. Samples are colour coded by day, and markers represent the Rounds. (E) The log‐transformed abundances of taxa in cellulose and glucose environments that contribute most to separation of the two environments along PC1.

Having examined patterns in biomass and diversity, we next turned to community composition across environments and Rounds. The dynamics of dominant ASVs (Figures 2 and S2–S4) clearly show that glucose‐ and cellulose‐grown communities assembled distinct taxa, with CP and CB communities exhibiting similar compositions. This was further confirmed by PCA (Appendix A; Figure 4A–D). On Day 1 of Round 1, communities clustered together (dark blue circles around coordinate (10, 10) in Figure 4B–D), indicating initial similarity driven by the shared inoculum. However, as time progressed, glucose and cellulose communities diverged rapidly along the first principal component (PC1).

Successional dynamics differed across environments. In cellulose environments (CP and CB; Figure 4B,C), communities showed gradual change across Rounds 1 and 2. After Round 2, communities clustered more tightly, indicating that a steady state had been reached. In glucose, a similar pattern was observed during Round 1 (Figure 4D), but community structure stabilized by Round 2, indicating that the steady state was reached faster due to rapid growth‐death dynamics. These trends were corroborated by three beta‐diversity metrics independent of PCA: JSD, UniFrac distance and Aitchison distance (Figures S10, S12 and S14).

Analysis of distances between technical replicates (Figures S9, S11 and S13) revealed that within‐round replicate divergence decreased over time. Nonetheless, JSD and Aitchison distances showed increasing variance across Rounds, indicating that while communities generally became more similar, subgroups of replicates diverged from others. This is consistent with the emergence of dominant ASVs unique to individual replicates by Round 4 (Figures S2–S4). For example, on CP, Cytophaga_0 was a dominant member in all replicates at Round 1, but a minor component of Replicates 2, 3 and 5 at Round 4, with, in these replicates, Oceanobacter_8 having seemingly displaced Cytophaga_0 (Figures 2A and S3). In CB, Cytophaga_0 is the principal ASV across replicates in Rounds 1–3, but Nitrosomonas_24 dominates in Replicates 2 and 5 at Round 4 (Figures 2B and S2). In glucose, Pseudomonas_5 is a defining feature of three replicates, but is at low abundance in the other two at Round 4 (Figures 2C and S4). For UniFrac (Figure S13), which includes phylogenetic information, this increase in variance was not observed indicating that closely related strains replaced the previous strains, consistent with competition dynamics.

To assess the behaviour of low‐abundance taxa, we recalculated JSD using only ASVs with a maximum relative abundance of 1%. In contrast to full‐community analyses, these comparisons (Figure S15A–C) revealed that low‐abundance taxa were highly variable across replicates. Replicates clustered into two or three distinct groups with divergent compositions. Each group exhibited internal succession, but distances between groups increased over time (Figure S15G). Variance in glucose was higher than in cellulose as expected (Section 4). Therefore, while dominant taxa were largely shared across replicates, low‐abundance taxa varied substantially.

### 3.4 Specialists Favoured in Cellulose Environments While Generalists Dominate in Glucose Environments

We next examined the taxa that contributed to differences between communities grown on cellulose versus glucose. In cellulose environments (Figures 2A,B, S2 and S3), Round 1 was characterized by transient growth of Pseudomonas_31, followed by dominance of the cellulose‐degrading strain Cytophaga_0 (Zhu and McBride 2014). On CP, Cellvibrio_32 also became abundant (Figure 2A), an unsurprising result because members of the genus *Cellvibrio* are known to produce glucosidase, a key enzyme in cellulose degradation (Mergaert et al. 2003). In subsequent Rounds, Cytophaga_0 remained dominant. Additional taxa also rose in CP communities (Figure S3), including Asticcacaulis_3 and Lysinimicrobium_9, both producers of α‐ and β‐glucosidases (Hamada et al. 2017; Zhou et al. 2020). Strains from the genera *Comamonas* and *Devosia*, known for metabolizing aromatic hydrocarbons (Wilkes et al. 2023; Talwar et al. 2020), also became abundant, as did marine‐associated *Oceanobacter* species (Satomi et al. 2002; Bowditch et al. 1984), which consume long‐chain and branched alkanes (Teramoto et al. 2009). In some replicates, *Nitrosomonas* strains appeared in later Rounds (Figures S2 and S3); these ammonia‐oxidizing bacteria (Koops et al. 1991), which fix carbon via CO_2_, may have competed with cellulose degraders for nitrogen. Overall, taxa enriched in cellulose were either direct cellulose degraders, users of alternative energy sources like ammonia, or specialists in metabolizing specific intermediates.

The dynamics of closely related ASVs offer further insight. While Cytophaga_0 was dominant across replicates and Rounds, two closely related ASVs (differing by one nucleotide) co‐existed in both CP and CB (Figure S16A, D). This is unexpected given that closely related taxa are likely to compete for the same niche. In contrast, the dominant *Nitrosomonas* strains emerging by Round 4 had no closely related variants in the community (Figure S16B,C,E,F and Section 4).

In glucose, early dominance in Round 1 came from *Aeromonas* and *Pseudomonas* (Figures 2C and S4). In Round 2, Aeromonas_6 became dominant and persisted through serial dilutions. In Rounds 3 and 4, additional strains of *Pseudomonas* and *Aeromonas* rose in abundance. Nitrogen‐fixing taxa such as Azoarcus_37 (Saini et al. 2023; Porter and Young 2014) and Allorhizobium‐Neorhizobium‐Pararhizobium‐Rhizobium_15 became prominent in the final Rounds. Thus, glucose environments favoured generalist taxa like *Pseudomonas* (Karishma et al. 2015), *Aeromonas* (Prediger et al. 2016) and nitrogen fixers. Nitrogen fixers are expected in the glucose environment because the rapid consumption of glucose also implies the rapid depletion of the supplied ammonium, and new ways of bringing nitrogen into the environment or alternate energy production pathways such as nitrogen fixation are likely to be favoured.

Fine‐scale variation among closely related ASVs further highlights these dynamics. Pseudomonas_22, which differs by one nucleotide from the initially dominant Pseudomonas_7, persisted longer into Round 2 before declining (Figure S17A). Pseudomonas_5 became dominant by Rounds 3 and 4, alongside Pseudomonas_10, which differs from it by one nucleotide (Figure S17B). Aeromonas_6, dominant in Rounds 2–4, is nearly identical to Aeromonas_17, which was dominant in Round 1 but rare thereafter (Figure S17C). In contrast, Azoarcus_37 (Figure S17D), which dominated in three replicates, had no close sequence variant (Section 4).

PCA supports these taxonomic distinctions. PC1 separates cellulose and glucose communities (Figure 4A). Loadings along the first eigenvector (Appendix A) identify the taxa most responsible for this separation. Log‐transformed abundances of these taxa (Figure 4E) confirm that cellulose is characterized by degraders like *Cytophaga*, *Lysinimicrobium*, *Asticcacaulis* and metabolizers of aromatic compounds like *Devosia*, while glucose is enriched for generalists such as *Pseudomonas*, *Aeromonas* and nitrogen‐fixing *Allorhizobium–Neorhizobium–Pararhizobium–Rhizobium*.

From Figure 4A–D, we see that while PC1 separated the environments, the communities are displaced downwards along PC2 with time. Using the eigenvector loadings along PC2 (Appendix A), we identified the taxa that contributed most to this temporal displacement. In cellulose environments (Figure S18, top panels), generalists such as *Pseudomonas* and *Flavobacterium* (Spormann 2023a) declined sharply from Rounds 2 to 4, contributing to PC2 displacement. At the same time, specialist taxa such as the cellulose‐degrading Chitinophagaceae_58 (Bailey et al. 2013; Rosenberg 2014; Funnicelli et al. 2021) and ammonia‐oxidizing Nitrosomonas_14 increased in abundance by Round 4, indicating temporal selection for specialists in cellulose. Although some specialist taxa such as Cellvibrionaceae_34, Cellvibrio_80 and Devosia_64 decreased in abundance by Round 4, related strains from the same genera or families remained, suggesting competition among closely related specialist lineages. In glucose communities (Figure S18, bottom panel), some generalists declined over time (e.g., Pseudomonas_22, Pseudomonas_7) or remained at low abundance (e.g., Pseudomonas_27, Flavobacterium_36), while others such as Pseudomonas_12 increased in abundance. The nitrogen‐fixing strain Azoarcus_37 also contributed to displacement along PC2, rising in abundance in some replicates by Round 4 (Figure S4). These results indicate selection for generalists and nitrogen fixers in glucose over time.

### 3.5 Interactions Between Taxa Independent of Relative Abundance and Environment

We next asked whether taxa in the communities exhibited signs of interaction. To explore this, we used correlations in abundance as proxies for potential interactions (Appendix A). Files S1 and S2 display the network modules for positive and negative interactions, respectively. In all environments, positive interactions between ASVs outnumbered negative ones, suggesting enhanced cross‐feeding.

Modules of co‐varying taxa were found to contain overlapping ASVs across environments, and the average strength of interactions within these modules was consistent across conditions. This suggests that interaction patterns were largely independent of environmental context, in particular, the main source of carbon.

We also tested whether interaction patterns were linked to taxon abundance. There was no correlation between the average relative abundance of ASVs and their number of interactions in any environment except glucose (Pearson *r* ∼0.6, *p*‐value $∼9\times 10^{-6}$). Likewise, average interaction strength was not correlated with abundance in any environment except for a weak correlation in CB (Pearson *r* ∼0.4, *p*‐value ∼0.02). These findings indicate that observed interactions were generally independent of ASV abundance.

### 3.6 Distinct Functions Are Evident in Different Environments

To explore functional differences between communities grown on glucose and cellulose, we used PICRUSt2 (Douglas et al. 2020) and FAPROTAX (Louca et al. 2016) to infer functional potential from 16S rRNA gene data. PICRUSt2 provided profiles of enzyme composition, Kegg Orthologs (KOs) and metabolic pathways, while FAPROTAX predicted broader functional capabilities. These data were analysed by direct comparison between treatments and via PCA (Appendix A).

**FIGURE 5:**
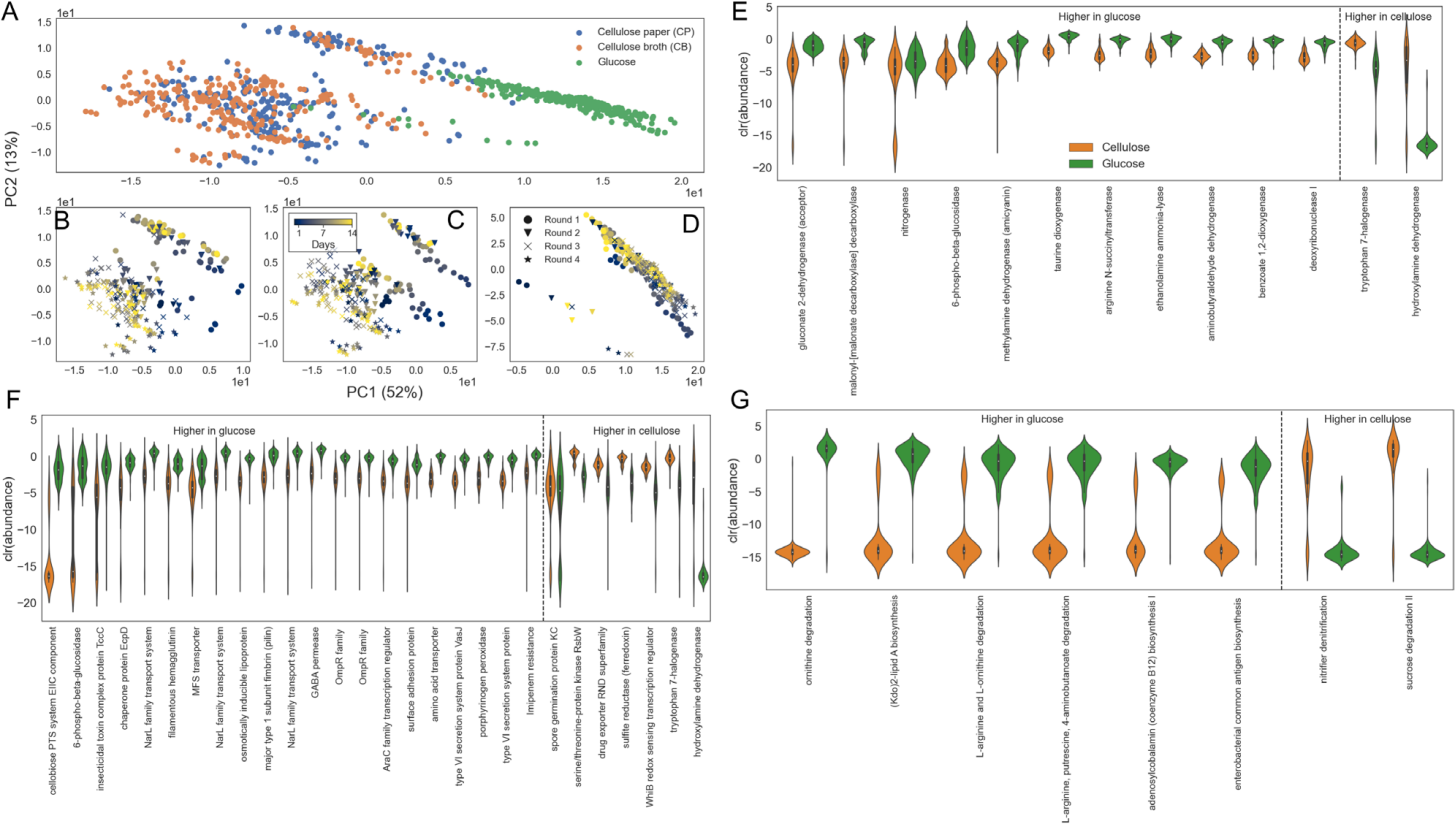
PCA on PICRUSt2 output. PCA was performed on the output of PICRUSt2 as described in Appendix A on all five replicates in all three conditions. (A) Output of PCA on the abundance of EC numbers, embedded in two dimensions. The samples are colour coded by their environments. (B–D) The same embedding as in (A) separated by the environment. (B) The samples on cellulose paper (CP), (C) the samples in cellulose broth (CB) and (D) the samples in glucose. The samples are colour coded by day, and the markers represent Rounds of transfer. (E) The log‐transformed EC number abundances in the cellulose and glucose environments contributing most to separation of the two environments is shown along PC1. (F) The log‐transformed Kegg Ortholog abundances in the cellulose and glucose environments contributing the most to separation of the two environments is shown along PC1. (G) The log‐transformed pathway abundances in the cellulose and glucose environments contributing the most to the separation of the two environments along PC1.

**FIGURE 6:**
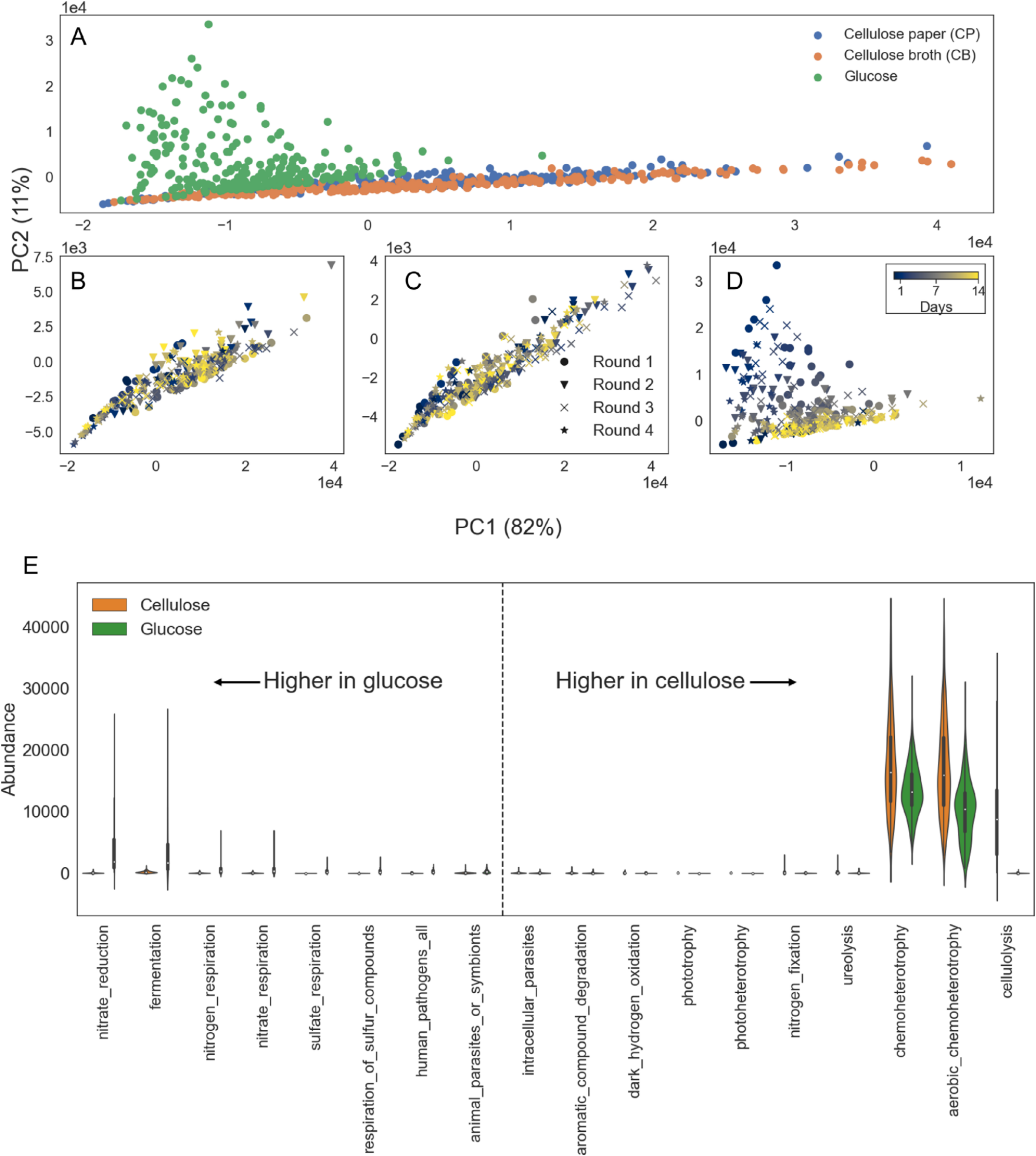
PCA on FAPROTAX output. PCA was performed on the output of FAPROTAX as described in Appendix A on all five replicates in all three environments. (A) Output of the PCA, embedded in two dimensions. The samples are colour coded by environment. (B, D) The same embedding as in (A) separated by the environment. (B) The samples on cellulose paper (CP), (C) the samples in cellulose broth (CB) and (D) the samples in glucose. The samples are colour coded by the days, and the markers represent the Rounds. (E) The functional number abundances in the cellulose and glucose environments contributing the most to the separation of the two environments along PC2.

The analyses revealed clear functional distinctions between communities in the two environments (Figures 5 and 6A–D and Tables S1–S4). Further, replicate communities in CP and CB remained functionally similar over time, while replicates in the glucose‐supported environment displayed temporal variation in each Round (Figures S25, S27, S29 and S31), reflecting the dynamics observed in the taxonomy. Across Rounds, functional similarity between the replicates did not vary (Figures S25, S27, S29 and S31). In addition to the major functional differences described below, distinct sets of transporters, regulatory elements and stress response systems were enriched in each environment.

#### 3.6.1 Cellulolysis, Aerobic Respiration, Cross‐Feeding and Alternate Energy Production Are Prevalent in Cellulose

As expected from taxonomy, communities grown on cellulose were enriched for genes involved in cellulose degradation (Table S4 and Figure 6E) as indicated by both PICRUSt2 and FAPROTAX. Aerobic respiration was more prominent in cellulose environments (Tables S1–S4 and Figure 5F). Slower bacterial growth on cellulose (as noted previously from biomass and composition analyses, Figures 1A,B and 2A,B) reduces oxygen consumption relative to glucose, resulting in more sustained aerobic conditions (Section 4), consistent with both FAPROTAX and PICRUSt2 predictions.

Enrichment of pathways associated with metabolite salvage (Tables S1–S4 and Figure 6E) and ABC transporter systems (Table S2) indicates transport and cross‐feeding of secondary metabolites in cellulose‐grown communities. Pathways for energy production independent of cellulose, including ammonia oxidation (nitrifier denitrification, Figure 5G, observed in strains belonging to genus *Nitrosomonas* in Figures S2 and S3) and sulphate assimilation (Figure 5F), were also enriched, consistent with the recalcitrant nature of cellulose.

Other dominant functional categories included branched‐chain amino acid metabolism, cell wall synthesis and DNA replication (Tables S1–S3), suggesting prioritization of growth‐related processes over energy storage (Section 4).

#### 3.6.2 C, N Cycles, Salvage and Biofilm Formation Are Enriched in Glucose Communities

In the glucose environment, pathways related to the TCA cycle (Tables S1 and S3) were enriched—a result consistent with glucose abundance and with the dominance of generalists as noted in the analyses of community composition.

High community turnover in glucose was reflected in enhanced salvaging and cross‐feeding pathways, including transport and cleavage of amino acids (Tables S1–S3 and Figure 5F), consistent with patterns observed in biomass and taxonomy. Enrichment of lipid synthesis pathways (Tables S1–S3 and Figure 5G) suggests both scavenging from lysed cells (Tables S1–S3 and Figure 5E) and lipid storage under high‐carbon conditions (Section 4). Increased cell lysis is further supported by enrichment of the osmolarity‐inducible lipoprotein and MFS transporters (Figure 5F), likely reflecting adaptation to solute release following frequent lysis.

Sensory proteins were also enriched (Table S2), suggesting increased responsiveness to diverse metabolites present in the environment.

Functions related to nitrogen cycling were also enriched (Figures 5E–G and 6E and Tables S1–S4), as indicated by both PICRUSt2 and FAPROTAX. This is consistent with rapid bacterial growth in glucose depleting available nitrogen, necessitating alternative nitrogen acquisition pathways (Section 4). These functions are also consistent with the observation of nitrogen fixing bacteria in glucose environments (Figure S4). These inferred functions were confirmed by metagenomic analyses (Figures S34–S37).

Biofilm‐related functions were a major feature of glucose communities. Components of the Type VI secretion system (Table S1 and Figure 5F) (Coulthurst 2013), the major subunit of Type 1 fimbriae (pilin), surface adhesion proteins and filamentous hemagglutinin were all enriched (Figure 5F). These findings are consistent with direct observations of biofilm formation in glucose cultures.

#### 3.6.3 Distinct Functions Activated in the Initial and Final Days in Glucose

Figures 5D and 6D show that in the glucose environment, communities at equivalent time‐points across different Rounds clustered closely. This was true for the other outputs of PICRUSt2 (Figures S22 and S23) and further confirmed independently of PCA by embedding a distance metric (Appendix A and Figures S24, S26, S28, S32 and S33). To understand those functions providing temporal separation of communities in glucose, we compared the functional profiles of communities from Days 3 and 4 with those from Days 13 and 14, across all Rounds (Appendix A). Dominant functions at each time‐point are presented in Tables S5–S8.

As expected, enzymes associated with sugar catabolism and TCA cycle intermediates were enriched during the early phase (Tables S5 and S6), when glucose is available. Interestingly, both anaerobic and aerobic respiration pathways (Tables S5 and S6) were detected early. By contrast, aerobic respiration dominated at later time points (Tables S5, S7 and S8), as indicated by both PICRUSt2 and FAPROTAX. This shift is consistent with biomass dynamics: rapid, glucose‐fuelled growth early in propagation drives high respiratory demand and steep oxygen gradients, generating localized anaerobic microenvironments, whereas declining growth and biomass turnover at later stages reduce oxygen consumption, allowing re‐equilibration and favouring aerobic respiration. These results are consistent with taxonomic analyses: strains of *Aeromonas* and *Comamonas*, which are dominant at the beginning of growth (Figures 2C and S4) in glucose, can be anaerobic (Percival and Williams 2014; Li et al. 2022).

Salvage pathways were already active by Days 3 and 4 (Tables S5 and S7), confirming substantial early cell turnover. By the final Days, communities showed elevated activity in ABC transporters, fatty acid synthesis and lipid metabolism, consistent with storage and scavenging (Tables S5–S7; Section 4).

An increase in serine transporter abundance during the early phase is noteworthy (Table S6). This transporter is known to prevent cell lysis under glucose‐depleted conditions (Kriner and Subramaniam 2020), potentially explaining how initially dominant ASVs, such as Aeromonas_6, persist across Rounds.

Nitrogen metabolism also shifted over time. While nitrate reduction was enriched in the early Days, a broader suite of nitrogen‐related functions—including ammonia assimilation, nitrate and nitrogen respiration and ureolysis—was upregulated in later stages (Table S8), suggesting intensifying nitrogen competition. This is consistent with the appearance of nitrogen fixing strains towards the ends of the Rounds (Figure 2C).

In the final Days, enrichment of receptors (Tables S5 and S6) indicated increased sensing of and response to available metabolites, consistent with enhanced cross‐feeding. Sulphate and sulphur respiration also increased (Table S8), further supporting the view of intensified resource competition over time.

## 4 Discussion

A central finding of this study is that high microbial diversity can be maintained on both recalcitrant and labile carbon sources, despite large differences in community composition, function and biomass. From a classical ecological perspective, this outcome is surprising. Foundational theory—supported by early microbial evolution experiments in well‐mixed chemostats—predicts that single limiting resources should support only a few dominant taxa due to competitive exclusion (e.g., (Helling et al. 1987; Levin 1972; Stewart and Levin 1973; MacArthur and Levins 1964; Tilman 1982)). The “paradox of the plankton” and other works (Hutchinson 1961; Rainey et al. 2000; Kerr et al. 2002) articulated this puzzle more broadly: how can such high diversity persist in seemingly uniform environments? More recent work, particularly in microbial systems, has begun to reconcile this paradox. Studies have shown (Goldford et al. 2018; Prabhakara and Kuehn 2023; Astacio et al. 2021; Roman and Wagner 2018; Bello et al. 2021; Xu et al. 2018; Ortega‐Retuerta et al. 2021; Noriega‐Ortega et al. 2019; Bloxham et al. 2024; Pacciani‐Mori et al. 2020; Zhu et al. 2024; Serván et al. 2018) that metabolic cross‐feeding, temporal niche generation and eco‐evolutionary feedbacks can sustain complex communities even on single carbon sources. Our results contribute to this growing body of evidence by demonstrating that distinct but comparably diverse microbial communities can assemble and persist on either glucose or cellulose—substrates that differ dramatically in structure and accessibility.

Glucose and cellulose gave rise to taxonomically and functionally distinct communities, consistent with expectations based on carbon chemistry. Glucose‐supported communities exhibited rapid growth, high biomass and early dominance by fast‐growing generalists. High biomass was sustained, despite expectations that supplied glucose would be depleted within 4–5 days, by continued access to diverse nutrients released from dead cells as a consequence of high turnover. Correspondingly, anabolic pathways were enhanced in these communities, a result expected from previous analyses of proteome allocation (Spormann 2023b; Hui et al. 2015). Cellulose‐supported communities, by contrast, assembled slowly, maintained lower biomass and were enriched for taxa known to specialize on complex polysaccharides. These differences likely reflect underlying metabolic trade‐offs. As noted by (Shan et al. 2024), microbial investment in glycosidic enzymes—essential for cellulose degradation—is anti‐correlated with investment in cell replication machinery. Such a trade‐off could explain the lower overall productivity in cellulose environments. However, despite slower growth and lower resource availability, cellulose‐grown communities ultimately achieved similar levels of taxonomic diversity. In fact, the cellulose‐supported communities eventually became richer than the glucose‐supported communities, consistent with expectations from the number of secondary metabolites produced by the two substrates (Bello et al. 2021). This suggests that recalcitrant substrates, while energetically costly to exploit, promote sustained diversity by enabling niche partitioning and slowing competitive exclusion.

A second explanation of the difference lies in the emergence of cross‐feeding and metabolic interdependence. Functional inference revealed extensive but distinct metabolite exchange in both environments, including enrichment of chemotaxis and transporter systems, particularly in glucose‐grown communities. Both environments also showed strong signatures of resource salvage from dead cells; necromass has recently been shown to play an important role in maintaining diversity in complex communities, with its quantity and composition shaping community structure (Hao et al. 2026). Taxon co‐occurrence patterns further indicate that low‐abundance taxa are highly connected within communities and likely depend on metabolic by‐products produced by dominant taxa irrespective of the environment. This is consistent with previous findings that even single‐resource environments can give rise to metabolically structured communities through niche construction and resource diversification (Goldford et al. 2018; Bello et al. 2021). The persistence of cross‐feeders at low abundance, the prevalence of positive interactions and the apparent independence of interaction number and strength from taxon abundance together suggest that these taxa play a key role in sustaining diversity, with ecosystem function emerging from distributed, cooperative interactions.

Third, nutrient stoichiometry and dynamic resource limitation likely shaped community composition in distinct ways. Although the growth media had similar C:N:P ratios in both conditions, the rate and mode of carbon consumption differed. In glucose, rapid uptake led to nitrogen limitation, as indicated by enrichment of nitrogen transporters and nitrogen‐cycling functions. This caused strong competition, reflected in the survival of only distantly related ASVs performing nitrogen cycling functions. In cellulose, carbon was released more slowly, favouring energy‐efficient respiratory strategies such as oxidative phosphorylation, reducing the intensity of nitrogen competition and promoting alternative energy production pathways. Apart from carbon, nitrogen and phosphorus, the availability of oxygen was also different—rapid growth in the glucose environment likely created anaerobic pockets, while the cellulose‐supported environment remained aerobic. These contrasting metabolic regimes—driven by the kinetics of substrate availability—help explain the functional divergence between communities and support the idea that nutrient imbalance can generate indirect niche dimensions even in nominally simple environments.

Despite pronounced taxonomic turnover, functional profiles remained relatively stable within each environment. Communities grown on glucose showed similar functional signatures across early time‐points in different experimental Rounds, while cellulose communities maintained functional consistency throughout. This decoupling between taxonomy and function indicates strong functional redundancy—a common feature of complex microbial communities (Louca et al. 2018; Li et al. 2021, 2024). In our case, it suggests that while species composition may be dynamic, the ecological roles required to process each carbon source are consistently fulfilled. Such redundancy may buffer ecosystem function against stochastic demographic fluctuations or taxon loss.

Our findings contrast with previous reports of highly reproducible assembly dynamics in glucose‐supplemented communities inoculated from soil, such as those described by Goldford et al. (Goldford et al. 2018). In that study, just 12 batch transfers (∼84 generations) in shaken culture led to the rapid emergence of simple and convergent communities dominated by a few metabolic generalists. That outcome was interpreted as evidence of universal metabolic constraints driving deterministic assembly. However, the reproducibility observed in such systems likely reflects strong ecological filtering arising from both inoculum preconditioning and the propagation regime, which favours fast‐growing fermentative and respiratory taxa.

In our experiments, despite using glucose as the sole carbon source and observing initial dominance by fast‐growing *Pseudomonas*, community dynamics were less predictable: biomass levels fluctuated sharply, dominant taxa turned over within Rounds and diversity metrics—especially evenness—varied significantly across time and replicates. Cross‐feeders emerged early and maintained diversity, but selection did not drive strong convergence. These findings suggest that deterministic simplicity is not an inevitable outcome of microbial community assembly on labile carbon sources. Instead, it emerges under particular combinations of environmental structure, propagation regime and selection intensity. Our results point to a more contingent and historically structured assembly process, even under nominally simple conditions.

Patterns of divergence across replicate communities reveal an interplay between deterministic and stochastic forces—the latter likely influential on dynamics of low abundance taxa. While dominant taxa converged within each environment—suggesting selection under shared conditions—lower‐abundance ASVs diverged across replicates, especially in glucose. This divergence likely reflects increasing environmental complexity over time, driven by higher cell turnover, by‐product accumulation and niche diversification. Prior work has shown that microbial communities can diverge more in complex environments (Silverstein et al. 2024), supporting our hypothesis that more diverse by‐products in glucose compared to cellulose cause stronger divergence in the low abundance taxa that depend on these by‐products.

Spatial structure further modulated community composition. In the cellulose environment, paper‐associated communities differed from those in the surrounding broth. Taxa colonizing the cellulose matrix were more abundant and interacted with a greater number of partners, consistent with the hypothesis that spatial partitioning can promote coexistence by reducing direct competition. While differences in diversity between compartments were modest, the presence of physical structure appears to create microenvironments that facilitate co‐assembly and resource partitioning.

Although ASVs that differ by only a single nucleotide are clearly closely related, they can nevertheless differ substantially in accessory gene content. Such ASVs frequently co‐occur in our communities, but it is often unclear whether they represent distinct species within the same genus or different strains of the same species. While the coexistence of multiple species within a genus is readily explained, the stable coexistence of closely related strains poses a greater challenge. One way to distinguish between these possibilities is to examine genome databases. For instance, the Genome Taxonomy Database contains only six strains assigned to the genus *Cytophaga*, but 8751 strains for *Pseudomonas*. Without further information, it remains difficult to determine whether our ASVs reflect intra‐ or interspecific diversity. However, this distinction is important—particularly in the context of competitive interactions (Rossum et al. 2020)—and future work involving strain isolation and genome sequencing will be required to clarify these relationships.

Together, these findings reveal that microbial diversity can persist under far simpler resource conditions than previously assumed. Functional redundancy, emergent metabolic interactions, dynamic nutrient limitation and spatial structure all contribute to this outcome. Rather than acting in isolation, these processes interact to shape distinct yet diverse communities across contrasting environments. Our results underscore the importance of considering not only the identity of available resources but also the ecological dynamics they generate—including feedbacks through by‐product formation, growth kinetics and interspecies interactions. Ongoing work is aimed at disentangling these mechanisms more precisely through isolate‐based reconstructions, genome‐resolved metagenomics and experimental manipulation of interaction networks. Of particular importance is understanding the relationship between diversity maintained in these experimental systems and diversity of types present in the source inoculum.

## 5 Limitations of the Study

This study has two primary limitations. First, while the initial inocula derived from compost contained both prokaryotic and eukaryotic organisms, our sequencing data capture only the prokaryotic component. As a result, we may have overlooked potentially important eukaryotic contributions to community dynamics and function. Second, all functional inferences were based on 16S rRNA gene sequences, using predictive tools that rely on reference genomes. This approach assumes that taxa with similar 16S sequences share similar functional repertoires, an assumption that may not hold for diverse environmental strains. Consequently, the inferred functional profiles should be interpreted with caution.

## Author Contributions

**Kaumudi H. Prabhakara:** visualization, formal analysis, writing – original draft, writing – review and editing. **Yansong Zhao:** conceptualization, investigation, methodology, writing – review and editing. **Kristian Ullrich:** software, formal analysis. **Andrew D. Farr:** writing – review and editing. **Paul B. Rainey:** conceptualization, methodology, resources, writing – review and editing, supervision.

## Funding

This work was supported by Max‐Planck‐Gesellschaft.

## Conflicts of Interest

The authors declare no conflicts of interest.

## Supporting information


**Figure S1:** Number of reads per sample. The number of counts plotted on the *y*‐axis for each sample sequenced, plotted on the *x*‐axis.
**Figure S2:** Dynamics of dominant taxa in cellulose broth communities. The dynamics of the dominant taxa in cellulose broth as defined in Methods for other four replicates are shown here. The *y*‐axis shows the relative abundance and the *x*‐axis shows the days of sampling. The columns are the Rounds of transfer. Below each column the legends show the identity of the ASVs in terms of the lowest phylogenetic classification provided by SILVA. G denotes Genus, F denotes Family. The numbers at the end are unique identifiers for each ASV detected.
**Figure S3:** Dynamics of dominant taxa on cellulose paper communities. The dynamics of the dominant taxa on cellulose paper as defined in Methods for other four replicates are shown here. The *y*‐axis shows the relative abundance and the *x*‐axis shows the days of sampling. The columns are the rounds of transfer. Below each column the legends show the identity of the ASVs in terms of the lowest phylogenetic classification provided by SILVA. G denotes Genus, F denotes Family. The numbers at the end are unique identifiers for each ASV detected.
**Figure S4:** Dynamics of dominant taxa in glucose communities. The dynamics of the dominant taxa in glucose as defined in Methods for other four replicates are shown here. The *y*‐axis shows the relative abundance and the *x*‐axis shows the days of sampling. The columns are the rounds of transfer. Below each column the legends show the identity of the ASVs in terms of the lowest phylogenetic classification provided by SILVA. G denotes Genus. The numbers at the end are unique identifiers for each ASV detected.
**Figure S5:** Number of taxa within given relative abundance ranges. The number of taxa in relative abundance ranges above 10%, denoted by circles, between 5% and 10% denoted by triangles, and between 1% and 5% denoted by asterisks, are plotted by averaging the abundances across replicates. The top row shows these data for communities in cellulose broth, the middle row for communities on paper, and the bottom row for communities on glucose.
**Figure S6:** ACE diversity metric. The alpha diversity metric, abundance‐based coverage estimate, which represents the richness, is shown on the *y*‐axis. The *x*‐axis shows the days of sampling. Each column shows the rounds of transfer. The top row shows these data for communities in cellulose broth, the middle row for communities on paper, and the bottom row for communities on glucose.
**Figure S7:** Simpsonʼs evenness measure: The alpha diversity metric, Simpsonʼs evenness, which represents the evenness, is shown on the *y*‐axis. The *x*‐axis shows the days of sampling. Each column shows the rounds of transfer. The top row shows these data for communities in cellulose broth, the middle row for communities on paper, and the bottom row for communities on glucose.
**Figure S8:** Faithʼs phylogenetic diversity. The alpha diversity metric, Faithʼs PD, which takes into account the phylogenetic information, is shown on the *y*‐axis. The *x*‐axis shows the days of sampling. Each column shows the rounds of transfer. The top row shows these data for communities in cellulose broth, the middle row for communities on paper, and the bottom row for communities on glucose.
**Figure S9:** Jensen–Shannon divergence (JSD). The beta diversity metric, JSD, is plotted on the *y*‐axis for the replicates. The *x*‐axis shows the days of sampling. Each column shows the rounds of transfer. The top row shows these data for communities in cellulose broth, the middle row for communities on paper, and the bottom row for communities on glucose. Each boxplot represents values across five replicate communities in each environment, where the boxes extend from the first to the third quartile, solid line represents the median, and the whiskers extend from the box to the farthest data point lying within 1.5 times the inter‐quartile range.
**Figure S10:** Embedding of Jensen–Shannon divergence metric. The JSD between all samples were embedded in two dimensions for each environment: (A) for cellulose broth (B) for paper and (C) for glucose. The bottom panels show the stress of the MDS embedding in each environment.
**Figure S11:** Aitchisonʼs distance metric. The beta diversity metric, Aitchisonʼs metric, which takes into account the compositional nature of the data is plotted on the *y*‐axis for the replicates. The *x*‐axis shows the days of sampling. Each column shows the rounds of transfer. The top row shows these data for communities in cellulose broth, the middle row for communities on paper, and the bottom row for communities on glucose. Each boxplot represents values across five replicate communities in each environment, where the boxes extend from the first to the third quartile, solid line represents the median, and the whiskers extend from the box to the farthest data point lying within 1.5 times the inter‐quartile range.
**Figure S12:** Embedding Aitchisonʼs distances. The Aitchisonʼs distance between all samples were embedded in two dimensions for each environment: (A) for cellulose broth (B) for paper and (C) for glucose. The bottom panels show the stress of the MDS embedding in each environment.
**Figure S13:** Unifrac distances across days. The beta diversity metric, Unifrac, which takes into account the phylogenetic information of the samples is plotted on the *y*‐axis for the replicates. The *x*‐axis shows the days of sampling. Each column shows the rounds of transfer. The top row shows these data for communities in cellulose broth, the middle row for communities on paper, and the bottom row for communities on glucose. Each boxplot represents values across five replicate communities in each environment, where the boxes extend from the first to the third quartile, solid line represents the median, and the whiskers extend from the box to the farthest data point lying within 1.5 times the inter‐quartile range.
**Figure S14:** Embedding Unifrac distances. The Unifrac distance between all samples were embedded in two dimensions for each environment: (A) for cellulose broth (B) for paper and (C) for glucose. The bottom panels show the stress of the MDS embedding in each environment.
**Figure S15:** JSD for only low abundance taxa. The JSD was computed for the low abundance ASVs as described in Methods. The pairwise distances were embedded in two dimensions for each environment: (A) for cellulose broth (B) for paper and (C) for glucose. (D–F) The stress of the MDS embedding in each environment. (G) The distances between replicates; the top row for cellulose broth, the middle row for cellulose paper and the bottom row for glucose broth. Each boxplot represents values across five replicate communities in each environment, where the boxes extend from the first to the third quartile, solid line represents the median, and the whiskers extend from the box to the farthest data point lying within 1.5 times the inter‐quartile range.
**Figure S16:** ASVs with 1 nucleotide differences in the cellulose environment. The relative abundance of the dominant ASV is plotted in black across all 14 days of all four rounds. In other colours, relative abundance of ASVs that are one nucleotide different from the dominant ASV are plotted (A–C) in the cellulose broth, (D–F) on paper. The different curves represent abundances in different replicates.
**Figure S17:** ASVs with 1 nucleotide differences in the glucose environment. The relative abundance of the dominant ASV is plotted in black across all 14 days of all four rounds. In other colours, relative abundance of ASVs that are 1 nucleotide different from the dominant ASV are plotted for (A) *Psedumonas* 7, (B) *Pseudmonas* 5, (C) *Aeromonas* 6 and (D) Azoarcus 37. The different curves represent the abundances in different replicates.
**Figure S18:** ASVs causing displacement of communities along PC2 between Round 2 and Round 4. The contribution of the ASVs to the displacement of communities between Rounds 2 and 4 were calculated the top contributors were chosen, as described in Methods. The log ‐transformed relative abundances in Rounds 2 and 4 for these top contributors are plotted for the cellulose broth environment in the top panel, the cellulose paper environment for the middle panel and the glucose broth environment for the bottom panel. The violin plots represent the distributions across the 5 technical replicates.
**Figure S19:** Thresholding and eigenvalues of thresholded data. For the PCA on 16S sequence data, the thresholding was performed as in Methods. (A) The logarithm of the frequency of maximum counts an ASV has across all sample. The dashed line shows the cutoff for thresholding. (B) The eigenvalue distribution for the thresholded data. (C) The cumulative explained variance, (D) the eigenvalue distribution and (E) cumulative explained variance of the shuffled data.
**Figure S20:** PCA with the full data set. (A) PCA embedded in two dimensions on the full 16S data. The colours represent the environment. (B–D) The same embedding as in (A), but separated by the environments: cellulose broth, paper and glucose, respectively. The colours represent the 14 days and the markers represent the four rounds of transfer. (E) Eigenvalue distribution, (F) cumulative explained variance, (G) Eigenvalue distribution and (H) cumulative explained variance of shuffled data.
**Figure S21:** Details of PCA on PICRUSt2 output: PICRUSt2 provides three outputs—abundances of EC number, KO and pathways. The logarithm of the frequency of the maximum abundance across all samples, the eigenvalue distribution with an inset of the cumulative explained variance, and the eigenvalue distribution and cumulative explained variance of the shuffled data are shown for (A–C) cellulose broth environment, (D–F) cellulose paper and (G–I) glucose.
**Figure S22:** PCA on Kegg Ortholog abundance output of PICRUSt2: PCA was performed on the KO abundances as described in Methods. (A) The two‐dimensional embedding of the sample, with the colours corresponding to the environment. (B–D) The same embedding as in (A), but separated by the environments: cellulose broth, paper and glucose, respectively. The colours show the 14 days of sampling within each round, and the markers represent the rounds of transfer.
**Figure S23:** PCA on pathway abundance output of PICRUSt2: PCA was performed on the pathway abundances as described in Methods. (A) The two‐dimensional embedding of the sample, with the colours corresponding to the environment. (B–D) The same embedding as in (A), but separated by the environments: cellulose broth, paper and glucose, respectively. The colours show the 14 days of sampling within each round, and the markers represent the rounds of transfer.
**Figure S24:** Embedding Euclidean distances of EC number composition. The Euclidean distances between communities were computed based on the EC number composition, as described in Methods. The pairwise distances were embedded in two dimensions using multidimensional embedding for each environment (A) cellulose broth (B) cellulose paper and (C) glucose. The bottom panels show the stress of the embedding.
**Figure S25:** Euclidean distances of EC composition across replicates. The Euclidean distances between replicates based on EC composition were calculated. These are shown on the *y*‐axis. The *x*‐axis shows the days of sampling and the columns show the four rounds of transfer. The top row is for cellulose broth, the middle row for cellulose paper and bottom row for glucose. Each boxplot represents values across five replicate communities in each environment, where the boxes extend from the first to the third quartile, solid line represents the median, and the whiskers extend from the box to the farthest data point lying within 1.5 times the inter‐quartile range.
**Figure S26:** Embedding Euclidean distances of KO composition. The Euclidean distances between communities were computed based on the KO composition, as described in Methods. The pairwise distances were embedded in two dimensions using multidimensional embedding for each environment (A) cellulose broth (B) cellulose paper and (C) glucose. The bottom panels show the stress of the embedding.
**Figure S27:** Euclidean distances of KO composition across replicates. The Euclidean distances between replicates based on KO composition were calculated. These are shown on the *y*‐axis. The *x*‐axis shows the days of sampling and the columns show the four rounds of transfer. The top row is for cellulose broth, the middle row for cellulose paper and bottom row for glucose. Each boxplot represents values across five replicate communities in each environment, where the boxes extend from the first to the third quartile, solid line represents the median, and the whiskers extend from the box to the farthest data point lying within 1.5 times the inter‐quartile range.
**Figure S28:** Embedding Euclidean distances of pathways composition. The Euclidean distances between communities were computed based on the pathways composition, as described in Methods. The pairwise distances were embedded in two dimensions using multidimensional embedding for each environment (A) cellulose broth (B) cellulose paper and (C) glucose. The bottom panels show the stress of the embedding.
**Figure S29:** Euclidean distances of pathways composition across replicates. The Euclidean distances between replicates based on pathways composition were calculated. These are shown on the *y*‐axis. The *x*‐axis shows the days of sampling and the columns show the four rounds of transfer. The top row is for cellulose broth, the middle row for cellulose paper and bottom row for glucose. Each boxplot represents values across five replicate communities in each environment, where the boxes extend from the first to the third quartile, solid line represents the median, and the whiskers extend from the box to the farthest data point lying within 1.5 times the inter‐quartile range.
**Figure S30:** Eigenvalues PCA on FAPROTAX output. (A) The eigenvalues distribution of the FAPROTAX output, (B) the cumulative explained variance. (C, D) The eigenvalue distribution and cumulative explained variance for the shuffled data.
**Figure S31:** Euclidean distances of functional composition across replicates. The Euclidean distances between replicates based on the functional composition output of FAPROTAX were calculated. These are shown on the *y*‐axis. The *x*‐axis shows the days of sampling and the columns show the four rounds of transfer. The top row is for cellulose broth, the middle row for cellulose paper and bottom row for glucose.
**Figure S32:** Embedding Euclidean distances of functional composition provided by FAPROTAX. The Euclidean distances between communities were computed based on the functional composition output of FAPROTAX, as described in Methods. The pairwise distances were embedded in two dimensions using multidimensional embedding for each environment (A) cellulose broth (B) cellulose paper and (C) glucose. The bottom panels show the stress of the embedding. Each boxplot represents values across five replicate communities in each environment, where the boxes extend from the first to the third quartile, solid line represents the median, and the whiskers extend from the box to the farthest data point lying within 1.5 times the inter‐quartile range.
**Figure S33:** Three‐dimensional embedding of Euclidean distances of functional composition provided by FAPROTAX of glucose communities: The glucose communities were embedded in three dimensions to look for any additional clustering of the samples. (A–C) The embeddings in Dimensions 1 and 2, 1 and 3 and 2 and 3, respectively.
**Figure S34:** Genomes performing the carbon cycle based on metagenomic analyses. Each panel shows the coverage on *y*‐axis (the fraction of genomes in the community that can perform the function shown in the panel title) of the pathway in the carbon cycle, with time on the *x*‐axis.
**Figure S35:** Genomes performing the nitrogen cycle based on metagenomic analyses. Each panel shows the coverage on *y*‐axis (the fraction of genomes in the community that can perform the function shown in the panel title) of the pathway in the nitrogen cycle, with time on the *x*‐axis.
**Figure S36:** Genomes performing the sulphur cycle based on metagenomic analyses. Each panel shows the coverage on *y*‐axis (the fraction of genomes in the community that can perform the function shown in the panel title) of the pathway in the sulphur cycle, with time on the *x*‐axis.
**Figure S37:** Genomes performing other elemental metabolism based on metagenomic analyses. Each panel shows the coverage on *y*‐axis (the fraction of genomes in the community that can perform the function shown in the panel title) of the pathway, with time on the *x*‐axis. The top panel shows iron metabolism, the middle panel arsenate and the bottom panel selenate.
**Table S1:** Enzymes that have significantly higher abundances in the two environments. Derived from 16S data using PICRUSt2, the enzymes whose abundance was higher in CB compared to glucose are listed in the second column and their broad role in the first, and the enzymes whose abundance was higher in the glucose‐supported environment compared to the CB environment are listed in the fourth column and their broad role in the third.
**Table S2:** Kegg Orthologs (KOs) that have significantly higher abundances in the two environments. Derived from 16S data using PICRUSt2, the KOs whose abundance was higher in CB compared to glucose are listed in the second column and their broad role in the first, and the KOs whose abundance was higher in the glucose‐supported environment compared to the CB environment are listed in the fourth column and their broad role in the third.
**Table S3:** Pathways that have significantly higher abundances in the two environments. Derived from 16S data using PICRUSt2, the pathways whose abundance was higher in CB compared to glucose are listed in the second column and their broad role in the first, and the pathways whose abundance was higher in the glucose‐supported environment compared to the CB environment are listed in the fourth column and their broad role in the third.
**Table S4:** Functions that have significantly higher abundances in the two environments. Derived from 16S data using FAPROTAX, the functions whose abundance was higher in CB compared to glucose are listed in the first column, and the functions whose abundance was higher in the glucose‐supported environment compared to the CB environment are listed in the second column.
**Table S5:** Enzymes that have significantly higher abundances in the early and later growth phases in the glucose environment. Derived from 16S data using PICRUSt2, the enzymes whose abundance was higher in the early growth phase compared to the later growth phase are listed in the second column and their broad role in the first, and those whose abundance was higher in the later growth phase compared to the early growth phase are listed in the fourth column and their broad role in the third.
**Table S6:** KOs that have significantly higher abundances in the early and later growth phases in the glucose environment. Derived from 16S data using PICRUSt2, the KOs whose abundance was higher in the early growth phase compared to the later growth phase are listed in the second column and their broad role in the first, and those whose abundance was higher in the later growth phase compared to the early growth phase are listed in the fourth column and their broad role in the third.
**Table S7:** Pathways that have significantly higher abundances in the early and later growth phases in glucose environment. Derived from 16S data using PICRUSt2, the pathways whose abundance was higher in the early growth phase compared to the later growth phase are listed in the second column and their broad role in the first, and those whose abundance was higher in the later growth phase compared to the early growth phase are listed in the fourth column and their broad role in the third.
**Table S8:** Functions that have significantly higher abundances in the early and later growth phases. Derived from 16S data using FAPROTAX, the functions whose abundance was higher in the early growth phase compared to the later growth phase are listed in the first column, and those whose abundance was higher in the later growth phase compared to the early growth phase are listed in the second column.

**File S1:** Combined networks_modules_positive.

**File S2:** Combine_netowrks_modules_negative.

## Data Availability

All sequence data are openly available in a public repository. The amplicon sequence data are available at: https://doi.org/10.5281/zenodo.16760758 and the metagenomic data are available at: https://doi.org/10.17617/3.INEIFJ

## Appendix A

### Alpha Diversity—Diversity Metrics

Diversity was calculated using four metrics for all samples without removing rare ASVs, except for Faith's PD.

#### Shannon Diversity

The Shannon diversity metric (Spellerberg and Fedor 2003; Shannon 1948), H, is defined by Equation (A1).

(A1)
$$H=-\sum_{i}p_{i}\log_{2}p_{i}.$$

Here, $p_{i}$ is the relative abundance of ASV_*i*_ in the community. This metric was calculated using skbio's “Shannon” function (Aton et al. 2026).

#### Abundance‐Based Coverage Estimator

The Abundance‐based Coverage Estimator (ACE) metric $S_{\mathrm{ace}}$ (Chao et al. 2000) measures richness and is given by Equation (A2).

(A2)
$$S_{\mathrm{ace}}=S_{\text{abund}}+\frac{S_{\text{rare}}}{C_{\mathrm{ACE}}}+\frac{F_{1}}{C_{\mathrm{ACE}}}\gamma_{\mathrm{ACE}}^{2}.$$

The ASVs are classified as abundant, $S_{\text{abund}}$, and rare, $S_{\text{rare}}$, depending on whether their count is greater than or less than 10, respectively. $F_{i}$ is the number of species with a count of i (i.e., $F_{1}$ is the number of species with count 1). $C_{\mathrm{ACE}}=1-\frac{F_{1}}{N_{\text{rare}}}$ is the sample coverage, where $N_{\text{rare}}=\sum_{i=1}^{10}{\text{iF}}_{i}$ is the total number of counts in rare species. $\gamma_{\mathrm{ACE}}$ is the estimated coefficient of variation for the rare ASVs, given by $\gamma_{\mathrm{ACE}}^{2}=\max\left[\frac{S_{\text{rare}}}{C_{\mathrm{ACE}}}\frac{\sum_{i=1}^{10}i\left(i-1\right)F_{i}}{N_{\text{rare}}\left(N_{\text{rare}}-1\right)}-1,0\right]$. This metric was computed using skbio's “ace” function (Aton et al. 2026) based on the EstimateS manual by Colwell (Colwell and Elsensohn 2014).

#### Simpson's Evenness

Simpson's evenness measure (Simpson 1949), *E*, quantifies how even the samples are. It is defined by Equation (A3).

(A3)
$$E=\frac{1/D}{S_{\mathrm{obs}}}.$$

Here, $\frac{1}{D}$ is the effective number of species, where $D=\sum_{i=0}^{S_{\mathrm{obs}}}p_{i}^{2}$. As before, $p_{i}$ is the relative abundance of ASV in a sample. $S_{\mathrm{obs}}$ is the number of unique ASVs observed. This measure was calculated using skbio's (Aton et al. 2026) “simpson_e” function.

#### Faith's Phylogenetic Diversity

To include the phylogenetic information, we used the Faith's Phylogenetic Diversity (PD) metric. We used scikit‐bio's (Aton et al. 2026) implementation of Faith's PD metric, which is based on (Hamady et al. 2010). The phylogenetic information is input in the form of a tree. As it is difficult to align 15,576 sequences, we reduced our data to include only those taxa that have a maximum relative abundance of at least 0.5% in any sample. This reduced the dataset to 727 ASVs. The fasta file containing the sequences of these ASVs were aligned and a tree created using SILVA's ACT feature (Pruesse et al. 2012) with the FastTree algorithm. This tree was used as the input for skbio's Faith's PD calculation.

### Principal Component Analysis

In order to perform the PCA, it is necessary to take into account the compositional nature of the relative abundance data (Gloor et al. 2016, 2017).

#### Zeroes and Rare Taxa

One of the log transformations introduced by (Aitchison et al. 2000), the centered log ratio (clr) transform was used here to overcome the limitations posed by the compositional nature of the data. The “clr” function from skbio was used to calculate the transform. However, one problem with using such log transformations is the presence of zeros in the data. This problem was overcome here by adding a small number, 0.001, to all the data. It has been shown (Silverman et al. 2017) that adding a small pseudocount does not impact further analyses. To further discard rare taxa, with low counts, the maximum count of each ASV across all samples was plotted (Figure S19A). This shows that there are many taxa whose maximum abundance across all samples is very low. Any ASV whose maximum count was less than 1350 was removed (vertical dashed line in Figure S19A). Given the average counts per sample, 24,044, and the standard deviation is about 27% (Figure S1), this implies removing ASVs whose maximum relative abundance across all samples is less than about 5.6%. With this thresholding, the number of sequences reduces from 15,576 to 102.

#### Choosing the Number of Dimensions

PCA was performed on the reduced dataset using scikit‐learn (Pedregosa et al. 2011). The distribution of eigenvalues is plotted in Figure S19B, where it is noted that the data had two dominant eigenvalues, while the rest were comparatively low. The first two eigenvalues explain 40.7% and 13.2% of the variance. Figure S19C shows the cumulative explained variance. These indicated that the first two dimensions explained the data well. To further confirm this, the data were shuffled by randomizing the counts for each sample. The clr transform was performed on this randomized dataset. By performing PCA on this randomized clr‐transformed dataset, the eigenvalue distribution of the randomized dataset was obtained (Figure S19D,E). The two dominant eigenvalues observed before were absent, indicating that the structure of the dataset is lost on randomizing.

#### PCA Without Removal of Rare Taxa

PCA was performed on the complete data, without removing any rare taxa, after performing the clr transform (Figure S20). The eigenvalue distribution was similar, with the first two eigenvalues much higher than the rest, although in this case, the third eigenvalue was also significant (Figure S20E). However, the first two components only explained 17.2% and 9.1% of the variance (Figure S20F). The basic structure of data remained the same as in the PCA with rare taxa removed, both across and within the environment (Figure S20A–D), especially with regard to the separation along PC1 and the succession dynamics. Random shuffling of the data caused it lose its structure, with the high eigenvalues dropping out (Figure S20G,H).

#### Contributors to Separation Along PC1

The contributions of the ASVs to the separation of the communities grown on glucose broth from the communities grown on cellulose along PC1 were calculated as follows (Prabhakara and Kuehn 2023). The eigenvector along PC1 is $\vec{\mathrm{PC}1}=\left\{l_{1}^{1},l_{2}^{1},l_{3}^{1},\ldots ,l_{k}^{1},\ldots \right\}$, where $l_{k}^{1}$ is the loading corresponding to ASV k along PC1. The superscript 1, refers to PC1. The composition of a sample is the vector, $\vec{c}=\left\{x_{c}^{1},x_{c}^{2},x_{c}^{3},\ldots ,x_{c}^{k},\ldots \right\}$, where $\vec{c}$ represents a sample and $x_{c}^{k}$ is the mean subtracted, clr‐transformed abundance corresponding to ASV k in the sample represented by $\vec{c}$. The distance between two communities along PC1 is $d=\;|\left\langle {\vec{c}}_{\text{cellulose}}|\vec{\mathrm{PC}1}\right\rangle -\left\langle {\vec{c}}_{\text{glucose}}|\vec{\mathrm{PC}1}\right\rangle |\;=\sum_{k}|\left(x_{\text{cellulose}}^{k}-x_{\text{glucose}}^{k}\right)|l_{k}^{1}$, where ${\vec{c}}_{\text{cellulose}}$ represents a community grown on cellulose and ${\vec{c}}_{\text{glucose}}$ represents a community grown on glucose. The summation is over all the ASVs k. $\left\langle \vec{p}|\vec{q}\right\rangle$ denotes the inner product of the two vectors $\vec{p}$ and $\vec{q}$ and can be interpreted as the projection of $\vec{p}$ on $\vec{q}$. Therefore, the contribution of ASV k to the separation of the communities grown in glucose broth from the communities grown in cellulose along PC1 is $o_{k}=\;|\left(x_{\text{cellulose}}^{k}-x_{\text{glucose}}^{k}\right)|l_{k}^{1}$.

The $o_{k}$ for each ASV k was computed by considering all possible pairs of communities grown in glucose broth and cellulose broth across all replicates, transfers and days. The median across all these pairs was used as the contribution of the ASV to separation along PC1. The cumulative distribution of $o_{k}$ was obtained for all ASVs, and a cut‐off value was chosen to be at 90 percentile. The ASVs whose $o_{k}$ was higher than this cutoff were designated as those contributing the most to the separation of the communities grown on glucose from the communities grown on cellulose along PC1. The clr‐transformed abundance of these ASVs are plotted in Figure 4E.

#### Contributors to Displacement Along PC2

From Figure 4B–D, it is clear that the samples move down along PC2 in all three cases. This is especially clear from Round 2 when the initial transients have ended. To know which ASVs contribute the most to this displacement along PC2, a similar method as before was applied.

The eigenvector along PC2 is $\vec{\mathrm{PC}2}=\left\{l_{1}^{2},l_{2}^{2},l_{3}^{2},\ldots ,l_{k}^{2},\ldots \right\}$, where $l_{k}^{2}$ is the loading corresponding to ASV k along PC2. The superscript 2, refers to PC2. As before, the composition of a sample is the vector, $\vec{c}=\left\{x_{c}^{1},x_{c}^{2},x_{c}^{3},\ldots ,x_{c}^{k},\ldots \right\}$, where $\vec{c}$ represents a sample and $x^{k}$ is the mean subtracted, clr‐transformed abundance corresponding to ASV k in the sample represented by $\vec{c}$. The distance between two communities along PC2 is $d=\;|\left\langle {\vec{c}}_{R4}|\vec{\mathrm{PC}2}\right\rangle -\left\langle {\vec{c}}_{R2}|\vec{\mathrm{PC}2}\right\rangle |\;=\sum_{k}|\left(x_{R4}^{k}-x_{R2}^{k}\right)|l_{k}^{2}$, where ${\vec{c}}_{R2}$ represents a community at Round 2 and ${\vec{c}}_{R4}$ represents a community at Round 4. The summation is over all the ASVs k. Therefore, the contribution of ASV k to the displacement of the communities across Rounds along PC2 is $o_{k}=\;|\left(x_{R4}^{k}-x_{R2}^{k}\right)|l_{k}^{2}$.

The $o_{k}$ for each ASV k was computed by considering all possible pairs of communities in Rounds 2 and 4 across all replicates, and days, but within a given environment. The median across all these pairs was used as the contribution of the ASV to displacement along PC2. The cumulative distribution of $o_{k}$ was obtained for all ASVs, and a cut‐off value was chosen to be at 90 percentile. The ASVs whose $o_{k}$ was higher than this cutoff were designated as those contributing the most to the displacement along PC2. The clr‐transformed abundance of these ASVs are plotted in Figure S18.

### Beta Diversity—Distance Metrics

Three distance metrics were computed for the data. The entire dataset was used for these computations, that is, no ASVs were removed, except for Unifrac metric.

#### Jensen–Shannon Divergence

Due to the compositional nature of the data, the composition of the samples can be viewed as probability distributions, summing to 1. The JSD (Lin 1991) is symmetric, bounded and calculates distance between normalized probability distributions. Here, it was calculated using Scipy's (Virtanen et al. 2020) jensenshannon function. The JSD between two distributions X and Y, $J_{X,Y}$ is defined in Equation (A4).

(A4)
$$J_{X,Y}=H\left(\pi_{1}X+\pi_{2}Y\right)-\pi_{1}H\left(X\right)-\pi_{2}H\left(Y\right).$$

If $X=\left\{x_{i}\right\}$ and $Y=\left\{y_{i}\right\}$, where $x_{i}$ and $y_{i}$ represent the normalized probability of finding the value $x_{i}$ and $y_{i}$ in the probability distributions X and Y, then the function H is the entropy of the distribution defined by Equation (A5), similar to Equation (A1).

(A5)
$$H\left(X\right)=-\sum x_{i}\log x_{i}.$$

The weights $\pi_{1}$ and $\pi_{2}$ are set to be equal, that is, $\frac{1}{2}$. Using Equations (4) and (5), the JSD between the relative abundance composition of the two communities can be calculated. Note that the Scipy function “jensenshannon” calculates the square root of Equation (A4). These values are reported in Figure S9.

#### Aitchison's Distance Metric

Aitchison's distance is a relevant metric for calculating distances between samples in compositional data (Gloor et al. 2016, 2017). A small pseudocount (0.001) was added to all the data to remove zeroes. The data were then transformed via clr. The Euclidean distance between the samples was then computed using the “euclidean” function from Scipy. These distances are reported in Figure S11.

#### Unifrac Distance Metric

To include the phylogenetic information in calculating the distances between communities, Unifrac distance metric was used. This metric needs a tree as the input. The same tree created for Faith's PD measurement was used here. As before, the data set was reduced to contain only those taxa whose maximum relative abundance was atleast 0.5% in any sample. Skbio's unweighted unifrac function, which is based on (Hamady et al. 2010), was used to compute the Unifrac distances. These distances are reported in Figure S13.

#### Embedding Distances

In order to visualize distances between the communities better, multi‐dimensional embedding (MDS) was used to embed the distances in two dimensions. However, since apriori the dimensionality is unknown, the stress of embedding was calculated for 1–5 dimensional spaces. These stresses are shown in the bottom panel of each embedding in Figures S10, S12 and S14. These show that the stress of a two dimensional embedding is close to the asymptotic value. Scikit‐learn's “MDS” function was used to perform the embedding. Note that each embedding was performed independently, so that coordinates across different embeddings cannot be compared.

### Correlation Networks

SCNIC (Shaffer et al. 2023) was used to obtain correlation modules and networks in the data. To enable interpretation, the data were thresholded to include only those taxa whose relative abundance was at least 5% in any community at any time point. This thresholded dataset was then subdivided into the three environments, CP, CB, and glucose, and these were provided as inputs to the SCNIC package. As a first step, SCNIC uses a fast implementation of the package sparcc (Friedman and Alm 2012), to calculate correlations between all ASVs within each environment. sparcc takes into account the compositional nature of the data to estimate the correlation strengths. Next, SCNIC takes those ASVs whose correlation strength is 0.35 or higher, and groups them in modules so that each ASVs in the module is correlated to all other ASVs in the module via a correlation strength of 0.35 or higher. By such a definition, some ASVs cannot be placed in any modules and are indicated by light blue patches in the Files S1 and S2. Cytoscape (Shannon et al. 2003) was used to create the network maps. Currently, SCNIC only works with positive correlations. In order to create modules of negative correlations, the signs of correlation strengths were inverted and this modified data was provided to SCNIC to create modules.

The correlation network data files were parsed using NetworkX (Hagberg et al. 2008) in python to obtain the values of the strengths and number of correlations for comparing across environments.

Although the number of positive and negative interactions per taxon were strongly correlated (Pearson *r* ∼0.8, *p*‐value $∼1\times 10^{-29}$), the strengths of these interactions were not. For all taxa, the number of interactions was positively correlated with the average strength, for both positive and negative interactions (Pearson *r* ∼0.6, *p*‐values <0.0003), except for positive interactions in glucose, where no correlation was observed. In glucose, ASVs showed significantly stronger positive than negative interactions (*p*‐value <0.003), whereas in CB and CP, the strengths of positive and negative interactions were similar.

Comparing across environments, ASVs on CP exhibited significantly more positive interactions than those in glucose or CB (*p*‐values <0.007), and significantly more negative interactions than ASVs in CB (*p*‐value $<1\times 10^{-5}$). This indicates that the paper environment supported more interactions than the broth. Positive and negative interaction strengths were positively correlated between CB and CP (Pearson *r* ∼0.8 and *r* ∼0.6, with *p*‐values $∼7\times 10^{-6}$ and 0.006, respectively).

### Functional Analysis

From the 16S sequence data, it is possible to get insights into the functional profiles of the communities. Here, two pipelines were used to obtain clues about the functions of the communities.

#### PICRUSt2

The package PICRUSt2 (Douglas et al. 2020) was used to infer Enzyme Classification (EC) numbers, KOs and pathway abundances in the samples. These will be referred to as ‘function types’. For all three cases, the unstratified output of PICRUSt2 was used, which provides abundances of each function per sample. In particular, this is a table for each function type where, for each sample, the abundance of a function is estimated. Here, by function we mean EC numbers for the function type Enzymes, KO numbers for the function type KOs and pathway names for the function type pathways.

**Comparison across environments**

For each function type, the abundance distribution of each function was compared between (1) all the cellulose broth communities and all the glucose broth communities and (2) all the cellulose paper communities and all the glucose broth communities. A two sample Kolmogorov–Smirnoff test was performed to estimate if the distribution of a function was significantly different between the two environments. For those functions that were significantly different, the difference in the median value of the function between the two environments was found. Based on this difference, the 15 functions that were high in cellulose broth and had the highest difference compared to glucose broth were chosen. Similarly, the 15 functions that were high in glucose and had the highest difference compared to cellulose broth were chosen. These are represented in Tables S1–S3. The functions selected from the comparison between cellulose paper and glucose were almost identical to those selected from the comparison between cellulose broth and glucose. Here only the latter are shown.

**Distance metric**

Euclidean distance was computed based on the abundance of functions between communities in a given environment, for each function type. Since the data are not compositional, the Euclidean metric can be directly applied. The entire dataset was used for this. The distance between replicates at each day is shown in Figures S25, S27 and S29 for the function‐type enzymes, KOs and pathways, respectively. The distances were embedded in two dimensions using the MDS method described above. These are shown in Figures S24, S26 and S28 for the function type enzymes, KOs and pathways, respectively. Below each embedding, the stress of the MDS embedding is shown. The stress at two dimensions is similar to the stress at higher dimensions; so a two‐dimensional representation is acceptable in these cases.

**Comparison of communities between the initial and the final days of the cycles in glucose broth**

From Figures S24, S26 and S28 it was noted that in the glucose broth, the communities in the initial days of all four rounds clustered together (blue markers of all four types). To look for the differences in the function between the initial days and the final days, the communities at Days 3 and 4 of all Rounds were grouped as the ‘initial days’ and the communities at Days 13 and 14 of all Rounds were grouped as the ‘final days’. Days 3 and 4 were chosen instead of Days 1 and 2 to avoid transient dynamics. As before, for each function type, the abundance distribution of each function was compared between the initial days and the final days to check if they were significantly different between the two groups. The two sample Kolmogorov–Smirnoff test was used to test for significance, with a Bonferroni‐corrected *p*‐value. For the functions that were significantly different, the median across all communities in each group was found to know in which group the function is higher. Based on the difference in the medians, the functions were classified as being higher in the initial days and higher in the final days. This is shown in Tables S1–S3 for the three function types.

**Principal component analysis**

PCA was performed on the data in the following way. First, the dataset was thresholded to remove very low abundance functions. The frequency of the maximum abundance of each function type across all samples was plotted (Figure S21) and a threshold was set for each function type to remove those functions whose maximum abundance was very low across all samples. This reduced the number of functions: from 2413 to 610 for the enzymes, from 7802 to 1396 for the KOs and from 438 to 361 for the pathways. Since these data are not compositional, first a PCA was performed directly on the dataset, and a distribution of the eigenvalues was found. Next, the data were randomized by shuffling the columns (functions) randomly for each row (samples). PCA was performed on this randomized dataset. However, on comparing the eigenvalue distribution, it was found that the highest eigenvalue remained in the randomized dataset, indicating higher order structure to the data. A careful analysis revealed that some of the samples had almost an order of magnitude higher total function (e.g., total enzyme abundances) than others. To correct for this, the abundances were converted to relative abundances. PCA was then performed on the clr‐transformed data as described before. Now, when the eigenvalue distributions of the data were compared to the randomized data, the high eigenvalues disappeared (Figure S21C,F,I), indicating that the difference in total function abundances was indeed the reason why the highest eigenvalue was retained when the data were not compositional. Further, the eigenvalue distributions also showed that the first two principal components are the largest and could adequately describe the dataset. Figure 5A shows the PCA on the data with the function type enzymes. Figures S22 and S23 show the PCA on the data with function type KOs and pathways, respectively. The percentage of variance explained is indicated on the axes of the plots.

Similar to the analysis of the 16S data, here the contribution of each function to the separation along PC1 was found for each function type. The top 98th percentile of functions that contribute to the separation were chosen. The clr of the abundances of these functions for all three function types in the cellulose broth and glucose broth environments are shown in Figure 5B–D.

#### FAPROTAX

The package FAPROTAX (Louca et al. 2016) was used to analyse the 16S data. This package provides function predictions based on the 16S sequences. The output is a file that provides the abundance of each function in each community.

#### Comparison Across Environments

Similar to the analysis using PICRUSt2, the distribution of abundances of each function was compared between communities in (1) cellulose broth and glucose broth and (2) cellulose paper and glucose broth. The two sample Kolmogorov–Smirnoff test was used to test the null hypothesis that both samples were drawn from the same distribution. If the *p*‐value was lower than the Bonferroni‐corrected threshold, then the null hypothesis was incorrect and the two samples are differently distributed. The medians across all samples within a group of the functions that were found to be differently distributed were found. Based on the difference in the medians, the functions were classified as either being higher in the cellulose broth environment or in the glucose environment. The functions are presented in Table S4. These were identical to the functions selected in the comparison between communities grown on cellulose paper and glucose broth.

**Distance metric**

Euclidean distances were computed between all pairs of communities as described above. The distance between the replicates for each day is shown in Figure S31. The distances within each environment were embedded on two dimensions using multi‐dimensional embedding, as shown in Figure S32. The stress of the embedding is indicated below each panel.

As can be seen from the embedding, for glucose the stress at two dimensions is considerably higher than at three dimensions. To check for clustering or separation at higher dimensions, the glucose communities were embedded in three dimensions. This is shown in Figure S33.

**Comparison of communities between the initial and the final days of the transfer Rounds in glucose broth**

As seen in Figure S32C, the glucose communities are clustered according to the day of growth across all transfer Rounds, that is, there is a transition from blue to yellow markers. To investigate this further, the abundance distribution of each function was compared across two groups—the ‘initial days’ group consisting of all communities at Days 3 and 4, and the ‘final days’ group consisting of all communities at Days 13 and 14. As before, the first 2 days were not considered to avoid transient dynamics. The Kolmogorov–Smirnoff test was used, with a Bonferroni‐corrected *p*‐value, to determine if the distribution of abundances was significantly different between the two groups. For those functions where it was different, the median of the function abundance was found across each group. The difference in medians between the two groups was used to determine in which group the function was dominant. This is shown in Table S4.

**Principal component analysis**

PCA was performed on the dataset, without any transformation of the data. The eigenvalue distribution is shown in Figure S30. The dataset was then randomized as described above and PCA performed on the randomized dataset. This showed that the two dominant eigenvalues disappeared on randomizing. So no further transformation of the data was needed. The results of the PCA are shown in Figure 6A–D.

In this case, the glucose and cellulose communities are separated along PC2. As described before, the contribution of each function to the separation along PC2 was found. The top 18 contributing functions were chosen. Their abundance across the glucose and cellulose communities is shown in Figure 6E.
