# Supplementary material for: Diverse Microbial Communities Assemble on Both Recalcitrant and Labile Carbon Sources
Source: Environ Microbiol. 2026 Jun 25;28(7):e70351. doi: 10.1111/1462-2920.70351 (PMC13305152; doi:10.1111/1462-2920.70351)
Supplement: Supplementary file 1 — Figure S1: Number of reads per sample. The number of counts plotted on the y‐axis for each sample sequenced, plotted on the x‐axis. Figure S2: Dynamics of dominant taxa in cellulose broth communities. The dynamics of the dominant taxa in cellulose broth as defined in Methods for other four replicates are shown here. The y‐axis shows the relative abundance and the x‐axis shows the days of sampling. The columns are the Rounds of transfer. Below each column the legends show the identity of the ASVs in terms of the lowest phylogenetic classification provided by SILVA. G denotes Genus, F denotes Family. The numbers at the end are unique identifiers for each ASV detected. Figure S3: Dynamics of dominant taxa on cellulose paper communities. The dynamics of the dominant taxa on cellulose paper as defined in Methods for other four replicates are shown here. The y‐axis shows the relative abundance and the x‐axis shows the days of sampling. The columns are the rounds of transfer. Below each column the legends show the identity of the ASVs in terms of the lowest phylogenetic classification provided by SILVA. G denotes Genus, F denotes Family. The numbers at the end are unique identifiers for each ASV detected. Figure S4: Dynamics of dominant taxa in glucose communities. The dynamics of the dominant taxa in glucose as defined in Methods for other four replicates are shown here. The y‐axis shows the relative abundance and the x‐axis shows the days of sampling. The columns are the rounds of transfer. Below each column the legends show the identity of the ASVs in terms of the lowest phylogenetic classification provided by SILVA. G denotes Genus. The numbers at the end are unique identifiers for each ASV detected. Figure S5: Number of taxa within given relative abundance ranges. The number of taxa in relative abundance ranges above 10%, denoted by circles, between 5% and 10% denoted by triangles, and between 1% and 5% denoted by asterisks, are plotted by averaging the abundances acr [file EMI-28-e70351-s003.pdf]

Supplementary Information: *Diverse microbial communities assemble on both recalcitrant and labile carbon sources*

Kaumudi H. Prabhakara, Yansong Zhao, Kristian Ullrich
Andrew D. Farr, Paul B. Rainey

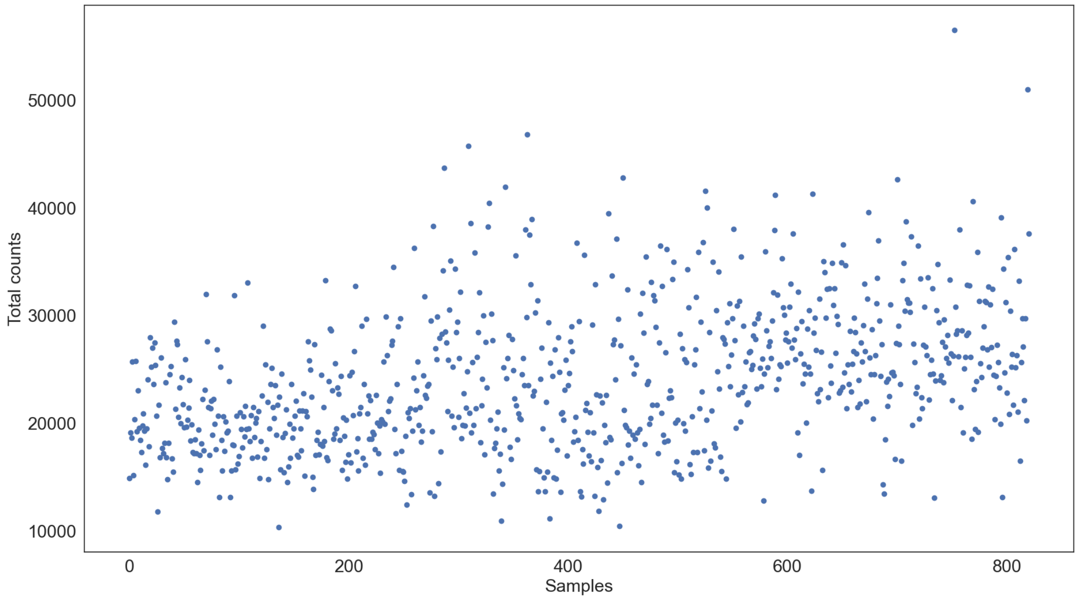

Figure S1: Number of reads per sample: The number of counts plotted on the y axis for each sample sequenced, plotted on the x axis.

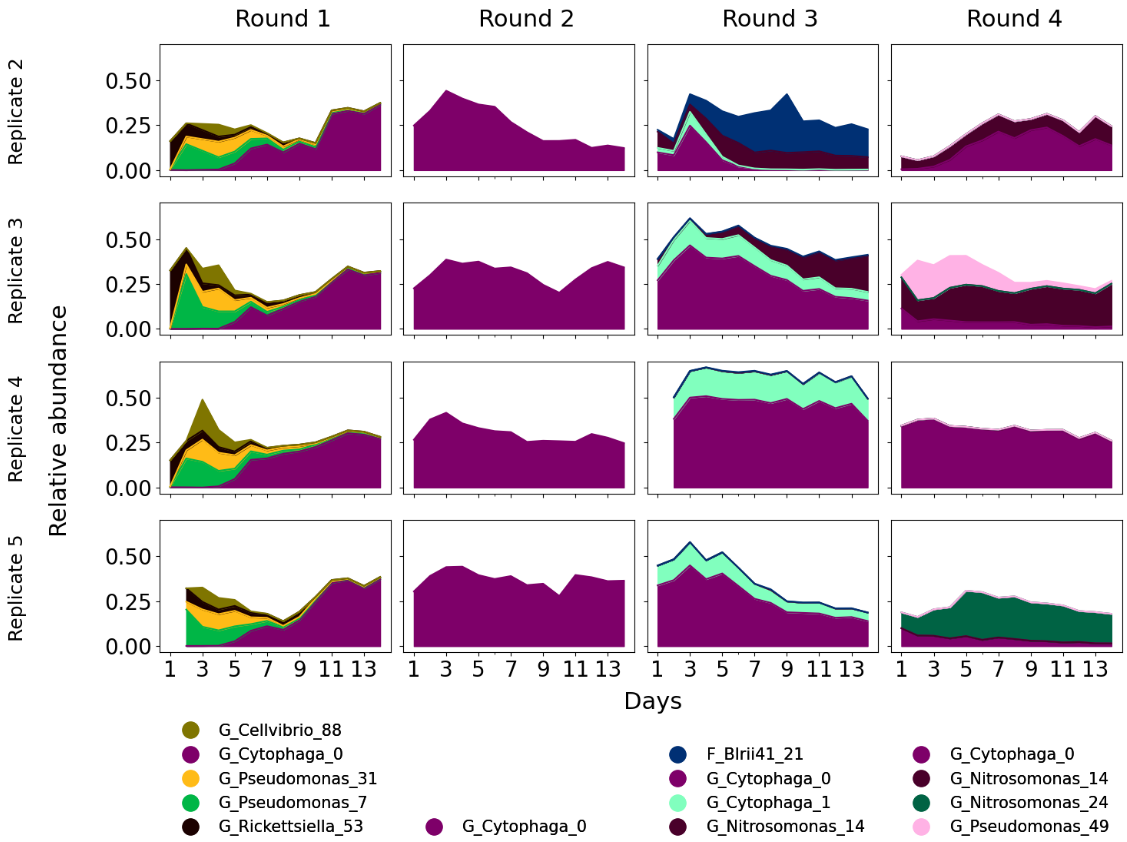

Figure S2: Dynamics of dominant taxa in cellulose broth communities: The dynamics of the dominant taxa in cellulose broth as defined in Methods for other 4 replicates are shown here. The y axis shows the relative abundance and the x axis shows the days of sampling. The columns are the rounds of transfer. Below each column the legends show the identity of the ASVs in terms of the lowest phylogenetic classification provided by SILVA. G denotes Genus, F denotes Family. The numbers at the end are unique identifiers for each ASV detected.

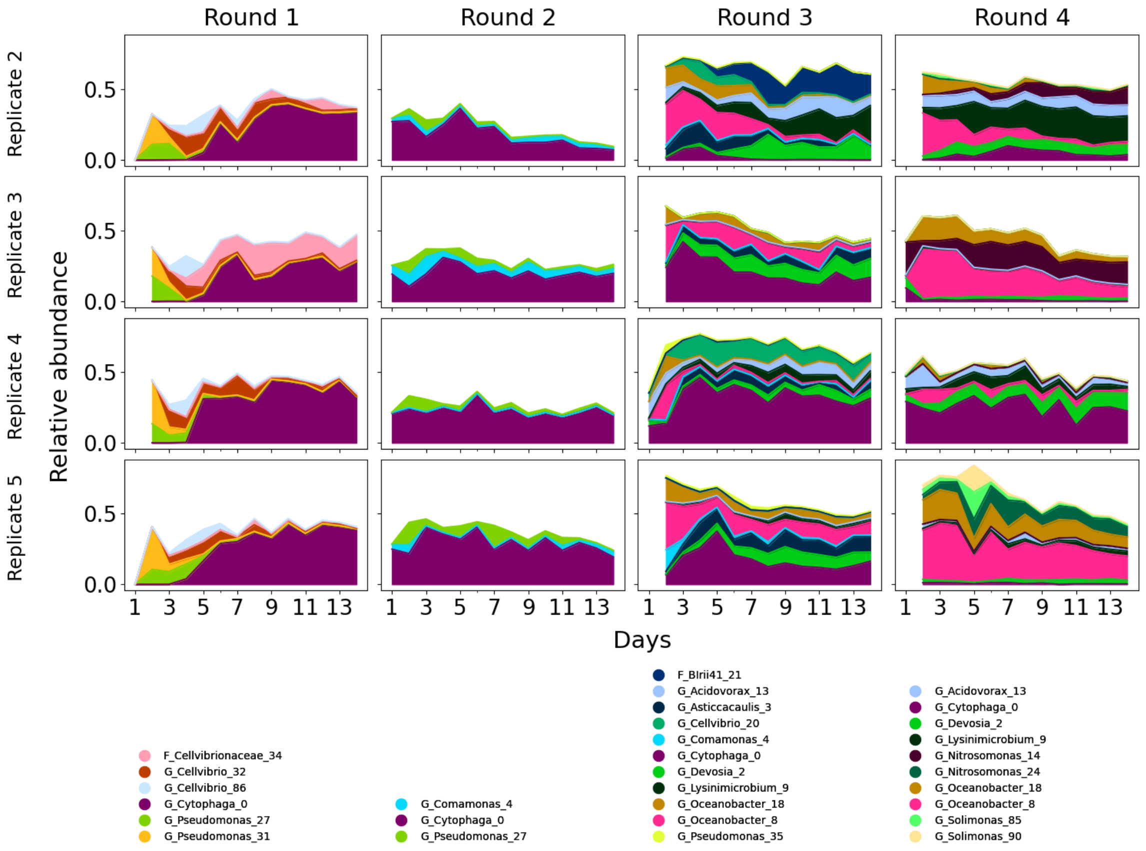

Figure S3: Dynamics of dominant taxa on cellulose paper communities:.The dynamics of the dominant taxa on cellulose paper as defined in Methods for other 4 replicates are shown here. The y axis shows the relative abundance and the x axis shows the days of sampling. The columns are the rounds of transfer. Below each column the legends show the identity of the ASVs in terms of the lowest phylogenetic classification provided by SILVA. G denotes Genus, F denotes Family. The numbers at the end are unique identifiers for each ASV detected.

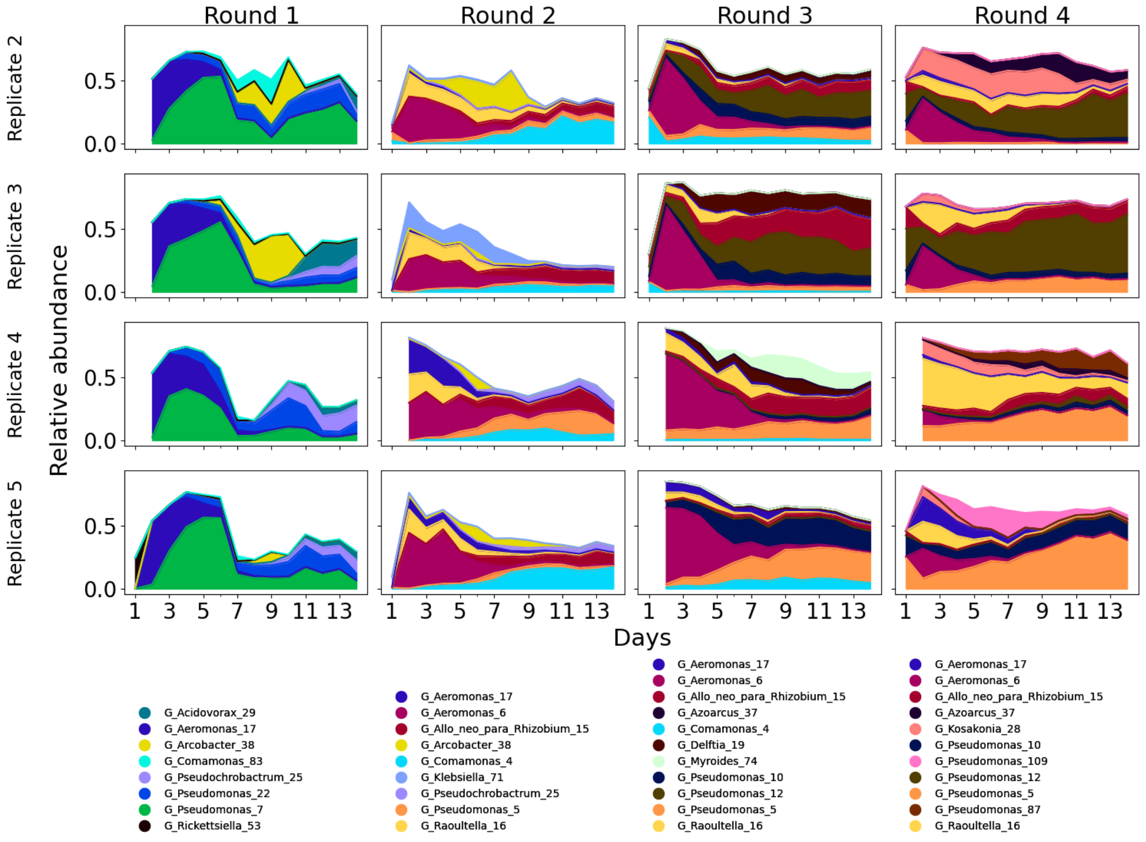

Figure S4: Dynamics of dominant taxa in glucose communities: The dynamics of the dominant taxa in glucose as defined in Methods for other 4 replicates are shown here. The y axis shows the relative abundance and the x axis shows the days of sampling. The columns are the rounds of transfer. Below each column the legends show the identity of the ASVs in terms of the lowest phylogenetic classification provided by SILVA. G denotes Genus. The numbers at the end are unique identifiers for each ASV detected.

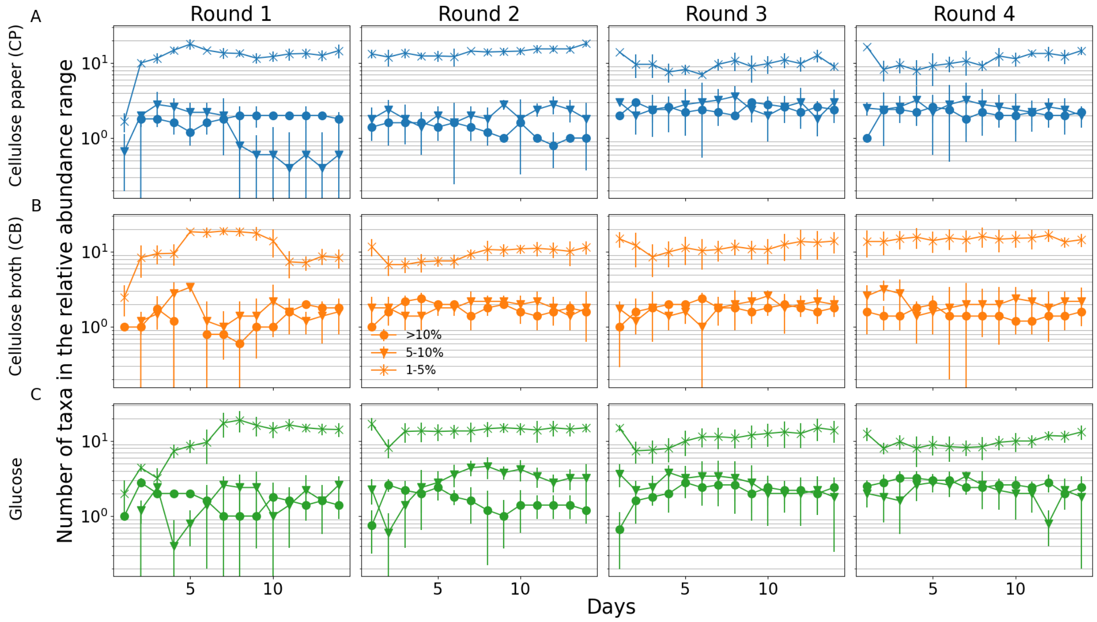

Figure S5: Number of taxa within given relative abundance ranges: The number of taxa in relative abundance ranges above 10%, denoted by circles, between $5-10\%$ denoted by triangles, and between $1-5\%$ denoted by asterisks, are plotted by averaging the abundances across replicates. The top row shows these data for communities in cellulose broth, the middle row for communities on paper, and the bottom row for communities on glucose.

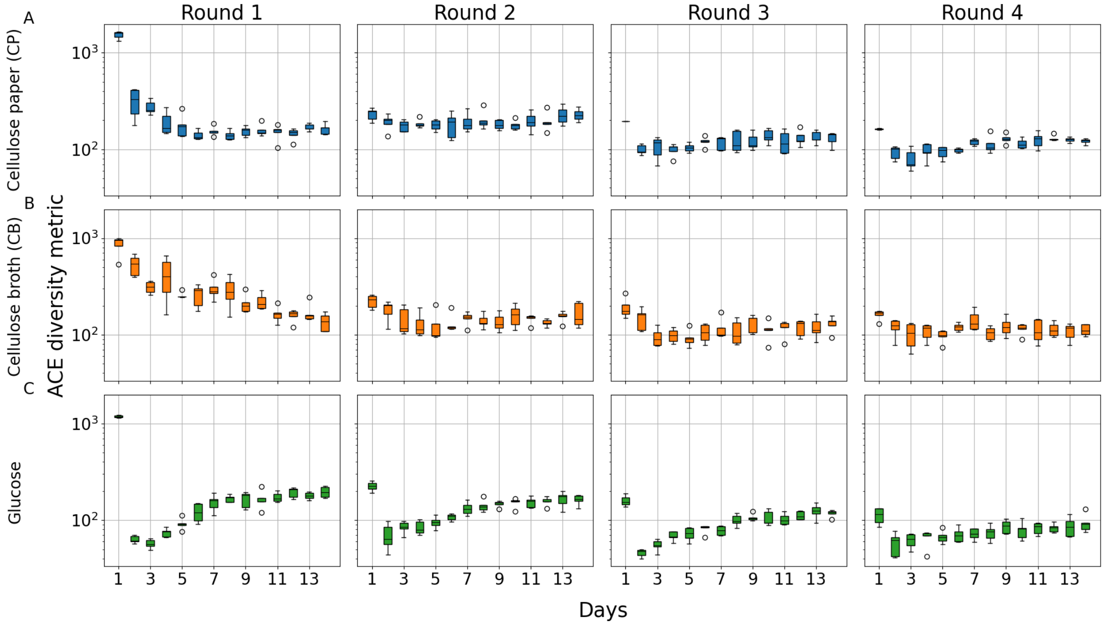

Figure S6: ACE diversity metric: The alpha diversity metric, Abundance based Coverage Estimate, which represents the richness, is shown on the y axis. The x axis shows the days of sampling. Each column shows the rounds of transfer. The top row shows these data for communities in cellulose broth, the middle row for communities on paper, and the bottom row for communities on glucose.

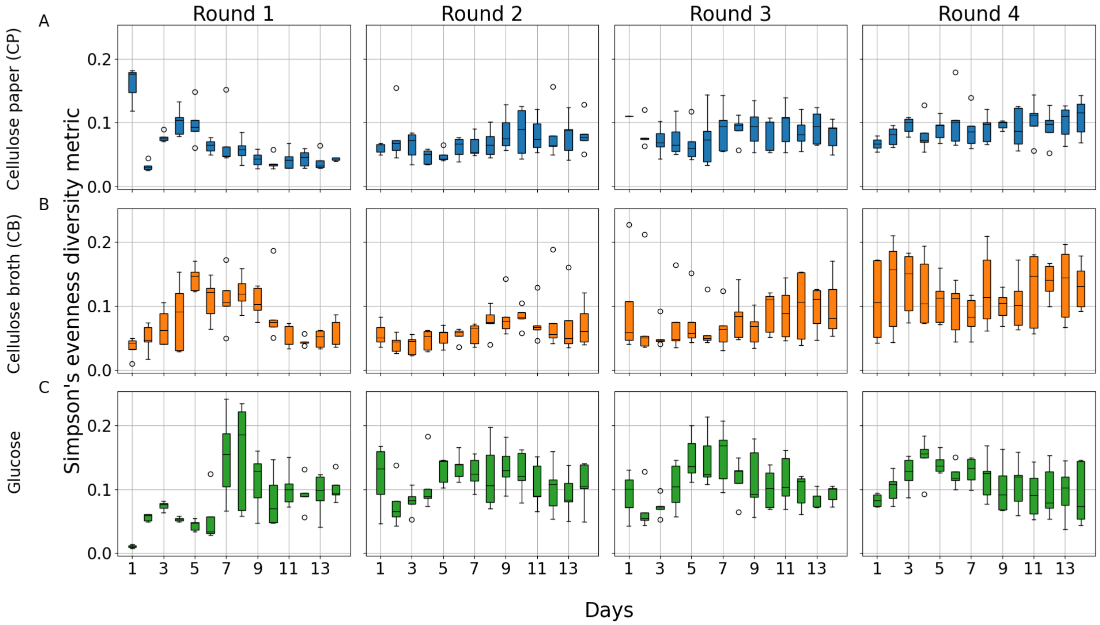

Figure S7: Simpson's evenness measure: The alpha diversity metric, Simpson's evenness, which represents the evenness, is shown on the y axis. The x axis shows the days of sampling. Each column shows the rounds of transfer. The top row shows these data for communities in cellulose broth, the middle row for communities on paper, and the bottom row for communities on glucose.

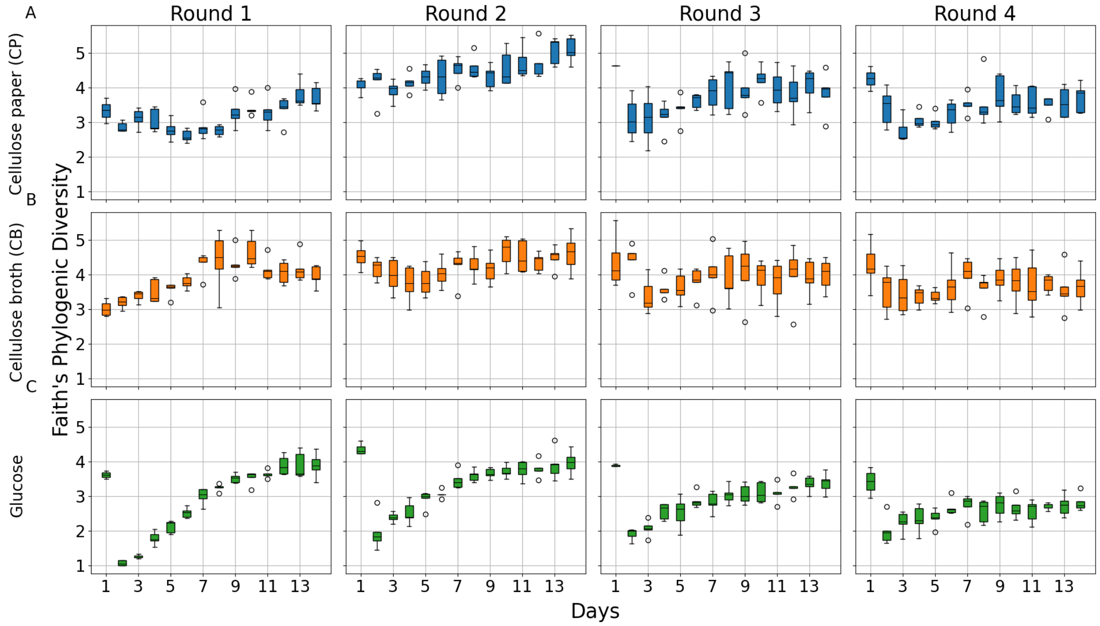

Figure S8: Faith's phylogenetic diversity: The alpha diversity metric, Faith's PD, which takes into account the phylogenetic information, is shown on the y axis. The x axis shows the days of sampling. Each column shows the rounds of transfer. The top row shows these data for communities in cellulose broth, the middle row for communities on paper, and the bottom row for communities on glucose.

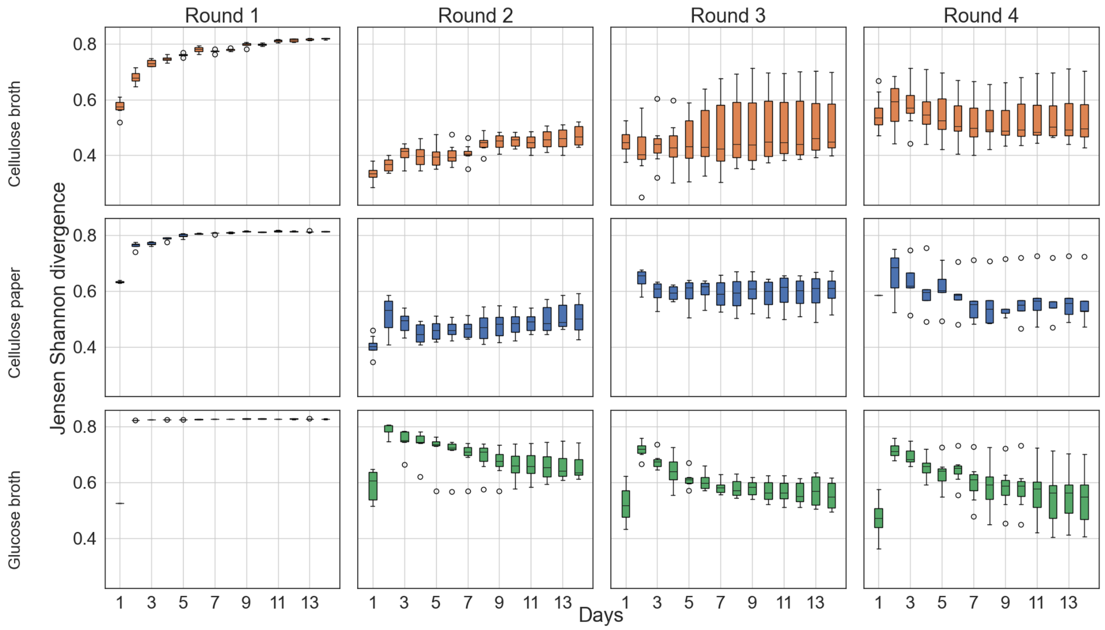

Figure S9: Jensen Shannon divergence (JSD): The beta diversity metric, JSD, is plotted on the y axis for the replicates. The x axis shows the days of sampling. Each column shows the rounds of transfer. The top row shows these data for communities in cellulose broth, the middle row for communities on paper, and the bottom row for communities on glucose. Each boxplot represents values across 5 replicate communities in each environment, where the boxes extend from the first to the third quartile, solid line represents the median, and the whiskers extend from the box to the farthest data point lying within 1.5 times the inter-quartile range.

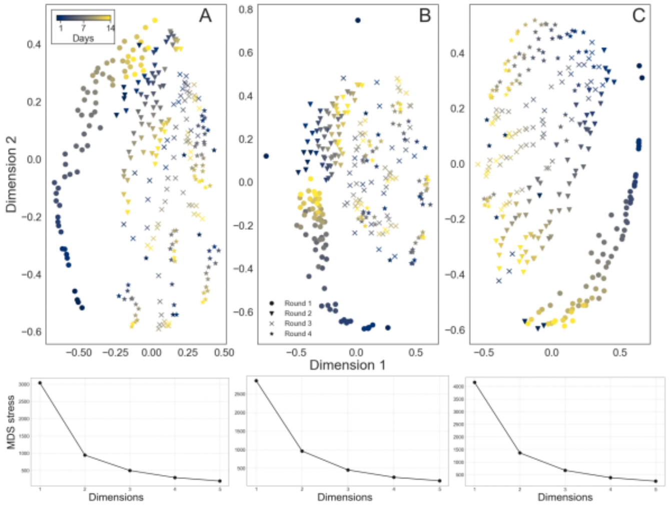

Figure S10: Embedding of Jensen Shannon Divergence metric: The JSD between all samples were embedded in two dimensions for each environment. **A** for cellulose broth **B** for paper and **C** for glucose. The bottom panels show the stress of the MDS embedding in each environment.

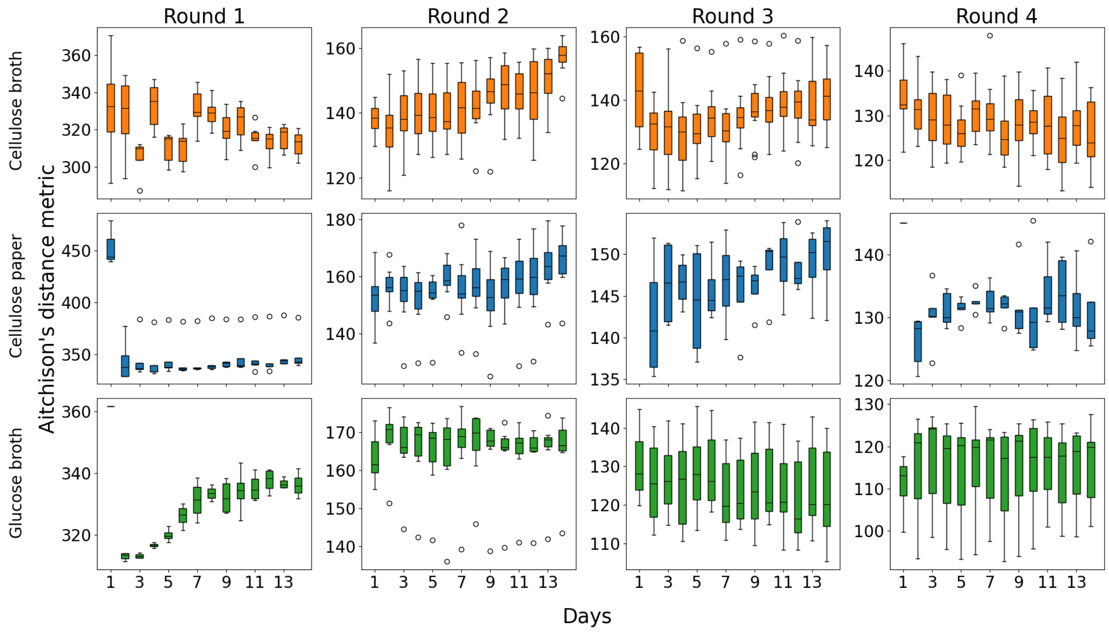

Figure S11: Aitchison's distance metric: The beta diversity metric, Aitchison's metric, which takes into account the compositional nature of the data is plotted on the y axis for the replicates. The x axis shows the days of sampling. Each column shows the rounds of transfer. The top row shows these data for communities in cellulose broth, the middle row for communities on paper, and the bottom row for communities on glucose. Each boxplot represents values across 5 replicate communities in each environment, where the boxes extend from the first to the third quartile, solid line represents the median, and the whiskers extend from the box to the farthest data point lying within 1.5 times the inter-quartile range.

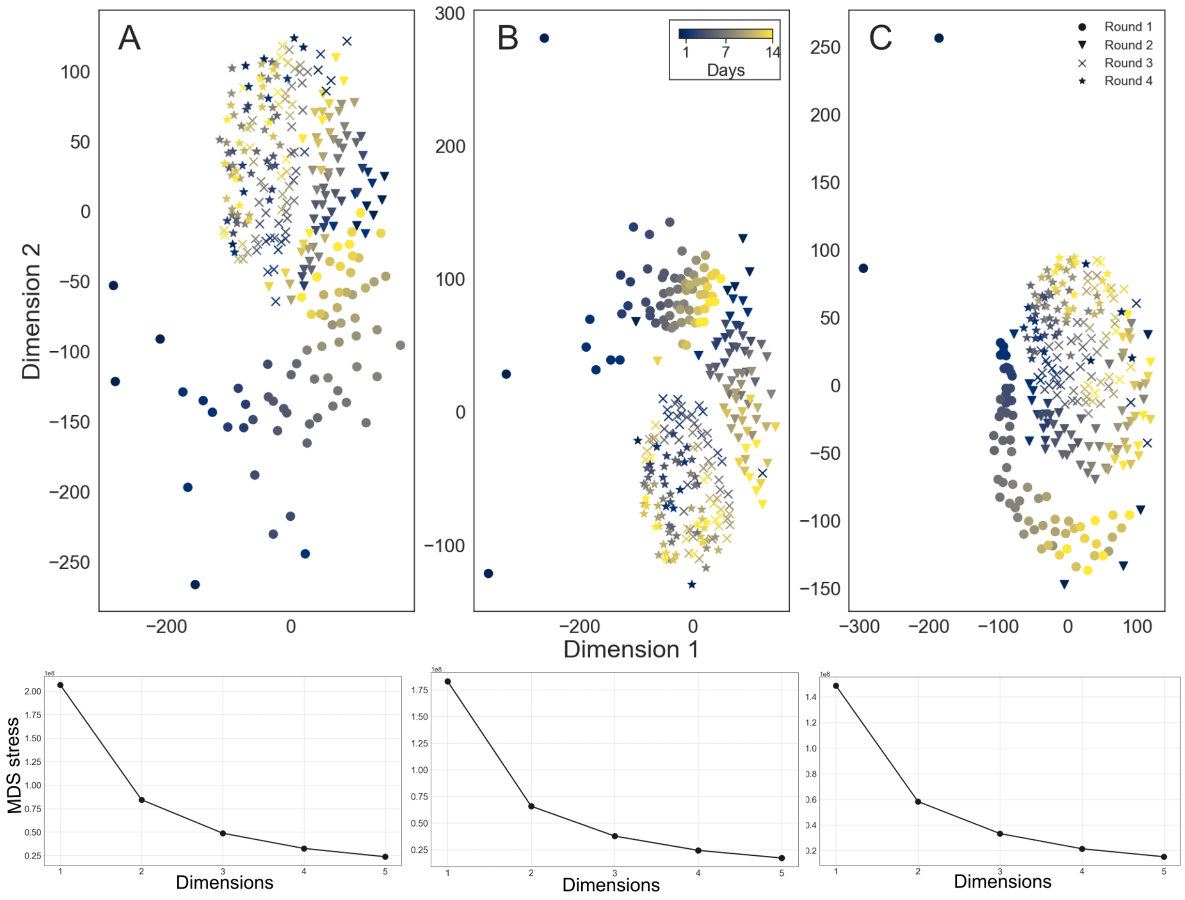

Figure S12: Embedding Aitchison's distances: The Aitchison's distance between all samples were embedded in two dimensions for each environment. **A** for cellulose broth **B** for paper and **C** for glucose. The bottom panels show the stress of the MDS embedding in each environment.

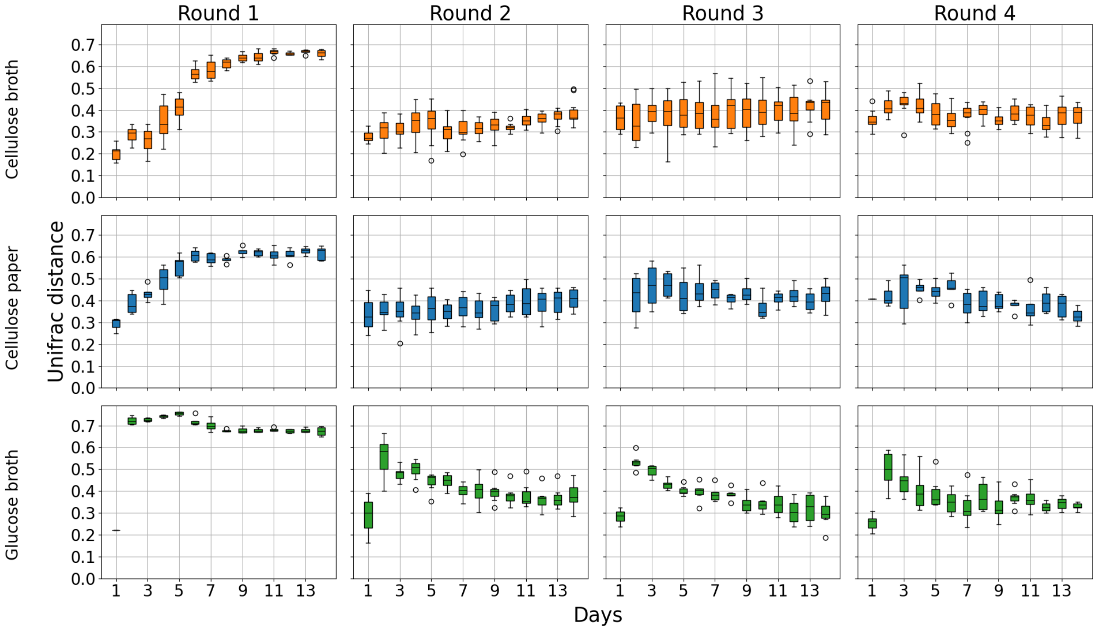

Figure S13: Unifrac distances across days: The beta diversity metric, Unifrac, which takes into account the phylogenetic information of the samples is plotted on the y axis for the replicates. The x axis shows the days of sampling. Each column shows the rounds of transfer. The top row shows these data for communities in cellulose broth, the middle row for communities on paper, and the bottom row for communities on glucose. Each boxplot represents values across 5 replicate communities in each environment, where the boxes extend from the first to the third quartile, solid line represents the median, and the whiskers extend from the box to the farthest data point lying within 1.5 times the inter-quartile range.

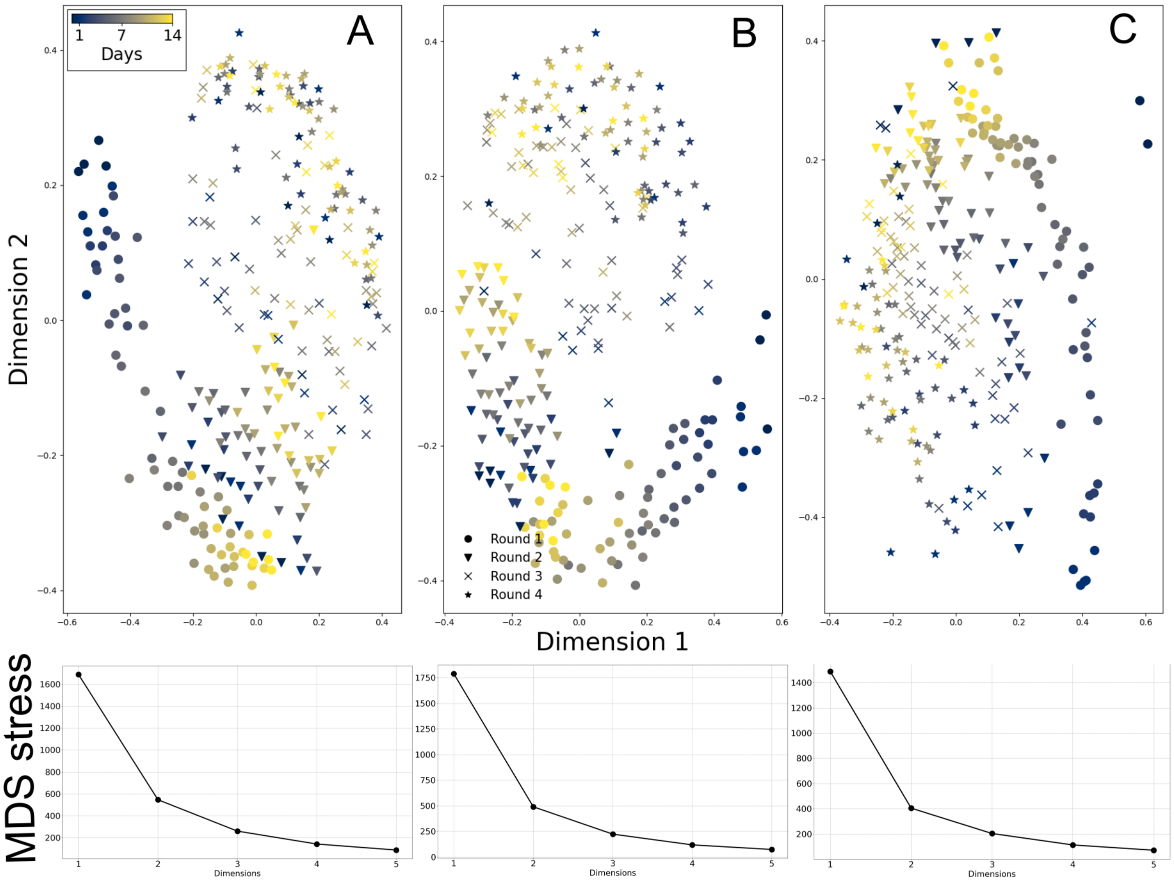

Figure S14: Embedding Unifrac distances:.The Unifrac distance between all samples were embedded in two dimensions for each environment. **A** for cellulose broth **B** for paper and **C** for glucose. The bottom panels show the stress of the MDS embedding in each environment.

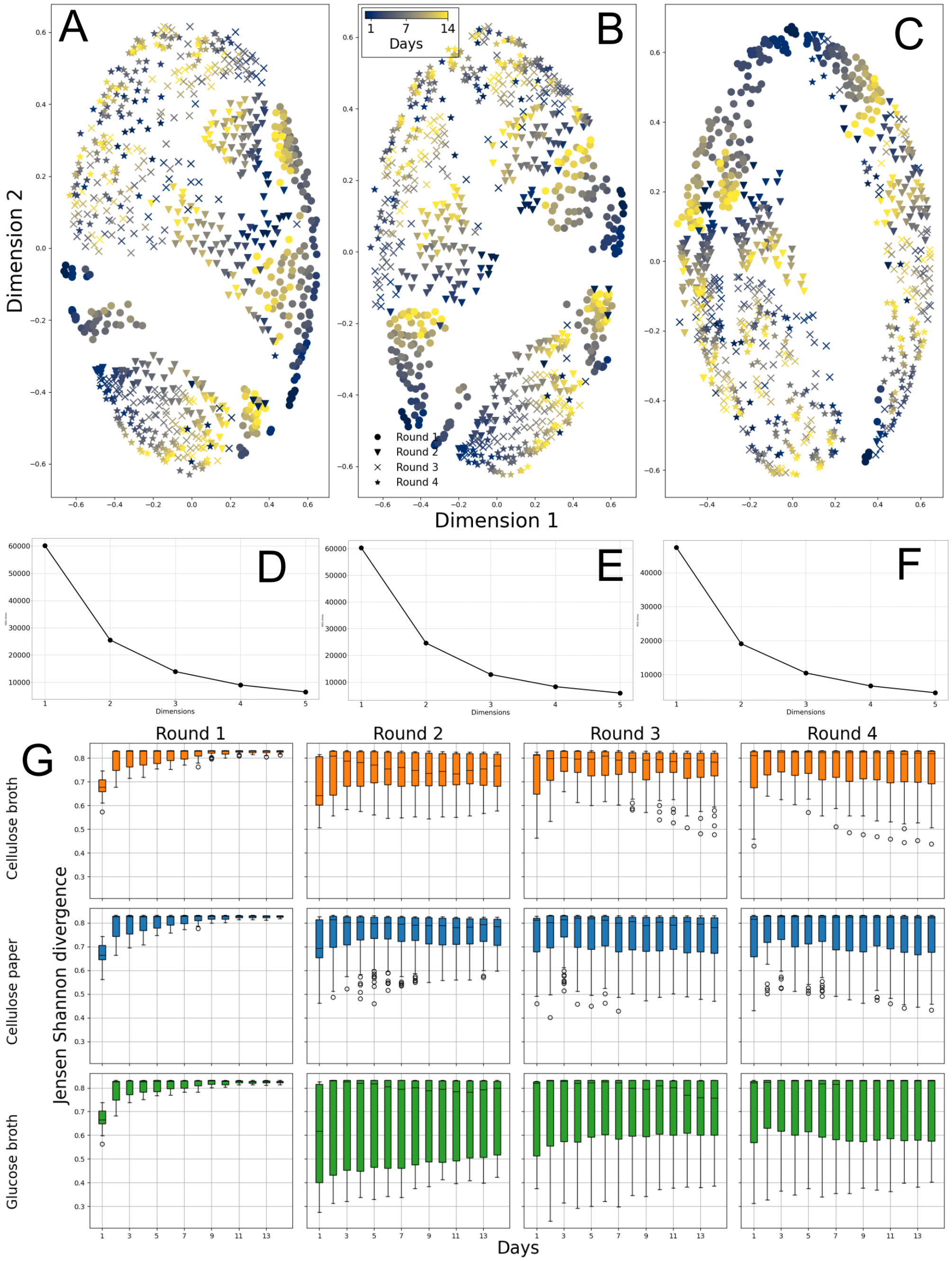

Figure S15: JSD for only low abundance taxa: The JSD was computed for the low abundance ASVs as described in methods. The pairwise distances were embedded in two dimensions for each environment. **A** for cellulose broth **B** for paper and **C** for glucose. **D-F** show the stress of the MDS embedding in each environment. **G** shows the distances between replicates; the top row for cellulose broth, the middle row for cellulose paper and the bottom row for glucose broth. Each boxplot represents values across 5 replicate communities in each environment, where the boxes extend from the first to the third quartile, solid line represents the median, and the whiskers extend from the box to the farthest data point lying within 1.5 times the inter-quartile range.

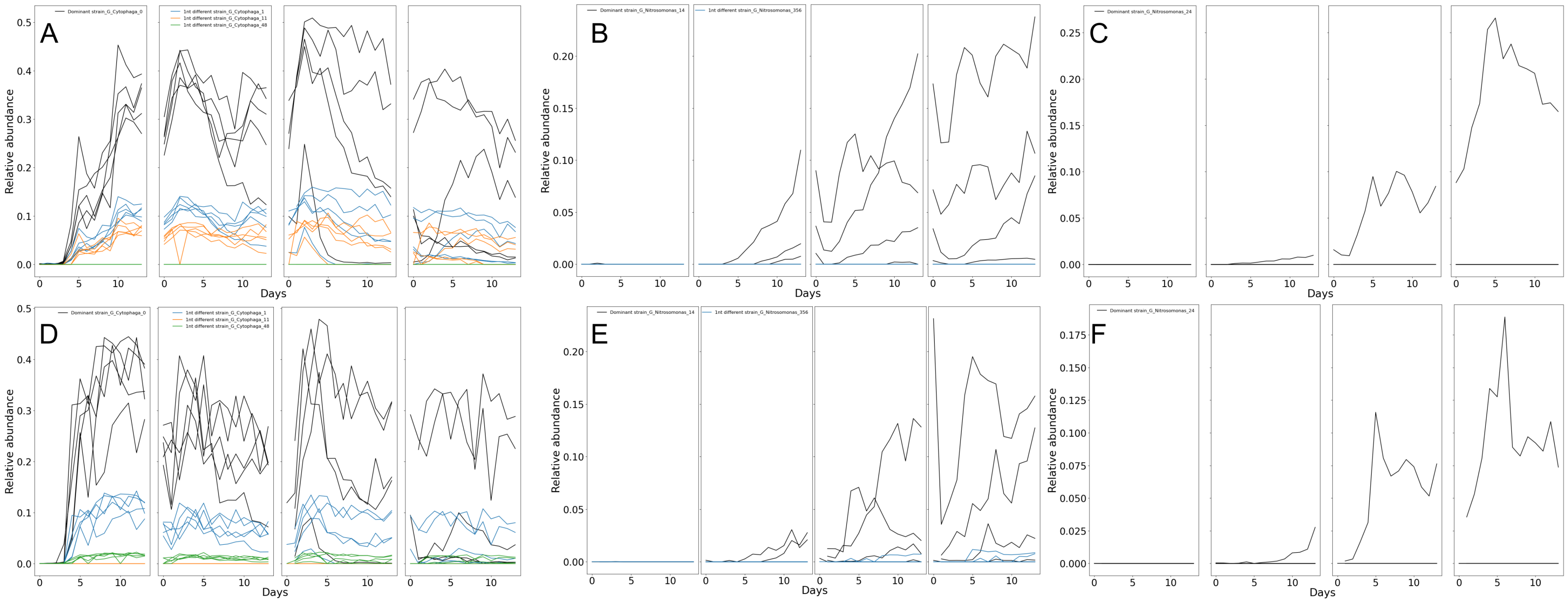

Figure S16: ASVs with 1 nucleotide differences in the cellulose environment: The relative abundance of the dominant ASV is plotted in black across all 14 days of all 4 rounds. In other colors, relative abundance of ASVs that are 1 nucleotide different from the dominant ASV are plotted **A-C** in the cellulose broth, **D-F** on paper. The different curves represent abundances in different replicates.

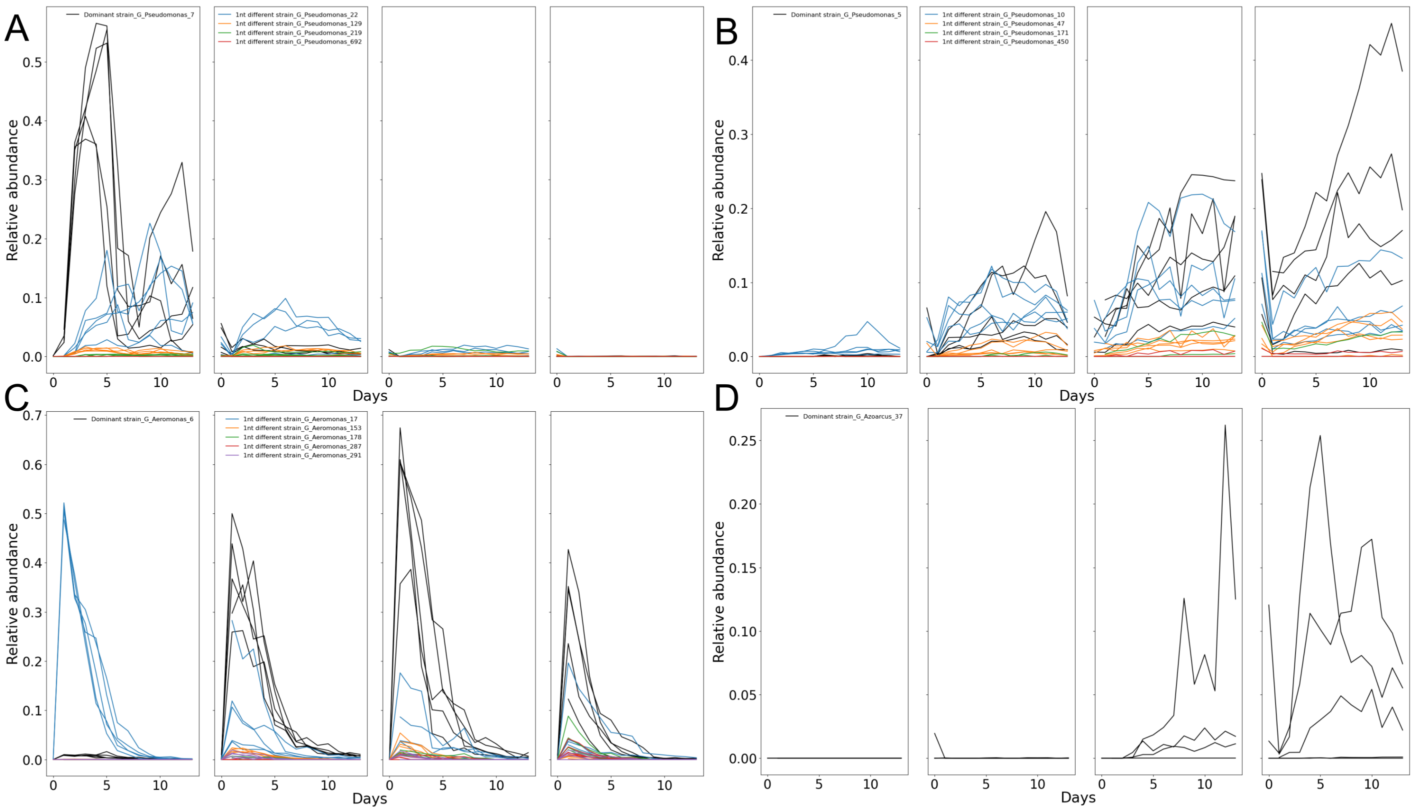

Figure S17: ASVs with 1 nucleotide differences in the glucose environment: The relative abundance of the dominant ASV is plotted in black across all 14 days of all 4 rounds. In other colors, relative abundance of ASVs that are 1 nucleotide different from the dominant ASV are plotted for **A** Psedumonas 7, **B** Pseudmonas 5, **C** Aeromonas 6 and **D** Azoarcus 37. The different curves represent the abundances in different replicates.

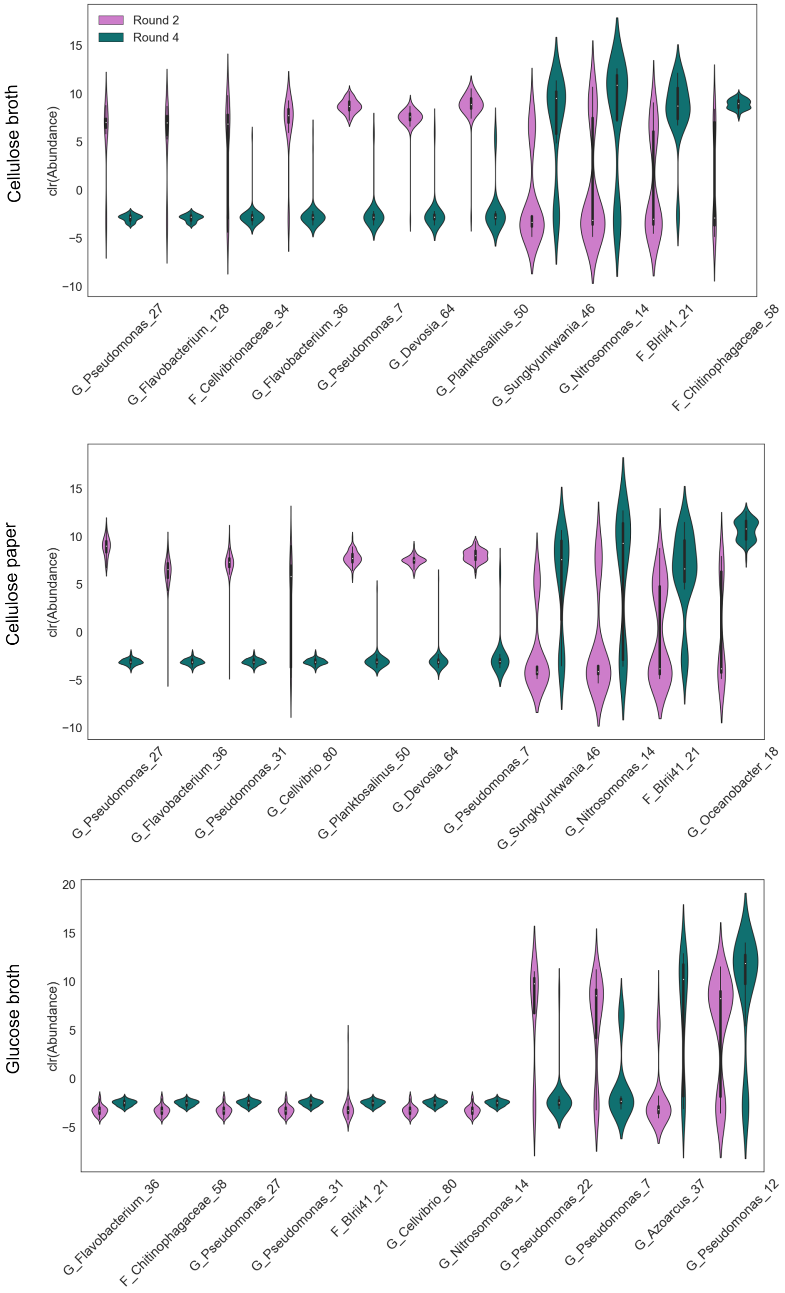

Figure S18: ASVs causing displacement of communities along PC2 between Round 2 and Round 4: The contribution of the ASVs to the displacement of communities between rounds 2 and 4 were calculated the top contributors were chosen, as described in Methods. The log transformed relative abundances in rounds 2 and 4 for these top contributors are plotted for the cellulose broth environment in the top panel, the cellulose paper environment for the middle panel and the glucose broth environment for the bottom panel. The violin plots represent the distributions across the 5 technical replicates.

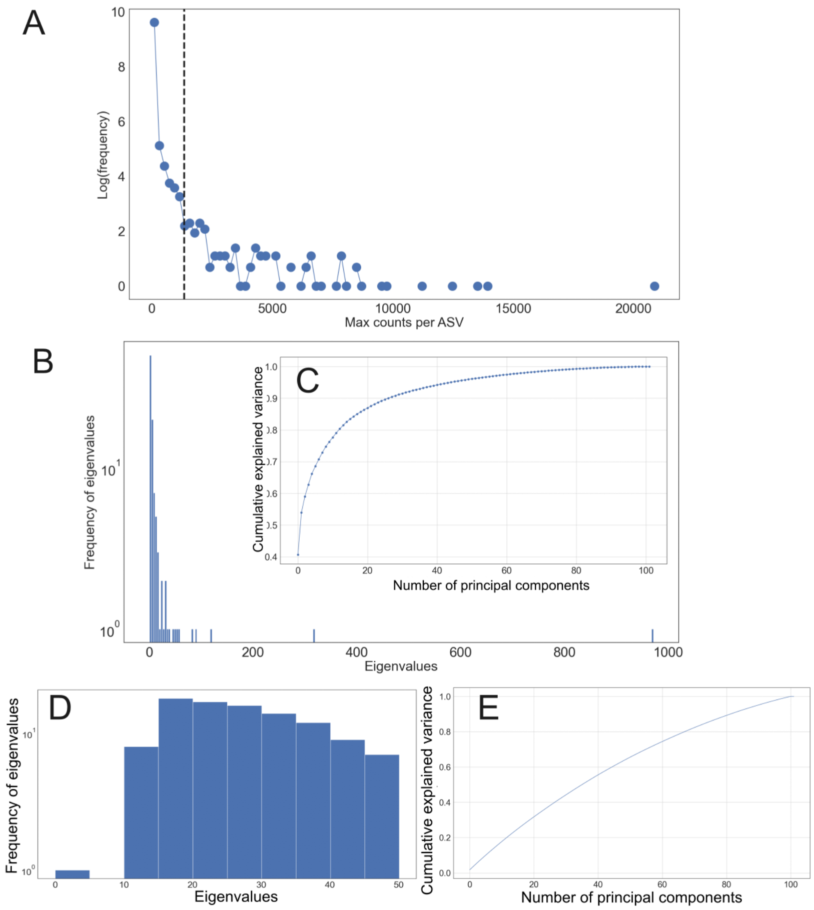

Figure S19: Thresholding and eigenvalues of thresholded data: For the PCA on 16S sequence data, the thresholding was performed as in Methods. **A** shows the logarithm of the frequency of maximum counts an ASV has across all sample. The dashed line shows the cutoff for thresholding. **B** The eigenvalue distribution for the thresholded data. **C** the cumulative explained variance **D** The eigenvalue distribution and **E** cumulative explained variance of the shuffled data.

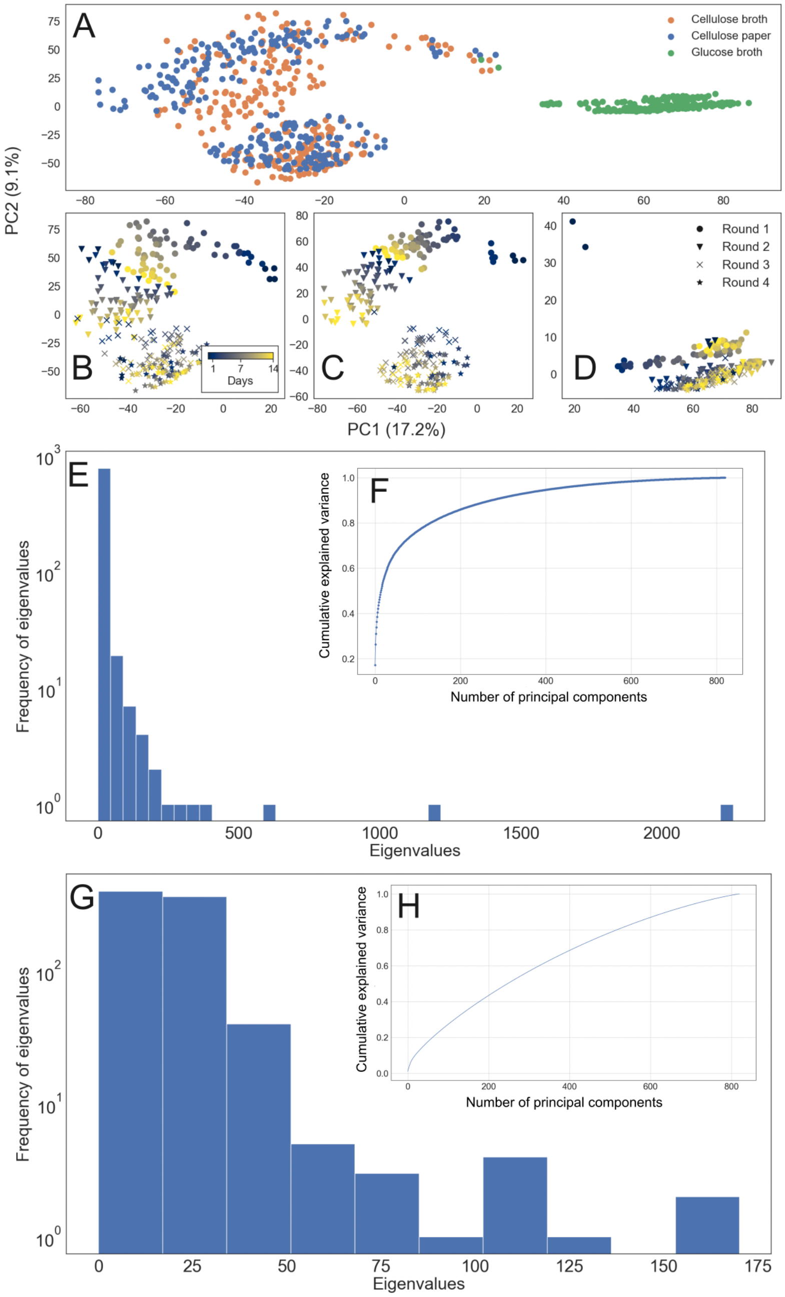

Figure S20: PCA with the full data set: **A** PCA embedded in two dimensions on the full 16S data. The colors represent the environment. **B-D** The same embedding as in A, but separated by the environments: cellulose broth, paper and glucose respectively. The colors represent the 14 days and the markers represent the 4 rounds of transfer. **E** Eigenvalue distribution **F** Cumulative explained variance **G** Eigenvalue distribution and **H** cumulative explained variance of shuffled data.

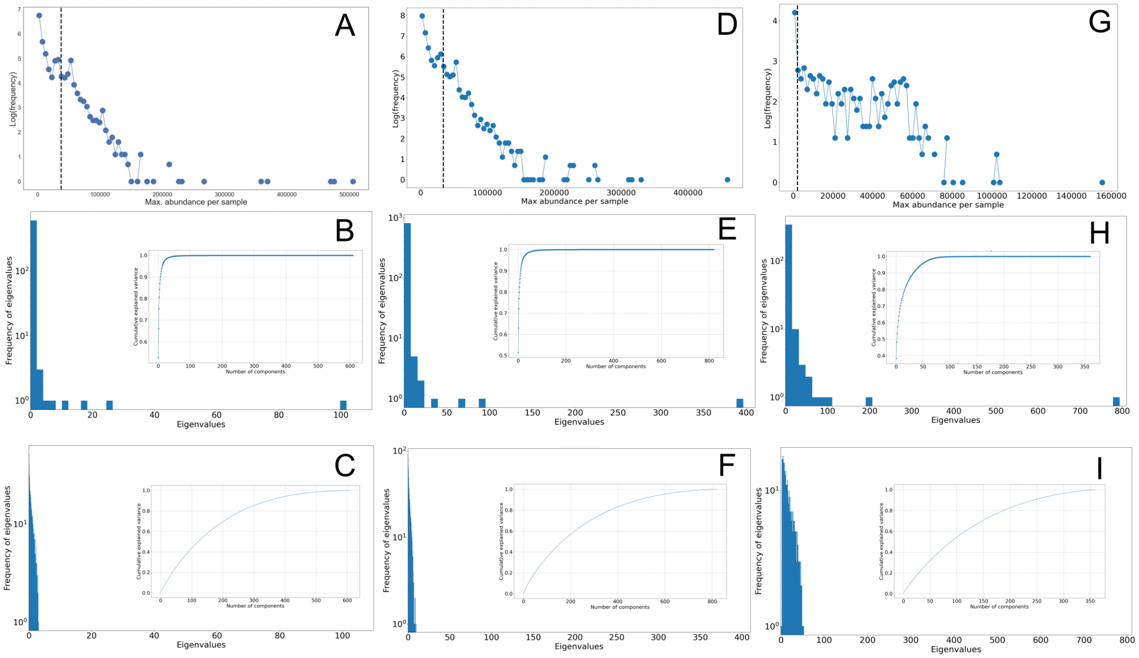

Figure S21: Details of PCA on PICRUSt2 output: PICRUSt2 provides 3 outputs - abundances of EC number, KO and pathways. The logarithm of the frequency of the maximum abundance across all samples, the eigenvalue distribution with an inset of the cumulative explained variance, and the eigenvalue distribution and cumulative explained variance of the shuffled data are shown for **A-C** cellulose broth environment **D-F** cellulose paper and **G-I** glucose.

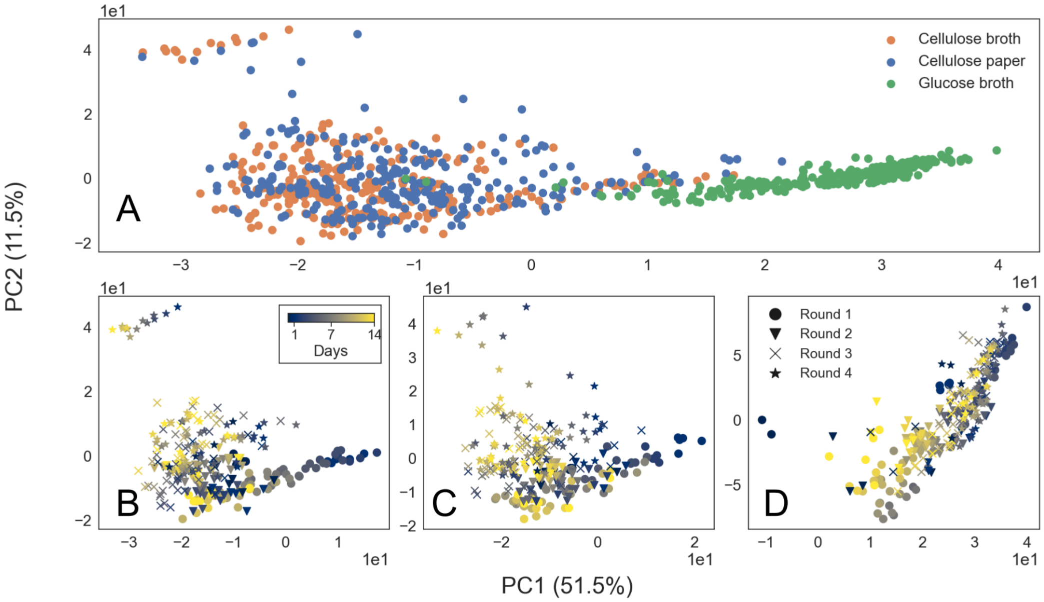

Figure S22: PCA on Kegg Ortholog abundance output of PICRUSt2: PCA was performed on the KO abundances as described in methods. **A** shows the 2 dimensional embedding of the sample, with the colors corresponding tot he environment. **B-D** shows the same embedding as in A, but separated by the environments: cellulose broth, paper and glucose respectively. The colors show the 14 days of sampling within each round, and the markers represent the rounds of transfer.

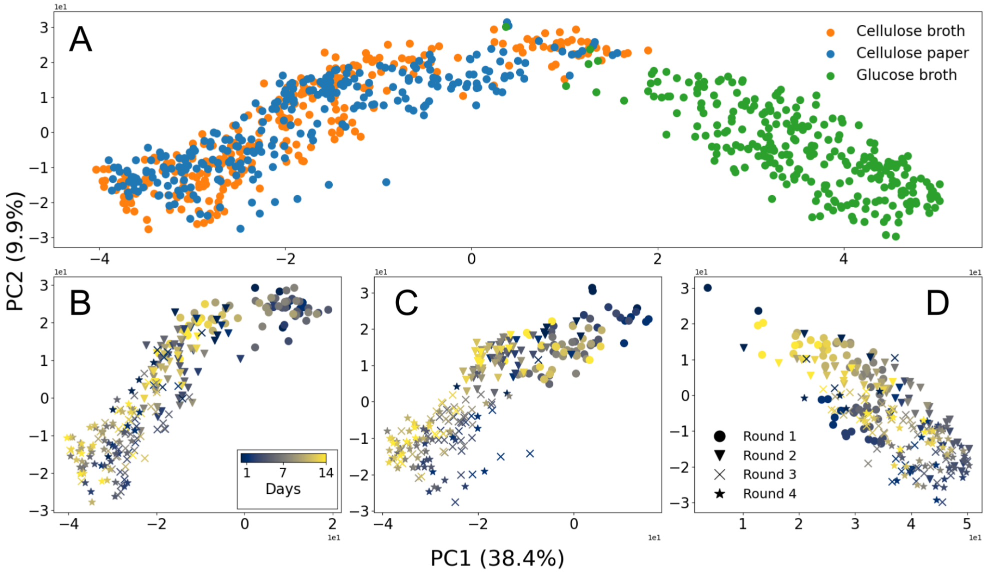

Figure S23: PCA on pathway abundance output of PICRUSt2: PCA was performed on the pathway abundances as described in methods. **A** shows the 2 dimensional embedding of the sample, with the colors corresponding to the environment. **B-D** shows the same embedding as in A, but separated by the environments: cellulose broth, paper and glucose respectively. The colors show the 14 days of sampling within each round, and the markers represent the rounds of transfer.

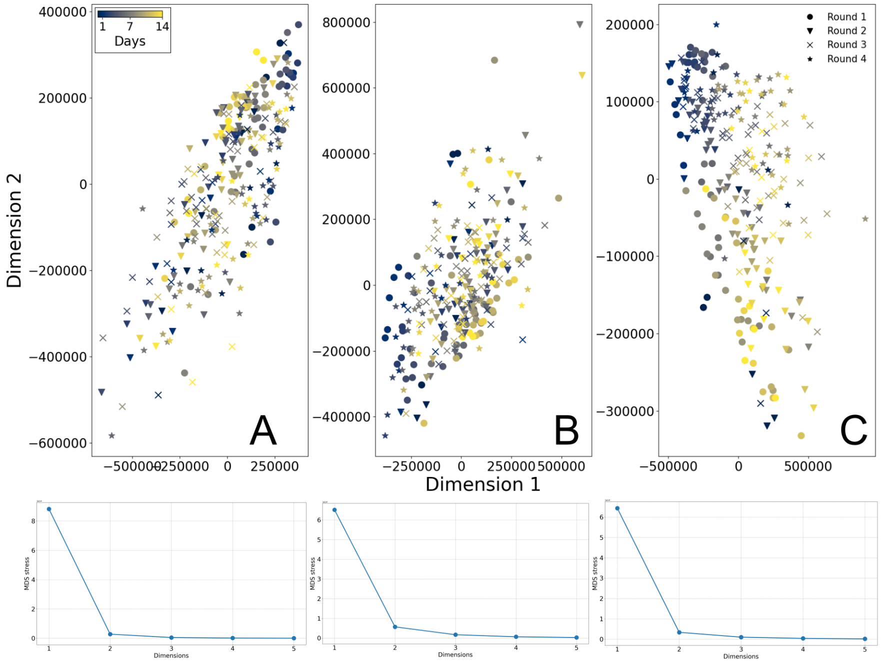

Figure S24: Embedding Euclidean distances of EC number composition: The Euclidean distances between communities were computed based on the EC number composition, as described in methods. The pairwise distances were embedded in two dimensions using multidimensional embedding for each environment **A** cellulose broth **B** cellulose paper and **C** glucose. The bottom panels show the stress of the embedding.

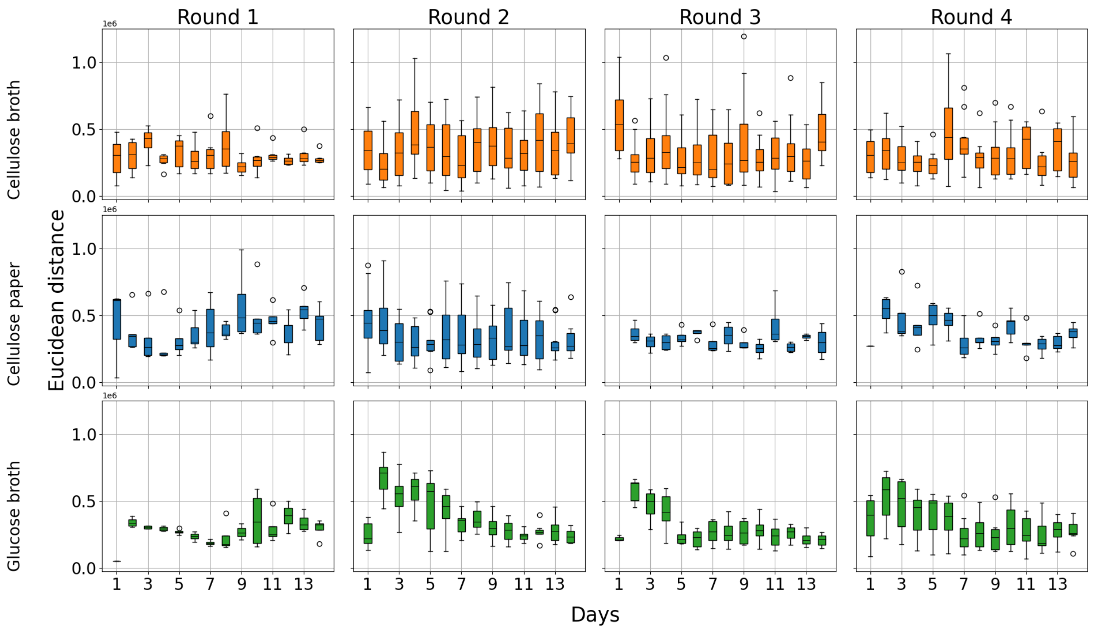

Figure S25: Euclidean distances of EC composition across replicates: The Euclidean distances between replicates based on EC composition were calculated. These are shown on the y axis. The x axis shows the days of sampling and the columns show the 4 rounds of transfer. The top row is for cellulose broth, the middle row for cellulose paper and bottom row for glucose. Each boxplot represents values across 5 replicate communities in each environment, where the boxes extend from the first to the third quartile, solid line represents the median, and the whiskers extend from the box to the farthest data point lying within 1.5 times the inter-quartile range.

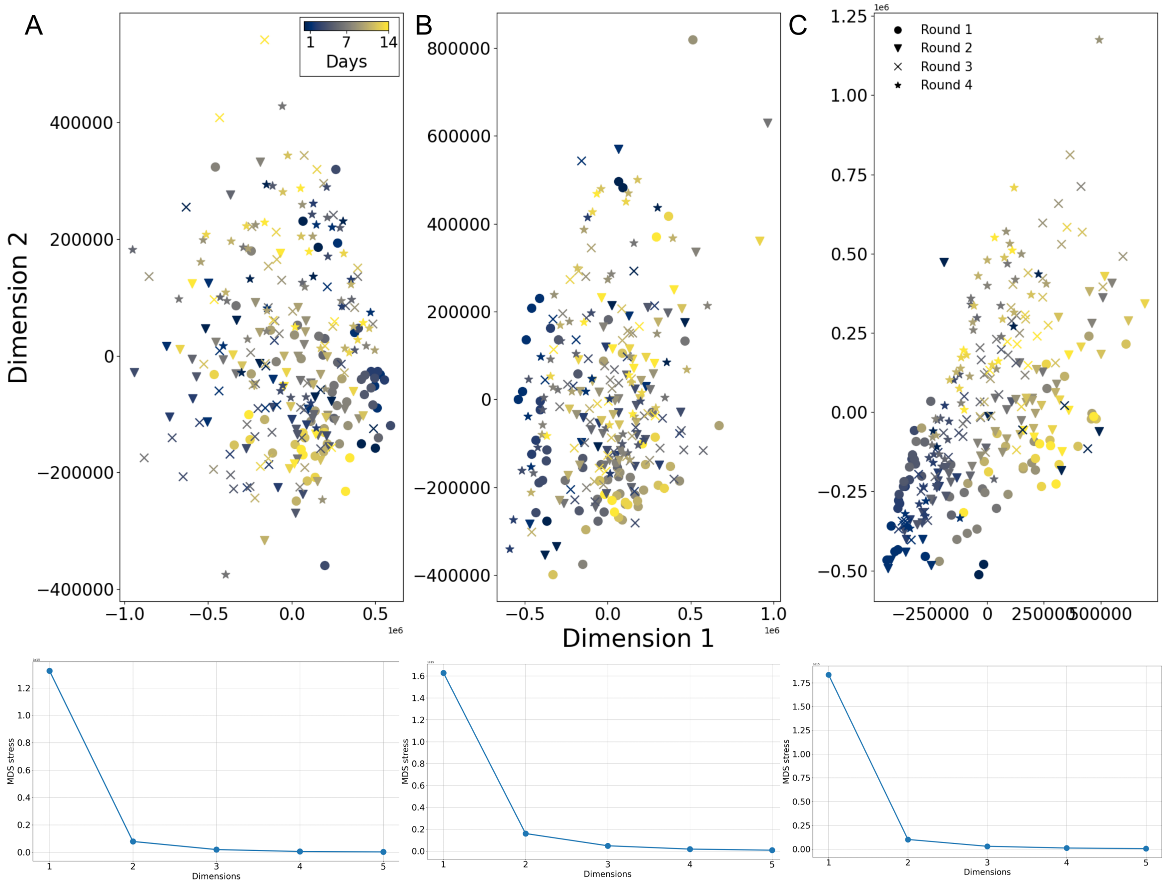

Figure S26: Embedding Euclidean distances of KO composition: The Euclidean distances between communities were computed based on the KO composition, as described in methods. The pairwise distances were embedded in two dimensions using multidimensional embedding for each environment **A** cellulose broth **B** cellulose paper and **C** glucose. The bottom panels show the stress of the embedding.

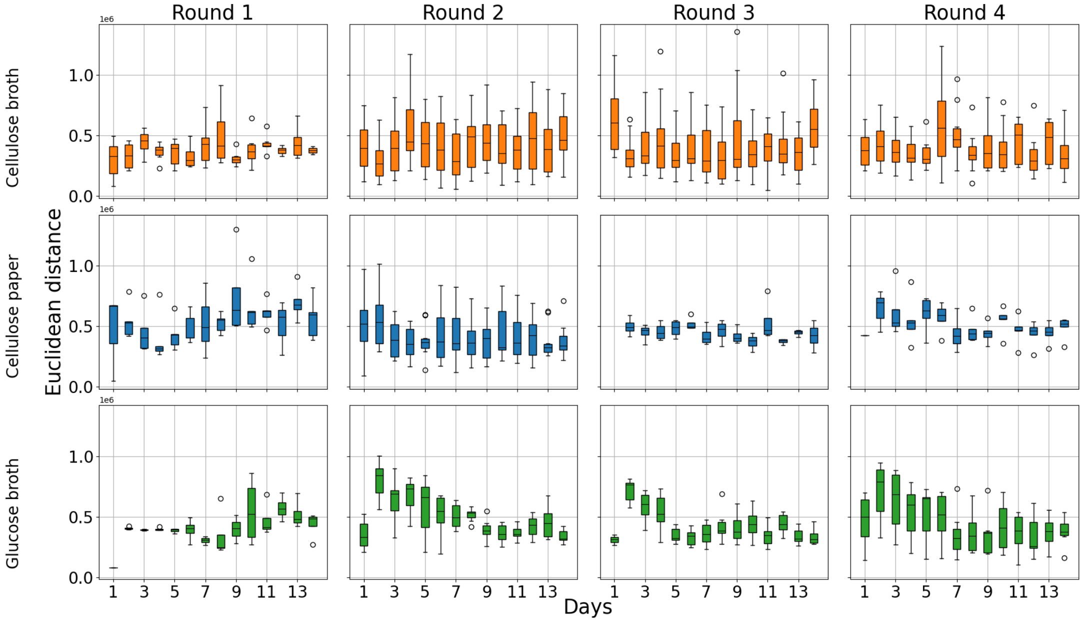

Figure S27: Euclidean distances of KO composition across replicates: The Euclidean distances between replicates based on KO composition were calculated. These are shown on the y axis. The x axis shows the days of sampling and the columns show the 4 rounds of transfer. The top row is for cellulose broth, the middle row for cellulose paper and bottom row for glucose. Each boxplot represents values across 5 replicate communities in each environment, where the boxes extend from the first to the third quartile, solid line represents the median, and the whiskers extend from the box to the farthest data point lying within 1.5 times the inter-quartile range.

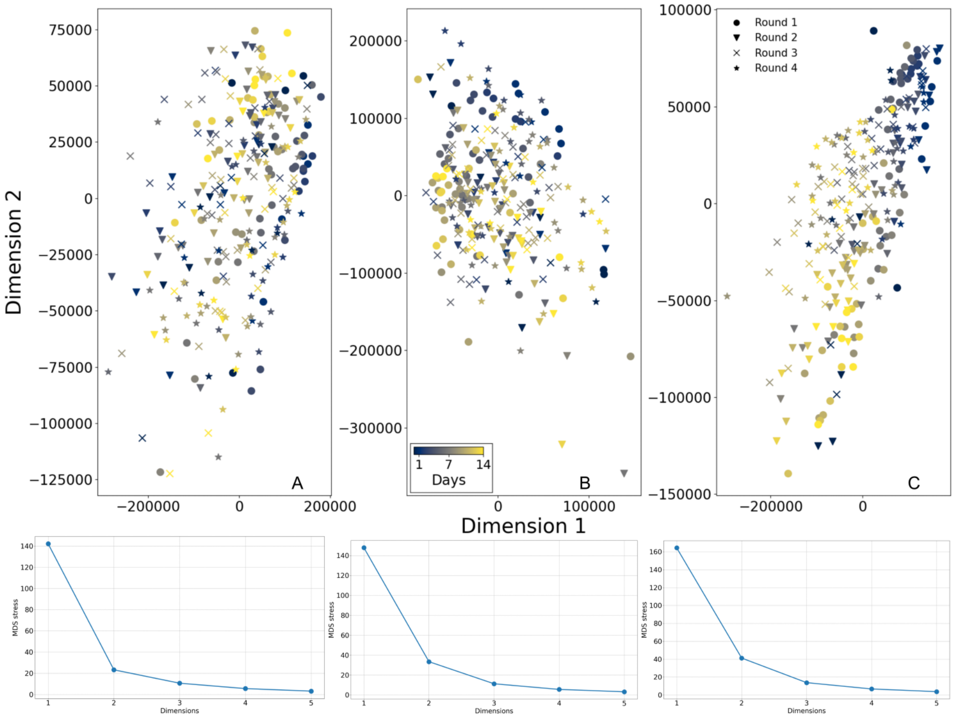

Figure S28: Embedding Euclidean distances of pathways composition: The Euclidean distances between communities were computed based on the pathways composition, as described in methods. The pairwise distances were embedded in two dimensions using multidimensional embedding for each environment **A** cellulose broth **B** cellulose paper and **C** glucose. The bottom panels show the stress of the embedding.

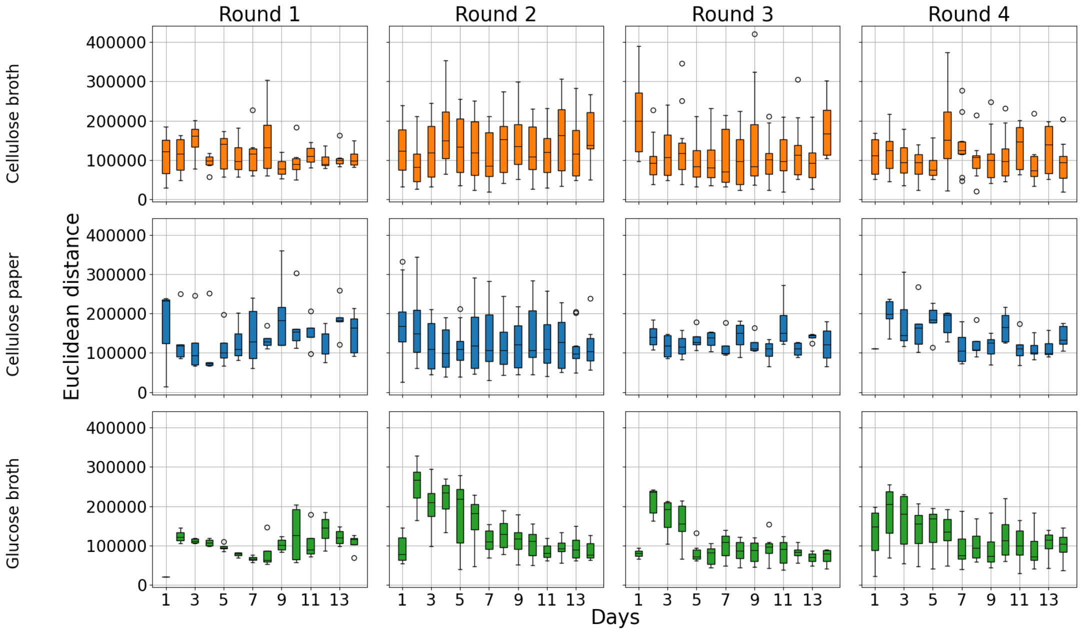

Figure S29: Euclidean distances of pathways composition across replicates: The Euclidean distances between replicates based on pathways composition were calculated. These are shown on the y axis. The x axis shows the days of sampling and the columns show the 4 rounds of transfer. The top row is for cellulose broth, the middle row for cellulose paper and bottom row for glucose.Each boxplot represents values across 5 replicate communities in each environment, where the boxes extend from the first to the third quartile, solid line represents the median, and the whiskers extend from the box to the farthest data point lying within 1.5 times the inter-quartile range.

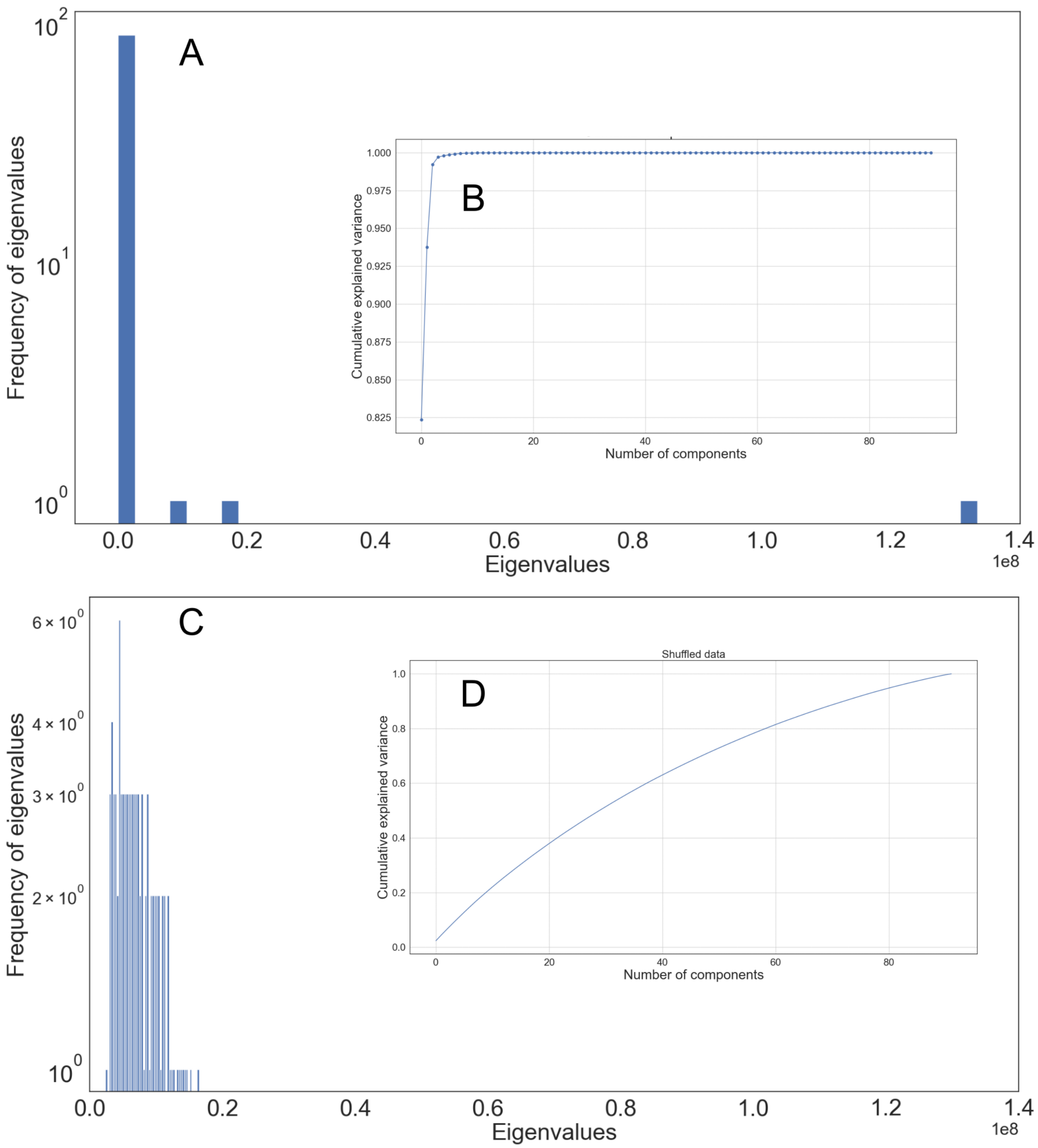

Figure S30: Eigenvalues PCA on FAPROTAX output: **A** shows the eigenvalues distribution of the FAPROTAX output **B** shows the cumulative explained variance. **C-D** show the eigenvalue distribution and cumulative explained variance for the shuffled data.

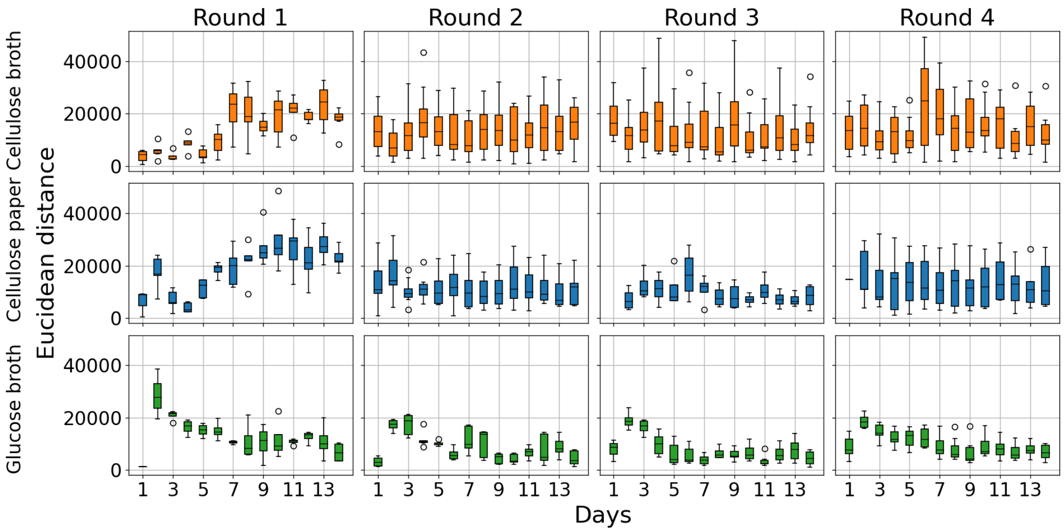

Figure S31: Euclidean distances of functional composition across replicates: The Euclidean distances between replicates based on the functional composition output of FAPROTAX were calculated. These are shown on the y axis. The x axis shows the days of sampling and the columns show the 4 rounds of transfer. The top row is for cellulose broth, the middle row for cellulose paper and bottom row for glucose.

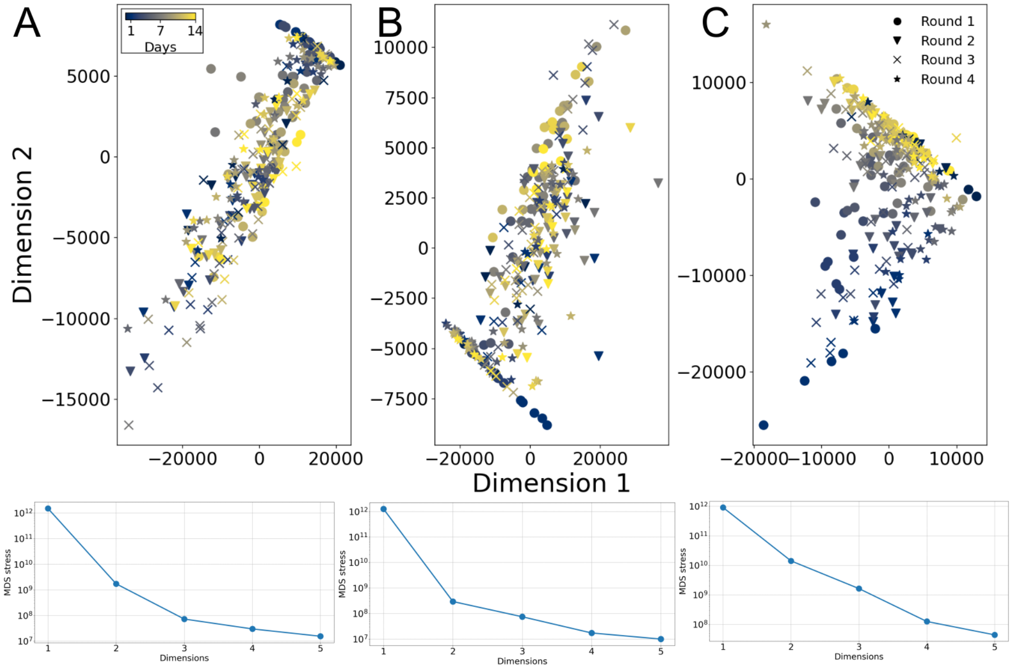

Figure S32: Embedding Euclidean distances of functional composition provided by FAPROTAX: The Euclidean distances between communities were computed based on the functional composition output of FAPROTAX, as described in methods. The pairwise distances were embedded in two dimensions using multidimensional embedding for each environment **A** cellulose broth **B** cellulose paper and **C** glucose. The bottom panels show the stress of the embedding. Each boxplot represents values across 5 replicate communities in each environment, where the boxes extend from the first to the third quartile, solid line represents the median, and the whiskers extend from the box to the farthest data point lying within 1.5 times the inter-quartile range.

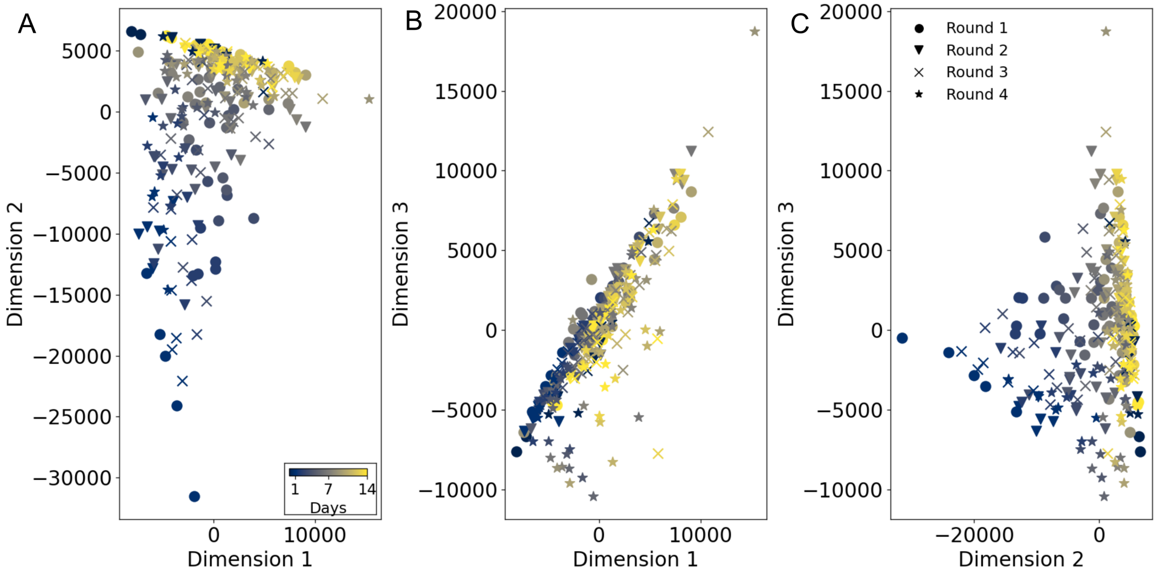

Figure S33: 3 dimensional embedding of euclidean distances of functional composition provided by FAPROTAX of glucose communities: The glucose communities were embedded in 3 dimensions to look for any additional clustering of the samples. **A-C** show the embeddings in Dimensions 1 and 2, 1 and 3 and 2 and 3 respectively.

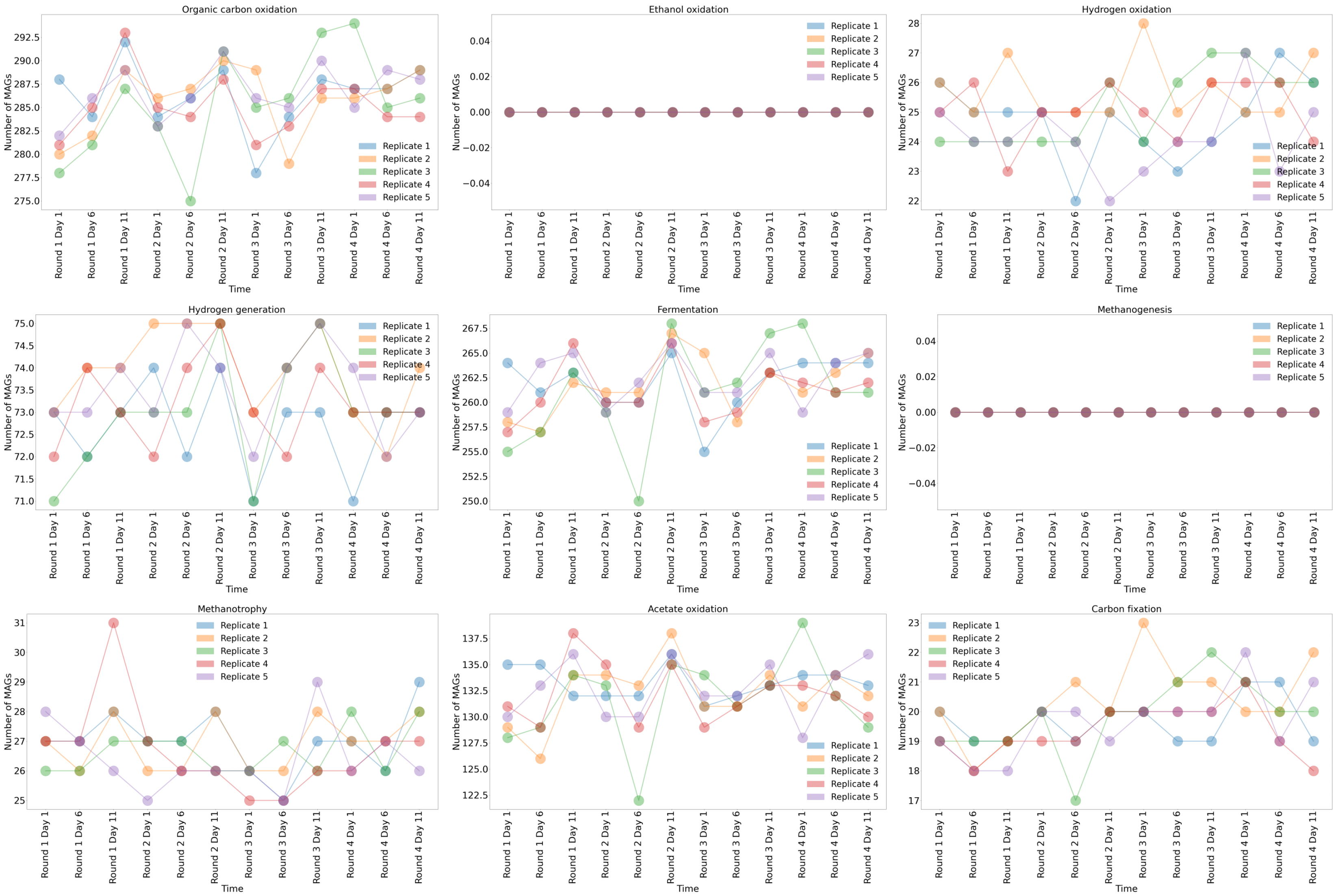

Figure S34: Genomes performing the carbon cycle based on metagenomic analyses: Each panel shows the coverage on y-axis (the fraction of genomes in the community that can perform the function shown in the panel title) of the pathway in the carbon cycle, with time on the x-axis.

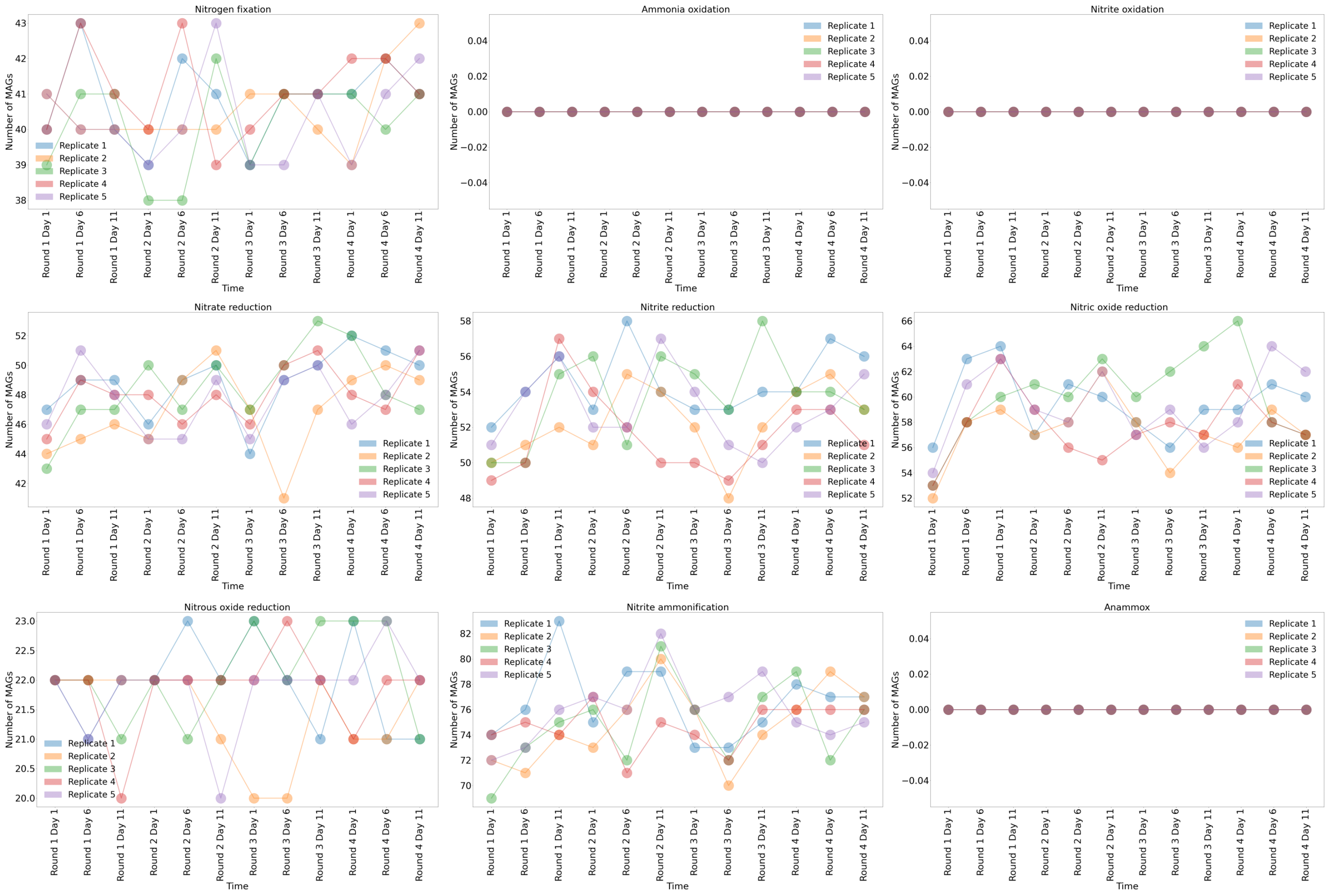

Figure S35: Genomes performing the nitrogen cycle based on metagenomic analyses: Each panel shows the coverage on y-axis (the fraction of genomes in the community that can perform the function shown in the panel title) of the pathway in the nitrogen cycle, with time on the x-axis.

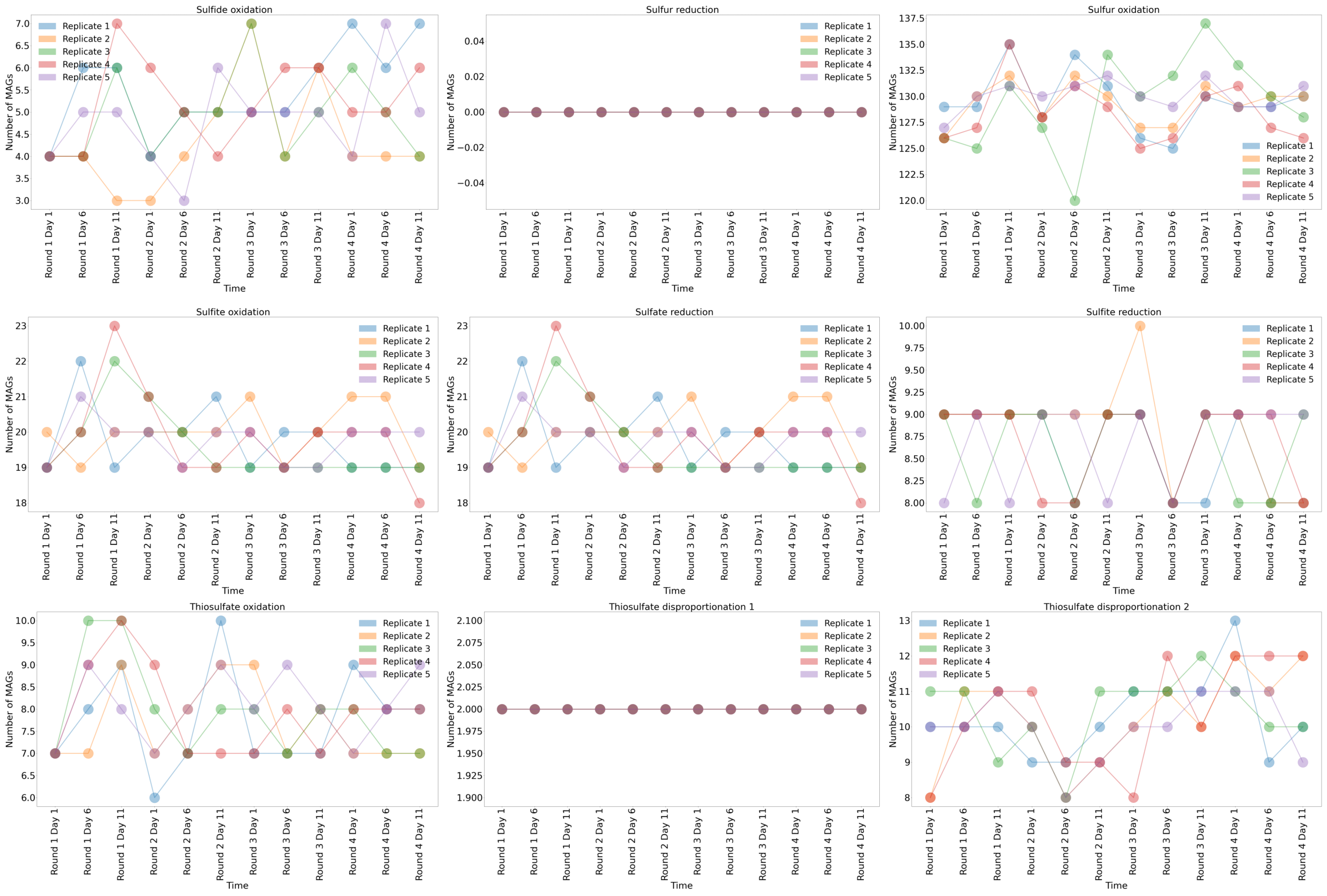

Figure S36: Genomes performing the sulphur cycle based on metagenomic analyses: Each panel shows the coverage on y-axis (the fraction of genomes in the community that can perform the function shown in the panel title) of the pathway in the sulphur cycle, with time on the x-axis.

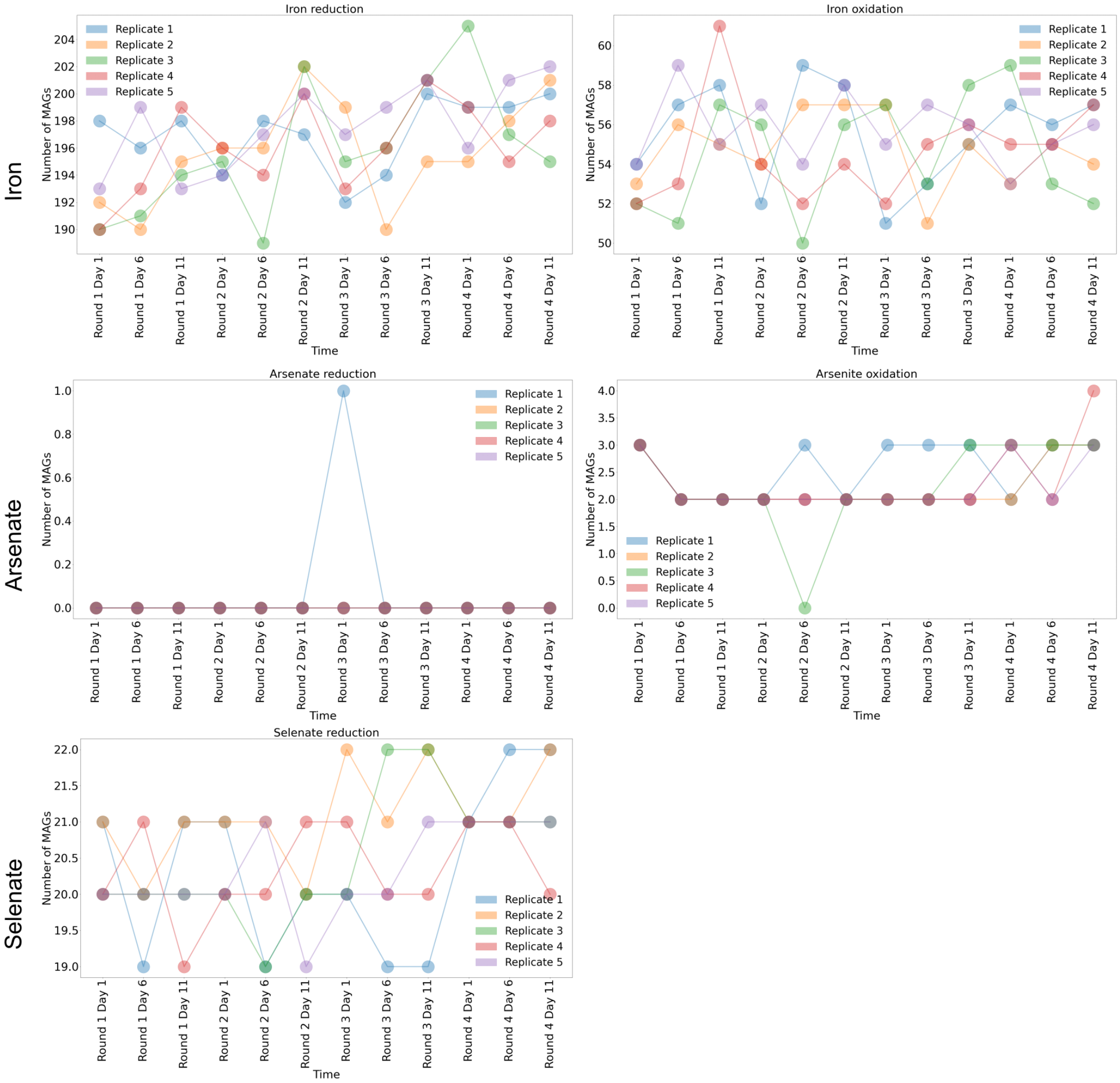

Figure S37: Genomes performing other elemental metabolism based on metagenomic analyses: Each panel shows the coverage on y-axis (the fraction of genomes in the community that can perform the function shown in the panel title) of the pathway, with time on the x-axis. The top panel shows iron metabolism, the middle panel arsenate and the bottom panel selenate.

High in cellulose broth		High in glucose	
Function indicated	Enzyme	Function indicated	Enzyme
DNA replication	DNA-directed DNA polymerase	Electron transfer	glutathione transferase
DNA replication	DNA helicase	Amino acid salvage	ABC type polar amino-acid transporter
Electron transport chain	NADH:ubiquinone reductase	Salvage	sarcosine oxidase (formaldehyde-forming)
Aerobic respiration	cytochrome-c oxidase	Cross feeding	formate dehydrogenase
DNA cleavage	type I site-specific deoxyribonuclease	Salvage	xanthine dehydrogenase
Cellulolysis	beta-glucosidase	Salvage	taurine dioxygenase
Aerobic respiration	methylmalonyl-CoA mutase	lipid metabolism	acetyl-CoA C-acetyltransferase
Protein folding	peptidylprolyl isomerase	lipid metabolism	enoyl-CoA hydratase
	site-specific DNA-methyl-transferase (adenine-specific)	Amino acid biosynthesis	glutamine synthetase
Aerobic respiration	cytochrome-c peroxidase		catalase
Catabolism of amino acids	branched chain amino-acid transaminase	Nitrogen cycling	amidase
Electron transfer	thioredoxin-disulfide reductase	Nitrogen cycling	urease
Sulphur cycle	cysteine desulfurase	Cross feeding	hippurate hydrolase
Cross feeding	alpha-L-fucosidase	TCA cycle	phosphoglycolate phosphatase
Salvage	alanine dehydrogenase	Nitrogen cycling	nitronate monooxygenase

Table S1: Enzymes that have significantly higher abundances in the two environments. Derived from 16S data using PICRUSt2, the enzymes whose abundance was higher in CB compared to glucose are listed in the second column and their broad role in the first, and the enzymes whose abundance was higher in the glucose-supported environment compared to the CB environment are listed in the fourth column and their broad role in the third.

High in cellulose broth		High in glucose	
Function indicated	KO	Function indicated	KO
Transport	putative ABC transport system	Amino acid salvage	polar amino acid transport system substrate-binding protein
Transport	ABC-2 type transport system ATP-binding protein	Amino acid salvage	polar amino acid transport system permease protein
Regulation	LacI family transcriptional regulator	Sensing	methyl-accepting chemotaxis protein
Stress response	RNA polymerase sigma-70 factor (rpoE)	Electron transfer	glutathione S-transferase
Transport	two-component system, LytTR family, response regulator	Salvage	putrescine transport system substrate-binding protein
Transport	ATP-binding cassette	Lipid metabolism	acyl-ACP dehydrogenase
Transport	ABC-2 type transport system permease protein	Sensing	transmembrane sensor
Transport	putative ABC transport system ATP-binding protein	Amino acid salvage	polar amino acid transport system ATP-binding protein
Transport	hydrophobic /amphiphilic exporter-1	Salvage	putative spermidine/putrescine transport system substrate-binding protein
Aerobic rspiration	cytochrome c oxidase subunit III	Salvage	glycine cleavage system transcriptional activator
Cellulolysis	beta-glucosidase	Salvage	glycine betaine/proline transport system substrate-binding protein
Aerobic respiration	cytochrome c peroxidase	Sensing	outer-membrane receptor for ferric coprogen and ferric-rhodotorulic acid
Amino acid production	branched-chain amino acid aminotransferase	Biofilm formation	type VI secretion system secreted protein
Electron transfer	thioredoxin reductase (NADPH)	Transport	D-methionine transport system substrate-binding protein
Salvage	alanine dehydrogenase	Salvage	formate dehydrogenase major subunit

Table S2: Kegg-orthologs (KOs) that have significantly higher abundances in the two environments. Derived from 16S data using PICRUSt2, the KOs whose abundance was higher in CB compared to glucose are listed in the second column and their broad role in the first, and the KOs whose abundance was higher in the glucose-supported environment compared to the CB environment are listed in the fourth column and their broad role in the third.

High in cellulose broth		High in glucose	
Function indicated	Pathway	Function indicated	Pathway
Amino acid biosynthesis	L-valine biosynthesis	TCA cycle	TCA cycle VII
Amino acid biosynthesis	L-isoleucine biosynthesis I	TCA cycle	TCA cycle VI
Amino acid biosynthesis	L-isoleucine biosynthesis II	Salvage	ornithine degradation
Salvage	pyruvate fermentation to propionate	Amino acid biosynthesis	L-isoleucine biosynthesis IV
Aerobic respiration	aerobic respiration I (cytochrome c)	Lipid biosynthesis	stearate biosynthesis II
Cell wall biosynthesis	dTDP-N-acetylthomosamine biosynthesis	vitamin biosynthesis	pyridoxal 5'-phosphate biosynthesis and salvage
Salvage	superpathway of pyrimidine ribonucleosides salvage	Lipid biosynthesis	palmitoleate biosynthesis I
Amino acid biosynthesis	Branched chain amino acid synthesis	Amino acid biosynthesis	L-aspartate and L-aspargine biosynthesis
Salvage	pyrimidine deoxyribonucleosides salvage	Salvage	protocatechuate degradation II
Pectin decomposition	D-galacturonate degradation I	Lipid biosynthesis	mycolate biosynthesis
Salvage	adenine and adenosine salvage III	Lipid biosynthesis	oleate biosynthesis IV
Vitamin synthesis	NAD de novo biosynthesis II (from tryptophan)	Lipid biosynthesis	(5Z)-dodecenoate biosynthesis I
Cell wall biosynthesis	GDP-mannose-derived O-antigen building blocks biosynthesis	Lipid biosynthesis	palmitate biosynthesis II
	taxadiene biosynthesis (engineered)	Salvage	fatty acid salvage
Amino acid biosynthesis	L-isoleucine biosynthesis III	vitamin biosynthesis	adenosylcobalamin biosynthesis from adenosylcobinamide-GDP I

Table S3: Pathways that have significantly higher abundances in the two environments. Derived from 16S data using PICRUSt2, the pathways whose abundance was higher in CB compared to glucose are listed in the second column and their broad role in the first, and the pathways whose abundance was higher in the glucose-supported environment compared to the CB environment are listed in the fourth column and their broad role in the third.

High in cellulose broth	High in glucose
cellulolysis	nitrate reduction
aerobic chemoheterotrophy	fermentation
chemoheterotrophy	nitrogen respiration
ureolysis	nitrate respiration
phototrophy	human associated
photoheterotrophy	human pathogens all
	sulfate respiration
	respiration of sulfur compounds
	animal parasites or symbionts

Table S4: Functions that have significantly higher abundances in the two environments. Derived from 16S data using FAPROTAX, the functions whose abundance was higher in CB compared to glucose are listed in the first column, and the functions whose abundance was higher in the glucose-supported environment compared to the CB environment are listed in the second column.

High in the initial days		High in the final days	
Function indicated	Enzyme	Function indicated	Enzyme
TCA cyle	6-phospho-beta-glucosidase	DNA replication	DNA-directed DNA polymerase
Anaerobic respiration	formate C-acetyltransferase	Electron transfer	NADH:ubiquinone reductase (H+-translocating)
Anaerobic respiration	[formate-C-acetyltransferase] -activating enzyme	DNA replication	DNA 5'-3' helicase
Aerobic respiration	pyruvate synthase	Transport	histidine kinase
Lipid biosynthesis	glycerol dehydrogenase	Protein folding	peptidylprolyl isomerase
Salvage	PepB aminopeptidase	Aerobic respiration	cytochrome-c oxidase
Lipid biosynthesis	acyl-Kdo2-lipid IVA acyltransferase	Electron transfer	glutathione transferase
TCA cycle	malate dehydrogenase (oxaloacetate-decarboxylating)	Lipid metabolism	3-oxoacyl-[acyl -carrier-protein] reductase
TCA cycle	sugar-phosphatase	Amino acid biosynthesis	Glutamine synthetase
Electron transfer	hydrogenase (acceptor)	Lipid metabolism	acetyl-CoA C-acetyltransferase

Table S5: Enzymes that have significantly higher abundances in the early and later growth phases in the glucose environment. Derived from 16S data using PICRUSt2, the enzymes whose abundance was higher in the early growth phase compared to the later growth phase are listed in the second column and their broad role in the first, and those whose abundance was higher in the later growth phase compared to the early growth phase are listed in the fourth column and their broad role in the third.

High in the initial days		High in the final days	
Function indicated	KO	Function indicated	KO
TCA cycle	6-phospho-beta-glucosidase	Stress response	RNA polymerase sigma-70 factor
Prevention of cell lysis	serine transporter	Sensing	iron complex outermembrane receptor protein
Transport	two-component system, LytTR family, sensor kinase	Sensing	methyl-accepting chemotaxis protein
Anaerobic respiration	formate C-acetyltransferase		uncharacterized protein
Electron transfer	pyruvate-ferredoxin/ flavodoxin oxidoreductase	Transport	ATP-binding cassette, subfamily B
Transport	concentrative nucleoside transporter, CNT family	Electron transfer	glutathione S-transferase
Transport	putative transport protein	Lipid metabolism	3-oxoacyl-[acyl-carrier protein] reductase
Transport	MFS transporter, OPA family, sugar phosphate sensor protein UhpC	Amino acid biosynthesis	glutamine synthetase
Lipid biosynthesis	glycerol dehydrogenase	Lipid biosynthesis	acetyl-CoA C-acetyltransferase
Transport	C4-dicarboxylate transporter, DcuC family	Transport	putative ABC transport system permease protein

Table S6: KOs that have significantly higher abundances in the early and later growth phases in the glucose environment. Derived from 16S data using PICRUSt2, the KOs whose abundance was higher in the early growth phase compared to the later growth phase are listed in the second column and their broad role in the first, and those whose abundance was higher in the later growth phase compared to the early growth phase are listed in the fourth column and their broad role in the third.

High in the initial days		High in the final days	
Function indicated	Pathway	Function indicated	Pathway
Lipid biosynthesis	(Kdo)2-lipid A biosynthesis	Aerobic respiration	aerobic respiration I (cytochrome c)
Vitamin biosynthesis	thiazole biosynthesis II (aerobic bacteria)	Salvage	pyruvate fermentation to isobutanol (engineered)
Vitamin biosynthesis	menaquinol-6 biosynthesis I	Lipid biosynthesis	cis-vaccenate biosynthesis
Vitamin biosynthesis	menaquinol-9 biosynthesis	Lipid biosynthesi	gondoate biosynthesis (anaerobic)
Vitamin biosynthesis	menaquinol-10 biosynthesis	Amino acid biosynthesis	L-isoleucine biosynthesis II
Salvage	L-arginine and L-ornithine degradation	Amino acid biosynthesis	Valine synthesis
Salvage	L-arginine, putrescine, and 4-aminobutanoate degradation	Amino acid biosynthesis	Isoleucine synthesis
Electron transport	heme b biosynthesis from uroporphyrinogen-III	Lipid biosynthesis	fatty acid elongation – saturated
Antibiotic resistance	polymyxin resistance	Lipid biosynthesis	mycolate biosynthesis
Amino acid biosynthesis	chorismate metabolism	Lipid biosynthesis	oleate biosynthesis IV (anaerobic)

Table S7: Pathways that have significantly higher abundances in the early and later growth phases in glucose environment. Derived from 16S data using PICRUSt2, the pathways whose abundance was higher in the early growth phase compared to the later growth phase are listed in the second column and their broad role in the first, and those whose abundance was higher in the later growth phase compared to the early growth phase are listed in the fourth column and their broad role in the third.

High in the initial days	High in the final days
fermentation	aerobic chemoheterotrophy
nitrate reduction	nitrate respiration
	nitrogen respiration
	human pathogens all
	animal parasites or symbionts
	human associated
	sulfate respiration
	respiration of sulfur compounds
	intracellular parasites
	ureolysis

Table S8: Functions that have significantly higher abundances in the early and later growth phases. Derived from 16S data using FAPROTAX, the functions whose abundance was higher in the early growth phase compared to the later growth phase are listed in the first column, and those whose abundance was higher in the later growth phase compared to the early growth phase are listed in the second column.
