# Supplementary figures and images for: Diverse Microbial Communities Assemble on Both Recalcitrant and Labile Carbon Sources
Source: Environ Microbiol. 2026 Jun 25;28(7):e70351. doi: 10.1111/1462-2920.70351 (PMC13305152; doi:10.1111/1462-2920.70351)

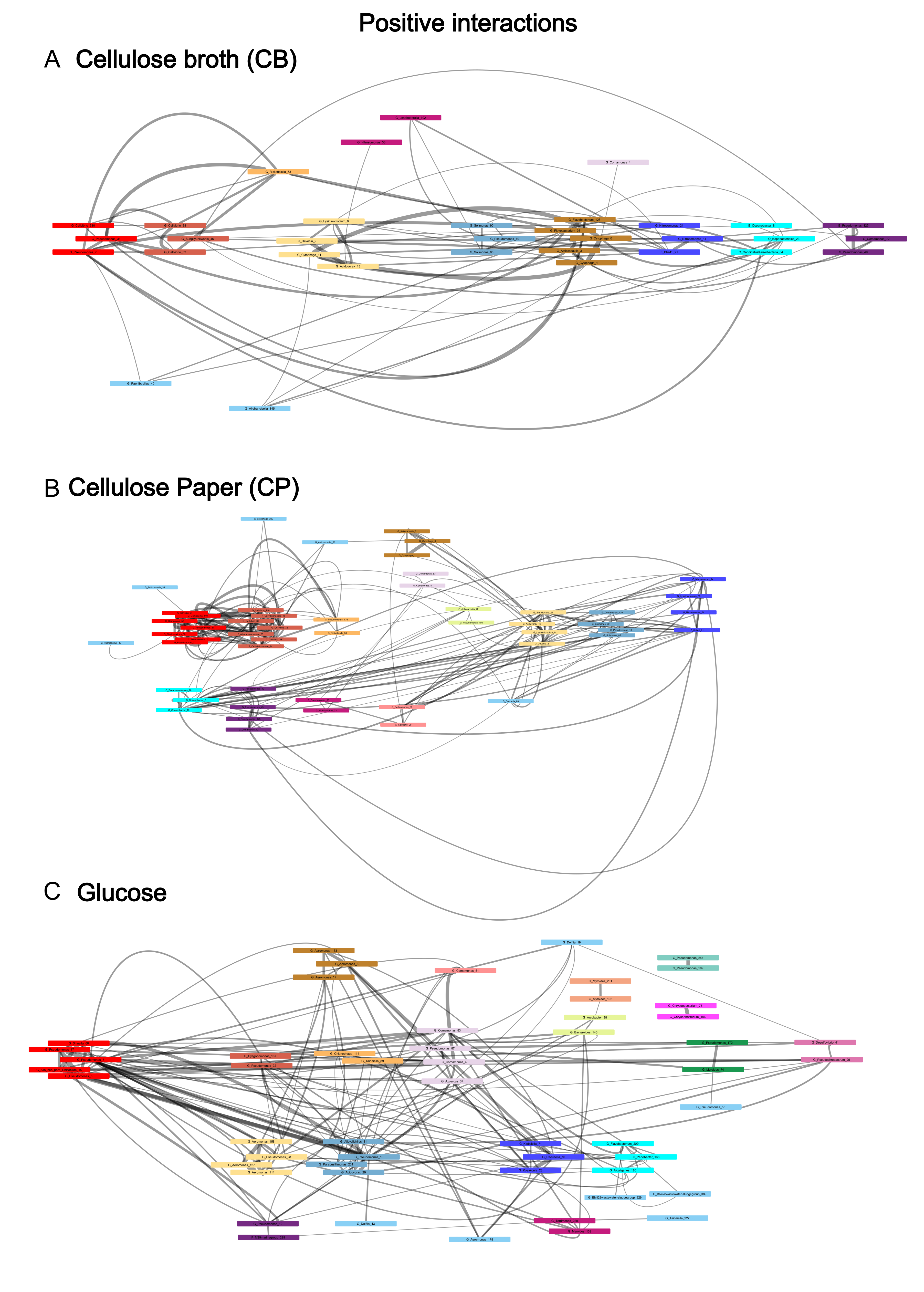

Supplement: Supplementary file 2 — File S1: Combined networks_modules_positive. [file EMI-28-e70351-s002.png]
